# Anatomically curated segmentation of human subcortical structures in high resolution magnetic resonance imaging: An open science approach

**DOI:** 10.3389/fnana.2022.894606

**Published:** 2022-09-30

**Authors:** R. Jarrett Rushmore, Kyle Sunderland, Holly Carrington, Justine Chen, Michael Halle, Andras Lasso, G. Papadimitriou, N. Prunier, Elizabeth Rizzoni, Brynn Vessey, Peter Wilson-Braun, Yogesh Rathi, Marek Kubicki, Sylvain Bouix, Edward Yeterian, Nikos Makris

**Affiliations:** ^1^Department of Psychiatry, Department of Neurology, Center for Morphometric Analysis, Athinoula A. Martinos Center for Biomedical Imaging, Massachusetts General Hospital and Harvard Medical School, Boston, MA, United States; ^2^Psychiatry Neuroimaging Laboratory, Brigham and Women’s Hospital, Boston, MA, United States; ^3^Department of Anatomy and Neurobiology, Boston University School of Medicine, Boston, MA, United States; ^4^School of Computing, Queen’s University, Kingston, ON, Canada; ^5^Surgical Planning Laboratory, Brigham and Women’s Hospital, Boston, MA, United States; ^6^Department of Psychology, Colby College, Waterville, ME, United States

**Keywords:** MRI, atlas, HOA, subcortical, open science, ontology, deep learning, segmentation (image processing)

## Abstract

Magnetic resonance imaging (MRI)-based brain segmentation has recently been revolutionized by deep learning methods. These methods use large numbers of annotated segmentations to train algorithms that have the potential to perform brain segmentations reliably and quickly. However, training data for these algorithms are frequently obtained from automated brain segmentation systems, which may contain inaccurate neuroanatomy. Thus, the neuroimaging community would benefit from an open source database of high quality, neuroanatomically curated and manually edited MRI brain images, as well as the publicly available tools and detailed procedures for generating these curated data. Manual segmentation approaches are regarded as the gold standard for brain segmentation and parcellation. These approaches underpin the construction of neuroanatomically accurate human brain atlases. In addition, neuroanatomically precise definitions of MRI-based regions of interest (ROIs) derived from manual brain segmentation are essential for accuracy in structural connectivity studies and in surgical planning for procedures such as deep brain stimulation. However, manual segmentation procedures are time and labor intensive, and not practical in studies utilizing very large datasets, large cohorts, or multimodal imaging. Automated segmentation methods were developed to overcome these issues, and provide high data throughput, increased reliability, and multimodal imaging capability. These methods utilize manually labeled brain atlases to automatically parcellate the brain into different ROIs, but do not have the anatomical accuracy of skilled manual segmentation approaches. In the present study, we developed a custom software module for manual editing of brain structures in the freely available 3D Slicer software platform that employs principles and tools based on pioneering work from the Center for Morphometric Analysis (CMA) at Massachusetts General Hospital. We used these novel 3D Slicer segmentation tools and techniques in conjunction with well-established neuroanatomical definitions of subcortical brain structures to manually segment 50 high resolution T1w MRI brains from the Human Connectome Project (HCP) Young Adult database. The structural definitions used herein are associated with specific neuroanatomical ontologies to systematically interrelate histological and MRI-based morphometric definitions. The resulting brain datasets are publicly available and will provide the basis for a larger database of anatomically curated brains as an open science resource.

## Introduction

Magnetic resonance imaging (MRI) has long been used to non-invasively visualize and quantify correlates of neuroanatomical structures (Filipek et al., 1989; Jouandet et al., 1989; Kennedy et al., 1989; Reyment and Bookstein, 1992; Fischl and Dale, 2000). Interregional differences in contrast and intensity have been used in conjunction with neuroanatomically based rules to determine the number of voxels belonging to each brain structure and thereby measure structural volumes (Filipek et al., 1994; Caviness et al., 1999). Since the inception of the principles by which imaging correlates of neuroanatomical structures are measured, volumetric and morphometric analyses of brain structures have become essential and frequently used approaches for evaluating the impact of neurological and psychiatric disorders on brain structure (Caviness et al., 1996; Breiter et al., 1997; Seidman et al., 1999; Goldstein et al., 2001, 2007; Herbert et al., 2003; Frazier et al., 2005; Rauch et al., 2006; Keuthen et al., 2007; Makris et al., 2008; Yang et al., 2014, 2015).

Magnetic resonance imaging-based volumetry was introduced in the late 1980s as a method to quantitatively characterize brain structures in humans (Caviness et al., 1989; Filipek et al., 1989; Kennedy et al., 1989). This method was based on a combination of the neuroanatomical systems approach of Deepak Pandya and colleagues (e.g., Pandya and Yeterian, 1985; Mesulam, 2000; Pandya et al., 2015) and the MRI-based morphometric approach pioneered by Caviness et al. (1999). As such, it provided a neuroanatomical framework to measure brain anatomy in health and disease (Rademacher et al., 1992; Filipek et al., 1994; Caviness et al., 1999; Makris et al., 1999). Pioneering studies in the mid-1990s, which defined brain structure regions of interest (ROIs) on MRI scans based on neuroanatomical principles, were conducted by the Center for Morphometric Analysis (CMA) at Massachusetts General Hospital. These investigations entailed the development of novel segmentation tools for MRI-based volumetric analysis (Rademacher et al., 1992; Filipek et al., 1994; Caviness et al., 1996, 1999), which were modified and incorporated into the custom designed software platform known as CardViews (Caviness et al., 1996, 1999; Meyer et al., 1999). This platform served as the basis for numerous MRI-based brain segmentation studies (e.g., Breiter et al., 1997; Kennedy et al., 1998; Seidman et al., 1999; Goldstein et al., 2001, 2007, 2010; Caviness et al., 2002; Fischl et al., 2002, 2004; Herbert et al., 2003; Makris et al., 2004, 2006; Frazier et al., 2005; Rauch et al., 2006; Keuthen et al., 2007; Yang et al., 2014, 2015). Many of the MRI-based neuroanatomical definitions implemented by the CMA (e.g., Filipek et al., 1994; Caviness et al., 1999; Makris et al., 1999) continue to serve as the basis for segmentation of subcortical structures by systems such as FreeSurfer, Neuromorphometrics, and Mindboggle as well as the Harvard-Oxford Atlas (HOA), a probabilistic atlas distributed with the FSL software platform (Fischl et al., 2002; Frazier et al., 2005; Klein et al., 2005, 2017; Klein and Hirsch, 2005; Desikan et al., 2006; Makris et al., 2006; Goldstein et al., 2007; Jenkinson et al., 2012). Importantly, the volumetrics framework developed by the CMA (Caviness et al., 1999), which incorporates the neuroanatomical systems approach of Pandya and Yeterian (1985), emphasizes the importance of anatomical accuracy in the delineation and measurement of subcortical and cortical brain structures (Rademacher et al., 1992, 1993; Makris et al., 1999; Rushmore et al., 2020a,b,c).

Several research groups have introduced atlases of the human brain (see Dickie et al., 2017, for review), but these atlases often have been based on low quality brain images, as compared to current technical standards, and on relatively small numbers of individuals. Moreover, the data and protocols used to generate the atlases are typically not shared or openly available. Recently, data sharing initiatives such as openneuro.org, the Open Access Series of Imaging Studies, the United Kingdom Biobank, the Canadian Open Neuroscience Platform, the Young Adult and Lifespan Human Connectome Project (HCP), disease-specific connectome projects, and the Adolescent Brain Cognitive Development (ABCD) project have provided an unprecedented amount of diverse data, including high-resolution MRI, to the neuroimaging community. The current, public availability of these high-quality data provides an opportunity to refine and improve the precision of neuroanatomical definitions as a basis for generating brain atlases using relatively large numbers of well-characterized subjects.

The creation of improved brain atlases depends on reassessment of the definitions of neuroanatomical structures. Anatomical definitions of MRI-based ROIs are generally accepted, but there continues to be a lack of clarity and consistency between the ontology of histologically defined individual brain structures and their corresponding ROIs as represented in MRI-based morphometric analyses. For example, the caudate nucleus ROI in an MRI may not correspond precisely to the anatomical definition of this structure. Similarly, the anatomical term pia does not necessarily correspond to the MRI-based term pia typically used to define the cerebral exterior (see Fischl et al., 2002; Makris et al., 2008). Although there are fundamental commonalities between the structural definitions and ontologies derived from neuroanatomy and MRI, there are frequent discrepancies and mismatches. These issues are present at the broader level of brain atlases, making comparisons between such atlases difficult. As pointed out by Bohland et al. (2009), different atlases use different anatomical terms to refer to the same anatomical structure. Moreover, a given anatomical term may be assigned to different anatomical regions in different atlases. Thus, there is a clear need to define ROIs using a common, established neuroanatomical lexicon in conjunction with detailed manuals for each atlas to insure consistent delineation of ROI borders. By combining detailed knowledge of human neuroanatomy with expertise in neuroimaging-based morphometric analysis, it is possible, in the context of recent advances in MRI technology, to more precisely relate MRI-based ROIs to the actual features of histologically defined structures (e.g., Amunts and Zilles, 2015).

Manual segmentation approaches continue to constitute the gold standard for brain segmentation and parcellation and currently underpin the construction of neuroanatomically accurate human brain atlases. In addition, neuroanatomically precise definitions of MRI-based ROIs derived from manual brain segmentation are essential for delineating accurate connections in structural connectivity studies, or for improving surgical planning for procedures such as deep brain stimulation. For instance, if a subcortical structure such as the caudate nucleus or the nucleus accumbens is segmented imprecisely, structural and connectional results will be inaccurate. However, manual segmentation procedures are time and labor intensive, and are typically precluded in studies that utilize large datasets (e.g., >1,000 cases; e.g., Schmaal et al., 2016; van Erp et al., 2016; Logue et al., 2018; van Rooij et al., 2018; Kong et al., 2020).

Automated brain segmentation methods (e.g., FreeSurfer, BrainVisa, volBrain, FSL, SPM, BrainSuite, Cat12; Cointepas et al., 2001; Shattuck and Leahy, 2002; Ashburner and Friston, 2005; Rivière et al., 2009; Ashburner, 2012; Fischl, 2012; Jenkinson et al., 2012; Manjón and Coupé, 2016) were developed for high data throughput, increased reliability, and multimodal imaging capability beyond what is possible using manual segmentation. These methods utilize manually labeled brain atlases to automatically parcellate the brain into different ROIs, but do not have the anatomical precision of manual segmentation approaches (Makris et al., 2008; Morey et al., 2009; Pardoe et al., 2009; Schoemaker et al., 2016; Guenette et al., 2018; Makowski et al., 2018; Monereo-Sánchez et al., 2021). Recently developed machine learning methods have revolutionized the fields of image classification and labeling and are being applied to perform automatic brain segmentation (Huo et al., 2019; Paschali et al., 2019; Roy et al., 2019; Coupé et al., 2020; Henschel et al., 2020) with the promise of more accurate results in a fraction of the computing time of classical segmentation tools. However, these recent methods require large amounts of data for training. Neuroanatomical inaccuracies and other errors may occur when these training data are supplied from naïve automated segmentation systems that often contain inaccurate neuroanatomy. Thus, both automated systems and machine learning techniques would benefit from a database of high quality, neuroanatomically curated and manually edited MRI brain images.

The creation of curated datasets as described in the current study requires software tools and well-defined methodologies that allow precise, neuroanatomically based morphometric analysis of MRIs. The 3D Slicer medical analysis and visualization software platform^1^ is one of the few software suites with advanced visualization capabilities that allows for precise manual segmentation of MRI datasets (Fedorov et al., 2012). In addition, it is open-source, supported by a large developer and user community, and designed specifically to allow customized extensions to its functionality. Accordingly, we developed a custom module for manual editing of brain structures in 3D Slicer (Rushmore et al., 2020d) that implements the techniques and user-interface tools developed by the CMA. The combination of new editing tools and the availability of high-resolution HCP data has led to improvements in the original CMA manual segmentation procedures and anatomical definitions. Therefore, in the present study, we used novel 3D Slicer segmentation tools and techniques in conjunction with neuroanatomically driven definitions of subcortical brain structures to manually segment 50 high resolution T1w MRI brains from the HCP Young Adult database. These structural definitions are associated with specific neuroanatomical ontologies to minimize the gap between histological and MRI-based morphometric definitions.

## Methods

### Subjects and imaging

Datasets were acquired from the HCP, as described in Glasser et al. (2013). ACPC-aligned T1w MRI images (0.7 × 0.7 × 0.7 mm voxel size) from the HCP Young Adult dataset were used. Fifty datasets were segmented (*n* = 25 female, *n* = 25 male), with the following demographic characteristics: 74% white, 20% Black or African American, 4% more than one race, 2% unknown or not reported. Fourteen percent of participants identified as Hispanic/Latino. The mean age of participants was 28.6 years (standard deviation = 10.8, min = 22, max = 35).

### Materials and equipment

Personal computers with pen and tablet capabilities were used to operate 3D Slicer, according to the specifications detailed in the system requirements section of 3D Slicer^1^. A custom-designed, freely available extension, the NeuroSegmentation module, was developed as part of this project as detailed below.

### Anatomic segmentation

Individual anatomic subcortical structures were segmented following the human HOA framework (Filipek et al., 1994; Caviness et al., 1999; Makris et al., 1999). In comparison to the original HOA, there were two major modifications. First, the software platform used was 3D Slicer rather than CardViews. 3D Slicer has the advantage of providing 3D visualizations of the anatomy of given brain structures with greater detail and clarity than CardViews, while also being actively developed and used by a large community. Second, neuroanatomical definitions were modified by senior neuroanatomists and applied to high quality HCP images. This process allowed for more precise delineation of structural borders, resulting in improved segmentation of subcortical brain structures. The anatomic definitions were updated as detailed below.

### Segmentation tools introduced in 3D slicer

**FIGURE 1:**
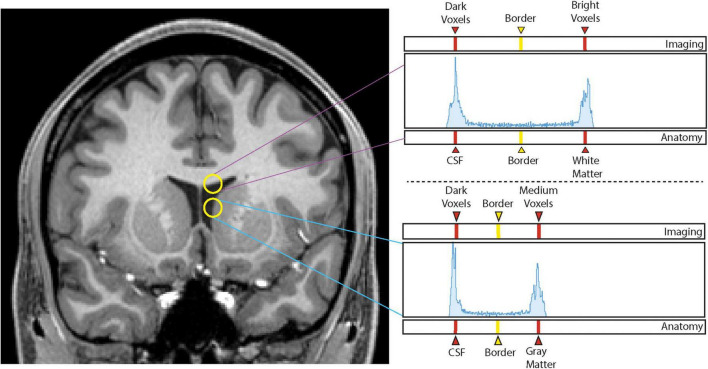
Histogram method. Sampling of voxel intensities between white matter and cerebrospinal fluid (upper yellow circle) produces a histogram (upper right panel) in which the left peak corresponds to dark voxels associated with cerebrospinal fluid (CSF) and the right peak corresponds to intense voxels of white matter. The vertical line equidistant between the two peaks represents the interpeak distance that produces the designated border between the two peaks, and thus, the two brain tissues. Sampling of voxel intensities using the lower circle produces the lower right histogram in which the left peak again corresponds to dark voxels of the CSF space, and the right peak to medium intensity voxels of the gray matter of the caudate nucleus. Notice there is now a different interpeak distance and a different border between these two structures.

**FIGURE 2:**
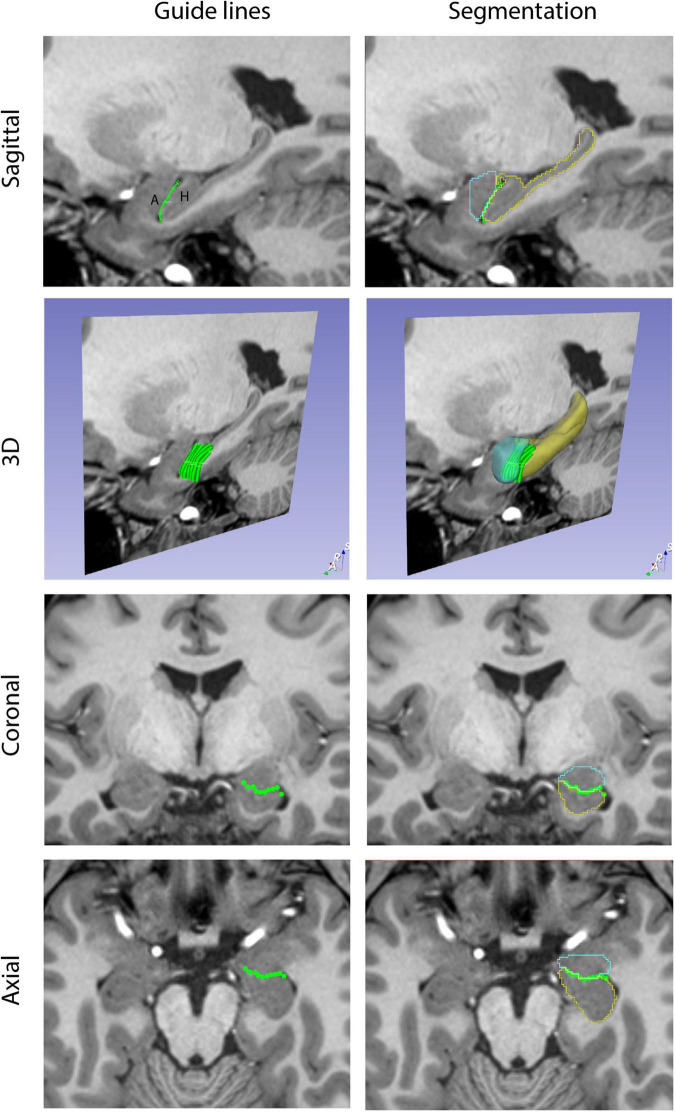
Guide Markup tool. The Guide Markup tool provides a means by which overlay lines can be drawn on one view of the brain (e.g., sagittal) to guide segmentation in another view (e.g., coronal). Guide lines are shown in the left column. In the upper left image, a guide line is drawn between the amygdala (A) and the hippocampal formation (H). This is repeated for a number of sagittal sections to produce a series of parallel lines in the 3D perspective. When viewed in the coronal and axial planes, the transects of these guide lines are visualized as individual dots that together generate an estimate of the border between the amygdala and hippocampal formation. The right column shows the resulting segmentations of these two structures (amygdala in teal, hippocampal formation in yellow) along with the guideline overlays.

A custom-designed segmentation procedure was developed to take advantage of the core segmentation capabilities of 3D Slicer (e.g., *paint, draw, erase*). In addition, two custom editing tools developed by the CMA were incorporated (Worth et al., 1998; Seidman et al., 2002). The first tool, the *Histogram tool* (Figure 1), was designed to measure voxel intensities in two adjacent regions (e.g., the lateral ventricle and the adjoining white matter; Worth et al., 1998). The peaks in the histogram correspond to the dominant voxel intensity for each structure, and the interpeak distance is selected and used as a threshold measure for standard 3D Slicer segmentation tools (e.g., *Paint*, *Draw*). The second tool, the *Guide Markup tool* (Figure 2), was developed to specify borders in one perspective (e.g., sagittal) to aid in segmentation in a different view (e.g., coronal). For instance, the border between the hippocampal formation and the amygdala is specified as a series of markup lines in sequential sagittal planes to assist in the delineation of the amygdala-hippocampal formation border in the coronal plane (Seidman et al., 2002). These novel tools were combined with standard image segmentation and visualization tools in a custom-designed, open-source Neurosegmentation^2^ extension within 3D Slicer (see text footnote 1). This module was further customized to support modern user interface devices including touchscreens, styluses, and multiple displays. It specifically incorporates the use of a pen and a tablet for segmentation to facilitate ease of use and improve time efficiency.

### Anatomic definitions

Brain structures were defined by using signal intensity borders specified by the histogram method as well as by neuroanatomical conventions and anatomically based operational definitions designed for clarity and reliability, as detailed below. A list of structures and related ontologies from the NeuroNames (NN; Bowden and Martin, 1995; Bowden and Dubach, 2003) schema are presented in Table 1 and described below.

**TABLE 1:** Regional volumes from 50 young adult subjects.

Region of interest (ROI)	Neuronames ID	Mean volume (cm^3^)	Standard deviation	Coefficient of variation
Lateral Ventricle Left	209	7.62	3.94	0.52
Lateral Ventricle Right	209	6.88	3.17	0.46
Transverse Cerebral Fissure (CSF)	28	0.92	0.21	0.23
Third Ventricle	446	0.64	0.23	0.36
Fourth Ventricle	509, 621	1.62	0.54	0.33
Fifth Ventricle	257	0.04	0.13	3.41
Nucleus Accumbens Left	277	0.68	0.12	0.18
Nucleus Accumbens Right	277	0.61	0.12	0.19
Caudate Left	226	4.26	0.47	0.11
Caudate Right	226	4.36	0.53	0.12
Putamen Left	230	4.99	0.66	0.13
Putamen Right	230	5.04	0.66	0.13
Globus Pallidus Left	231	1.60	0.24	0.15
Globus Pallidus Right	231	1.62	0.23	0.14
Brainstem	2052*	21.28	2.75	0.13
Thalamus Left	300*	7.97	0.87	0.11
Thalamus Right	300*	7.88	0.93	0.12
Inferior Horn of the Lateral Ventricle Left	222	0.58	0.17	0.29
Inferior Horn of the Lateral Ventricle Right	222	0.64	0.20	0.30
Hippocampal Formation Left	177	3.67	0.38	0.10
Hippocampal Formation Right	177	3.82	0.37	0.10
Amygdala Left	237	1.42	0.18	0.13
Amygdala Right	237	1.43	0.18	0.13
Optic Chiasm	459	0.10	0.04	0.39
Anterior Ventral Diencephalon Left	n/a	1.17	0.15	0.12
Anterior Ventral Diencephalon Right	n/a	1.14	0.15	0.14
Posterior Ventral Diencephalon Left	n/a	3.72	0.53	0.14
Posterior Ventral Diencephalon Right	n/a	3.67	0.50	0.14

Asterisks indicate instances where the operational definitions in the present approach does not precisely correspond to the ontology (see text for details).

#### The lateral ventricle (NN ID 209)

The lateral ventricle was segmented using two independent histogram-based borders–one bordering white matter structures such as the corpus callosum and fornix, and one with gray matter structures such as the caudate nucleus. The anterior horn of the lateral ventricle, defined as the space anterior to the interventricular foramen of Monro, is bounded by the cerebral white matter, the caudate nucleus, nucleus accumbens, septal nuclei, and the septum pellucidum. The interventricular foramen of Monro is included in the lateral ventricle segmentation as it passes between the fornix and the thalamus. The body of the lateral ventricle is bounded by the fornix medially, the thalamus inferiorly, the caudate laterally, and cerebral white matter superiorly. In sections posterior to the thalamus, the ventricle enlarges in the inferior direction at the atrium and subsequently narrows at the occipital (posterior) horn in more posterior sections. The inferior horn of the lateral ventricle is not included in the lateral ventricle segmentation, but instead is treated as a separate ROI. The border between the lateral ventricle ROI and the inferior horn of the lateral ventricle ROI is specified as the first coronal section of the atrium; anterior to this section, the inferior horn and the body of the lateral ventricle are separated by white matter and clearly differentiable. The occipital horn of the lateral ventricle may sometimes be observed as a space separate from the ventricular atrium; in these instances, the occipital horn appears as an isolated space lateral to the calcarine fissure. This disconnection presumably reflects a narrowing of the ependymal-lined canal below the resolution limit of the MRI scan rather than a discrete disconnection between the ventricular spaces.

High intensity voxels within the ventricles consistent with the position and size of the choroid plexus are included within the lateral ventricle segmentation. Such voxels are present from the atrium posteriorly to a few sections anterior to the interventricular foramen of Monro where tufts of choroid plexus envelop the anterior wings of the transverse cerebral fissure (TCF) (see below). Care is taken to include the choroid plexus, but not to intrude on the TCF, which is segmented separately.

#### The transverse cerebral fissure (NN ID 28)

The TCF is an anterior extension of the subarachnoid space of the quadrigeminal cistern, located superior to the midbrain tectum. In coronal sections at the level of the posterior thalamus, it is bordered by the midbrain inferiorly, the thalami laterally, and the fornices superiorly and encloses the suprapineal recess of the third ventricle. More anteriorly, the TCF is separated from the third ventricle by the thin membrane of the tela choroidea inferiorly, and by the thalami and fornices as noted above. In more anterior sections, as the thalami approach each other on the midline, the TCF adopts a diamond-shaped morphology, with the thalami bordering the lower blades of the diamond, and the fornices the upper. Superolateral extensions of the TCF may be observed between the flared lateral portions of the fornix and thalamus in the body of the lateral ventricle. At the level of the foramen of Monro, the TCF is split into two vertically oriented anterior wings before ending blindly in more anterior coronal sections. It should be noted that the TCF, as defined herein, corresponds to the anterior telodiencephalic portion of the TCF, as specified by Bowden, Swanson, Carpenter, and others (Carpenter and Sutin, 1983; Bowden and Martin, 1995; Bowden and Dubach, 2003; Swanson, 2015). This region is referred to herein as CSF to be consistent with the nomenclature developed by the CMA.

#### The third ventricle (NN ID 446)

The third ventricle is a thin midline cavity at the center of the forebrain. It receives cerebrospinal fluid from each lateral ventricle through the bilateral foramina of Monro, and communicates posteriorly with the cerebral aqueduct of Sylvius. On coronal sections, the third ventricle first appears as a horizontal space superior to the optic chiasm. In more posterior sections, this space enlarges superiorly to reach the inferior aspect of the anterior commissure, thereby producing a teardrop shape. In coronal sections more posterior to the anterior commissure, the foramina of Monro become visible. At this point, the hypothalamic sulci of Monro, bilateral evaginations in the third ventricle, appear on its lateral walls to confer a rhomboid shape to the third ventricle. These sulci are critical landmarks used to delimit the thalamus superiorly from the anterior and posterior portions of the ventral diencephalon inferiorly (see below). After the foramina of Monro close in more posterior sections, the velum interpositum appears to separate the TCF and the third ventricle. Similar to rules discussed in the lateral ventricle segmentation, the third ventricle segmentation extends through the choroid plexus associated with the velum to end in apposition to the inferior margin of the TCF. More posteriorly, the thalami approach each other to form the lateral and superior margins of the third ventricle. The third ventricle occupies this position until the appearance of the habenular nuclei, wherein the thin membrane of the tela choroidea will separate the superior margin of the third ventricle from the TCF above. The posterior commissure is present at the posterior end of the habenula and divides the third ventricle into two distinct spaces. The space inferior to the commissure is considered to be the cerebral aqueduct of Sylvius, a canal included in the fourth ventricle segmentation. The space superior to the commissure is the posterior extension of the third ventricle, the suprapineal recess. This extension is separated superiorly from the midbrain in coronal sections to be completely encapsulated by the TCF.

It should be noted that an extension of the third ventricle is anatomically present superior to the location at which the thalami converge along the midline. The variable anatomical convergence of the thalami along the midline, and their connection, is termed the interthalamic adhesion or massa intermedia. The superior extension of the third ventricle above the massa intermedia should be limited superiorly by the choroid plexus and tela choroidea; however, since the border of the third ventricle superior to the massa intermedia cannot be visualized, this portion of the third ventricle is operationally included in the TCF segmentation.

#### The fourth ventricle

The fourth ventricle, as defined here, includes both the fourth ventricle *per se* (NN ID 621) as well as the cerebral aqueduct of Sylvius (NN ID 509). The beginning of the fourth ventricle segmentation occurs when the posterior commissure appears on the coronal series. This space is segmented as it progresses inferoposteriorly through the midbrain to enlarge between the superior cerebellar peduncles in the caudal midbrain as the fourth ventricle. This space is bordered anteriorly by the brainstem and laterally and superiorly by cerebellar white matter. The inferior aspect of the fourth ventricle is the obex. Similar to the definitions of previous cerebral ventricular spaces, the choroid plexus is included in the fourth ventricle segmentation.

#### The caudate nucleus (NN ID 226)

The caudate nucleus has a comma-shaped morphology and flanks the lateral ventricle in the frontal, parietal, and temporal lobes. The anterior portion of the caudate is its largest region and is referred to as the head of the caudate. This caudate region is located anterior to the foramen of Monro and is bordered laterally by the internal capsule, superiorly by cerebral white matter, inferiorly by the nucleus accumbens, and medially by the anterior horn of the lateral ventricle. On coronal sections where the caudate is colocated with the putamen, gray matter often extends as cell bridges through the internal capsule to connect these two structures, a result of their shared phylogenetic origin. Given the positional variability of these cell bridges, they are not included in the segmentation of either nucleus. Posterior to the foramen of Monro, the body of the caudate nucleus becomes displaced superiorly to the lateral aspect of the body of the lateral ventricle by the imposition of the thalamus. The body of the caudate decreases in size from anterior to posterior. At the atrium or trigone of the lateral ventricle, the caudate nucleus turns inferiorly to adopt a vertical orientation, flanking the ventricular space laterally. The caudate then courses ventrally and anteriorly to the superior aspect of the inferior horn of the lateral ventricle, a region termed the tail of the caudate. While segmentation of the vertical portion of the caudate is performed in its entirety, the tail is not segmented due to its poor visualization on MRI scans.

The anterior borders of the head of the caudate nucleus are verified using markup lines placed on the sagittal and axial views. Attention is paid to the white matter region superior to the caudate at the superior lateral margin of the lateral ventricle to avoid inclusion of the occipitofrontal fascicle, a fiber bundle whose voxels may appear similar in intensity to gray matter (Makris et al., 2007). Since the inferior border of the caudate with the anterodorsal portion of the bed nucleus of the stria terminalis is often unclear and relates to the curvature of the thalamus and genu of the internal capsule, the gray matter region above the anterior commissure is included in the caudate segmentation. The stria terminalis, a poorly myelinated fiber bundle medial to the body of the caudate nucleus, is also incorporated in the caudate segmentation.

#### The nucleus accumbens (NN ID 277)

The nucleus accumbens, or nucleus accumbens septi, is a region of the striatum interposed between and contiguous with the caudate and putamen. The borders of the nucleus accumbens are defined operationally. The first coronal section of the nucleus accumbens is segmented when the small islands of the putamen coalesce into an identifiable sliver of gray matter lateral to the anterior limb of the internal capsule. A straight line is then drawn from the most inferior and lateral point of the anterior horn of the lateral ventricle to the inferior-most point of the putamen. The superior border of the nucleus accumbens lies along the length of this drawn line from the ventricle to the medial part of the internal capsule. The adjoining white matter, including the rostrum of the corpus callosum, comprises the inferior and medial borders of the nucleus accumbens. More posteriorly, where the anterior limb of the internal capsule rises and the nucleus accumbens and putamen become directly connected, the operational rule is adjusted so that the line drawn to delimit the superior border of the nucleus accumbens ends in the middle of the inferior aspect of the internal capsule. From here, a second line segment is drawn vertically and inferiorly to form the lateral border of the nucleus accumbens. As before, the interface of the nucleus accumbens with the white matter forms the inferior and medial borders of the nucleus accumbens. The nucleus accumbens is segmented until the anterior commissure begins to penetrate the lateral portion of the gray matter, at which point the nucleus accumbens segmentation is considered complete.

#### The putamen (NN ID 230)

The anterior portion of the putamen is located laterally to the internal capsule, and more posteriorly is bordered by the nucleus accumbens medially. The external capsule, a white matter bundle, borders the putamen laterally. Near the anterior commissure, the globus pallidus appears between the putamen and the internal capsule, a relationship that persists for much of the anterior-posterior extent of the putamen. For several coronal sections, the putamen is located beneath the globus pallidus; at these levels, the medial border of the putamen is considered to lie along a vertical line emanating from the lateral aspect of the anterior commissure. More posteriorly, a branch of the middle cerebral artery is frequently located below the putamen and forms an approximate inferior border. Following the disappearance of this blood vessel farther posteriorly, a tapered tail of the putamen extends inferiorly into the white matter that connects the frontal and temporal lobes at the frontotemporal junction, and is separated from the posterolateral margin of the amygdala by white matter. From here, the putamen diminishes further in size and terminates posteriorly. As previously discussed, thin gray matter bridges extend through the white matter of the internal capsule to join the putamen and caudate at intervals, but are not included in either segmentation. These striatal bridges are most evident anteriorly between the head of the caudate and the anterior portion of the putamen. These bridges are also seen between the body of the caudate and the superior portion of the putamen, between the tail of the putamen and the tail of the caudate, and between the posterior limit of the putamen and the vertical posterior portion of the caudate. These relationships are important for understanding the operationally defined borders of the putamen; none of these gray matter bridges are included in the segmentation of either the putamen or the caudate (see above).

#### The globus pallidus (NN ID 231)

The globus pallidus is located between the internal capsule and the putamen. It comprises two main segments, the pars internus and pars externus, which are frequently, but not always, visualized on the T1w scans. Anteriorly, the globus pallidus appears inferior to the medial and inferior aspect of the internal capsule and superior to the medial part of the anterior commissure. Its lateral border lies along the medial border of the putamen, a separation characterized by a thin white matter layer, the lateral lamina of the globus pallidus, which is included in the globus pallidus segmentation as a matter of definition. In more posterior coronal sections, the profile of the anterior commissure moves laterally, and the shape of the globus pallidus approximates a triangle, with the apex of the triangle pointed medially and slightly inferiorly. In more posterior sections, the triangle diminishes in size and the globus pallidus tapers and then ends. Axial sections are used to confirm the posterior end of the globus pallidus, which is often obscure on coronal sections.

#### The brainstem (NN ID 2052)

The brainstem includes three main subdivisions: the midbrain, pons, and medulla. The superior and inferior borders of this structure are operationally defined and rely on markup lines drawn on the midsagittal section. The superior border of the brainstem is established with a markup line linking the posterior aspect of the posterior commissure with the pre-pontine fissure in the interpeduncular fossa. The inferior border of the brainstem is defined by a line drawn between the anterior limit of the pyramidal decussation and the obex of the fourth ventricle. These sagittal lines define fiducial limits for the superior and inferior limits of the brainstem on the coronal plane. The posterior border of the brainstem is the fourth ventricle, and the lateral borders are delineated by specific markup lines drawn from the superior and inferior inflection points of the middle cerebellar peduncle (see Supplementary material). The posterior border of the midbrain tectal component of the brainstem lies at the junction between the inferior colliculus and the superior medullary velum.

#### The thalamus (NN ID 300)

The thalamus is a conglomeration of nuclei making up a majority of the diencephalic volume. It begins immediately posterior to the foramen of Monro, where it is bounded by the internal capsule laterally and by the third ventricle and TCF medially. Its inferior border is defined by the hypothalamic sulcus (of Monro). More posteriorly, the thalamus increases in size and becomes adjacent to the lateral ventricle superiorly. At its most posterior extent, the thalamus overhangs the tectum of the midbrain, is bordered by the hippocampus inferiorly and the fornix laterally and is surrounded by subarachnoid space medially.

The thalamus contains several nuclei, including the metathalamic nuclei (lateral geniculate nucleus, medial geniculate nucleus) appended to its posterior and inferior margin. These metathalamic nuclei are not included in the thalamic segmentation *per se* due to the inability of MRI segmentation to define precise borders based on intensity or gross anatomical landmarks. By convention, they are included in the ventral diencephalon segmentation (see below).

#### The ventral diencephalon

The ventral diencephalon is a segmentation that contains several gray and white matter structures. Thus, it is not given a single ontological designation, but instead represents a region inferior to the hypothalamic sulcus, posterior to the anterior commissure, and extending laterally to include the optic chiasm. It thus includes the hypothalamus and sublenticular extended amygdala anteriorly. In more posterior sections, the ventral diencephalon segmentation also includes the habenular nuclei superiorly and medially, the posterior commissure medially, the lateral and medial geniculate nuclei laterally and inferiorly, and the pretectum posteriorly. In addition, it includes the zona incerta, substantia nigra, subthalamic nucleus, fields of Forel, and the red nucleus in its posterior region. It borders the brainstem at the fiducial superior brainstem border detailed previously. The ventral diencephalon can be divided into anterior and posterior portions by a coronal plane positioned immediately posterior to the mammillary bodies.

#### The hippocampal formation (NN ID 177)

The hippocampal formation is a region of the cerebral cortex folded under the medial portion of the temporal lobe. It comprises several subdivisions, including regions of the cornu Ammonis (CA1–4), the dentate gyrus and the subiculum as well as white matter regions such as the alveus and the fimbria. For the purposes of the present segmentation, the medial border of the hippocampal formation is the medial curve of the hemispheric margin, which includes a portion of the parasubicular cortex. The hippocampal formation is bordered by temporal lobe white matter inferiorly and the amygdala anteriorly. The hippocampal formation bulges into the inferior horn of the lateral ventricle and, as such, is bordered by this space anteriorly, superiorly, and laterally. Posteriorly, it is bordered by the atrium of the lateral ventricle. In anterior sections, the hippocampal formation appears inferior to the amygdala. In more posterior sections, the inferior horn of the lateral ventricle separates the amygdala superiorly from the hippocampus inferiorly and medially. The medial portion of gray matter that hooks around the medial aspect of the inferior horn of the lateral ventricle represents the hippocampal-amygdalar transition area and is included in the hippocampal formation segmentation. The hippocampal formation is separated from the ventricular space by a capsule of white matter, which is included in the hippocampal formation segmentation. In anterior regions, the portion of the capsule bordering the ventricle and the anterior border of the hippocampal formation with the amygdala is referred to as the alveus. In more posterior regions, the superior and medial aspect of the capsule is known as the fimbria. The fimbria coalesces posteriorly into the fornix, the major efferent fiber bundle of the hippocampal formation. The fornix operationally is considered to begin at the coronal level of the posterior commissure; at this level the fornix is not segmented with the hippocampal formation.

It should be noted that in previous versions of the HOA, this ROI was referred to as the hippocampus (e.g., Filipek et al., 1994; Makris et al., 1999; Seidman et al., 1999, 2002). In the present study, this term has been replaced with the more precise term hippocampal formation.

#### The amygdala (NN ID 237)

The amygdala is a collection of nuclei (the amygdaloid nuclei or complex) in the anterior portion of the medial temporal lobe that together form a structure with the approximate shape of an almond. It is bordered anteriorly, inferiorly, and laterally by cerebral white matter of the temporal lobe. In more posterior sections, the amygdala is bordered inferiorly by the hippocampal formation and the inferior horn of the lateral ventricle. The enlargement of the hippocampal formation posteriorly displaces the amygdala superiorly where it tapers lateral to the optic tract. The interface of the amygdala with the hippocampal formation on coronal sections is resolvable by the placement of markup lines on the axial and sagittal views (see below). The anterior border of the amygdala with the temporal pole white matter in coronal sections is similarly resolved by demarcating markup lines on the sagittal and axial views, a procedure supplemented by the operational definition that the anterior-most coronal section of the amygdala does not extend to the cerebral exterior.

#### The inferior horn of the lateral ventricle (NN ID 222)

The inferior horn of the lateral ventricle is the temporal portion of the ventricle that extends from the atrium of the lateral ventricle posteriorly to the amygdala anteriorly. It is bounded by the hippocampal formation medially and inferiorly, the amygdala superiorly and anteriorly, and white matter elsewhere. In its middle to posterior regions, the inferior horn is separated from the subarachnoid space by a thin membrane of tela choroidea in the choroidal fissure. This membrane is attached to the thickened medial portion of the alveus, and to the fornix more posteriorly. The inferior horn often does not appear as a single continuous space due to the limits of resolution of MRI scans. Thus, the hippocampal formation often seems to contact the lateral wall of the inferior horn and subdivide it into separate components. As with the lateral ventricle segmentation above, choroid plexus in the inferior horn of the lateral ventricle is included in the segmentation of this structure.

#### The fifth ventricle (NN ID 257)

The fifth ventricle is a CSF-filled space between the septa pellucida that medially border the anterior horns of the lateral ventricles. It is also referred to as the terminal ventricle of the forebrain, the cavum septum pellucidum, or the cavum Vergae. This space is typically small or non-existent and normally does not have an evident means of communication with the cerebral ventricular system. However, it may appear enlarged in certain conditions such as chronic traumatic encephalopathy (Koerte et al., 2016).

#### The optic chiasm (NN ID 459)

The optic chiasm is a structure in which the axons of the optic nerves undergo a partial decussation. This structure begins at the first coronal section of the third ventricle and is no longer segmented when the optic tracts are identified in posterior sections.

The above definitions and segmentation protocols were assembled into a detailed segmentation manual along with region-specific visual summaries of the segmentation process (Supplementary material). The ICBM152 2009b symmetrical template brain was used for illustrative purposes in these resources (Fonov et al., 2009, 2011).

### Reliability and quality control

Inter-rater reliability of segmentation was performed using the Dice-Sorenson Overlap in twelve cases. Intra-rater reliability was determined in the same way in three brains. In addition, all segmentations were reviewed and quality-controlled by senior neuroanatomists (NM, EY).

### Asymmetry

Comparisons of volumes from left and right brain structures were performed using a symmetry coefficient (Galaburda et al., 1987; Filipek et al., 1994) corresponding to (L - R)/[0.5(L + R)]. Evaluation of significant differences from 0 was carried out using a one-tailed Student’s *t*-test, in which the alpha was corrected using the Bonferroni method.

### Data sharing

Data sharing is detailed in the Appendix.

## Results

**FIGURE 3:**
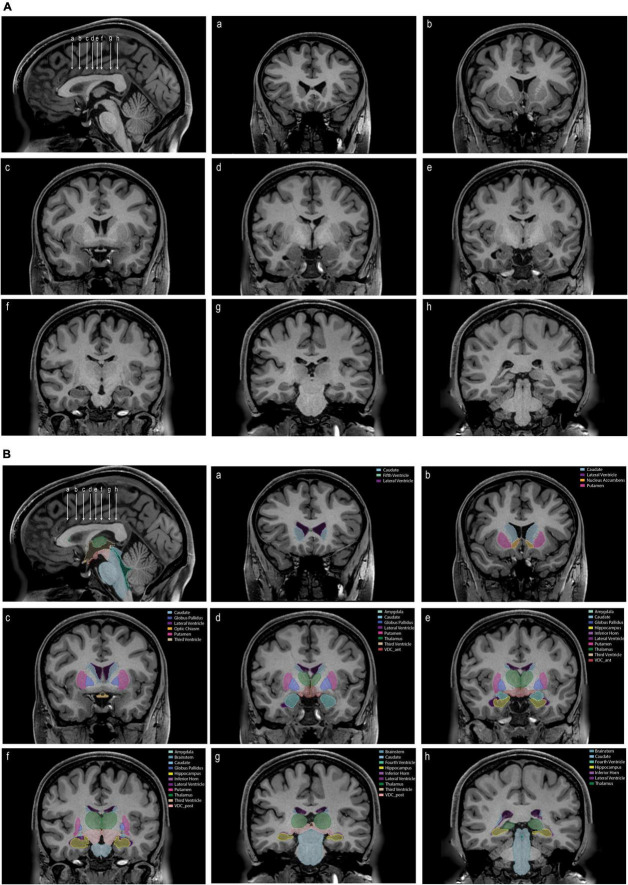
**(A)** An unlabeled sagittal image (upper left) showing the position of eight coronal sections **(a–h)**. **(B)** The same images as in panel **(A)** labeled with the tools and procedures specified in this paper. Each labeled structure is color coded. Abbreviations: VDC_ant, anterior ventral diencephalon; VDC_post, posterior ventral diencephalon.

Segmentation of subcortical structures was carried out using the methods described in the manual (Manual for Segmentation of Subcortical Brain Structures using 3D Slicer; Supplementary material), using segmentation tools in the NeuroSegmentation module of 3D Slicer (see text footnotes 1, 2). Segmentations are shown in Figure 3. Descriptive statistics for the subcortical volumes are shown in Table 1. Inter-rater and intra-rater reliability results are shown in Tables 2A,B.

**TABLE 2A:** Inter-rater reliability.

Region of interest (ROI)	Mean Dice	SD	Min	Max
Lateral Ventricle Left	0.95	0.02	0.92	0.98
Lateral Ventricle Right	0.95	0.02	0.93	0.98
Third Ventricle	0.84	0.05	0.75	0.90
Fourth Ventricle	0.87	0.04	0.80	0.94
Nucleus Accumbens Left	0.84	0.04	0.78	0.89
Nucleus Accumbens Right	0.84	0.05	0.76	0.93
Caudate Left	0.93	0.01	0.91	0.96
Caudate Right	0.93	0.02	0.88	0.96
Putamen Left	0.93	0.02	0.91	0.99
Putamen Right	0.93	0.02	0.91	0.99
Globus Pallidus Left	0.83	0.04	0.76	0.90
Globus Pallidus Right	0.81	0.06	0.73	0.90
Brainstem	0.95	0.01	0.94	0.98
Thalamus Left	0.88	0.04	0.78	0.92
Thalamus Right	0.88	0.03	0.82	0.93
Ventral Diencephalon Left	0.88	0.02	0.84	0.92
Ventral Diencephalon Right	0.88	0.01	0.85	0.90
Inferior Horn of Lateral Ventricle Left	0.72	0.05	0.61	0.82
Inferior Horn of Lateral Ventricle Right	0.72	0.05	0.61	0.81
Hippocampal Formation Left	0.87	0.03	0.82	0.90
Hippocampal Formation Right	0.87	0.02	0.82	0.90
Amygdala Left	0.84	0.03	0.78	0.88
Amygdala Right	0.80	0.05	0.71	0.88
Fifth Ventricle	0.76	0.07	0.65	0.84
Optic Chiasm	0.74	0.15	0.54	0.95

**TABLE 2B:** Intra-rater reliability.

Region of interest (ROI)	Mean Dice	SD	Min	Max
Lateral Ventricle Left	0.95	0.02	0.93	0.97
Lateral Ventricle Right	0.96	0.03	0.93	0.98
Third Ventricle	0.89	0.04	0.84	0.91
Fourth Ventricle	0.90	0.03	0.87	0.93
Nucleus Accumbens Left	0.87	0.04	0.82	0.89
Nucleus Accumbens Right	0.89	0.02	0.87	0.91
Caudate Left	0.93	0.03	0.91	0.96
Caudate Right	0.94	0.02	0.92	0.95
Putamen Left	0.94	0.02	0.92	0.95
Putamen Right	0.94	0.02	0.91	0.95
Globus Pallidus Left	0.81	0.05	0.77	0.86
Globus Pallidus Right	0.80	0.05	0.76	0.85
Brainstem	0.96	0.01	0.95	0.97
Thalamus Left	0.91	0.01	0.91	0.92
Thalamus Right	0.91	0.03	0.88	0.93
Ventral Diencephalon Left	0.90	0.01	0.89	0.91
Ventral Diencephalon Right	0.90	0.01	0.89	0.91
Inferior Horn of Lateral Ventricle Left	0.76	0.09	0.68	0.86
Inferior Horn of Lateral Ventricle Right	0.80	0.04	0.75	0.83
Hippocampal Formation Left	0.90	0.02	0.89	0.93
Hippocampal Formation Right	0.90	0.05	0.85	0.94
Amygdala Left	0.84	0.07	0.77	0.91
Amygdala Right	0.84	0.07	0.76	0.90
Fifth Ventricle	0.75	0.08	0.66	0.83
Optic Chiasm	0.87	0.18	0.66	0.99

The resolution and signal quality of the HCP data allowed for a high level of detail to be visualized beyond that typically observed in MRI scans *in vivo* for research or clinical purposes. For instance, the fifth ventricle (cavum septum pellucidum) was observed in 96% (48/50) of cases. Similarly, the posterior horn of the lateral ventricle was found to be disconnected from the atrium in many cases. In only 30% (15/50) of the cases did both posterior horns of the lateral ventricle clearly communicate with the atrium. In 30% (15/50) of cases both posterior horns were disconnected. In 40% (20/50) of cases, one lateral ventricle was disconnected such that in 16% (8/50) of cases, the left but not the right lateral ventricle was disconnected, and in 24% (12/50) of cases, the right but not the left was disconnected.

**FIGURE 4:**
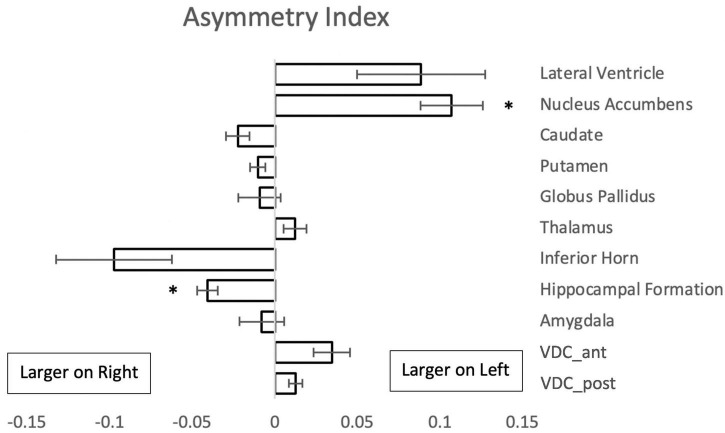
Asymmetry index {(L - R)/[0.5(L + R)]} for paired subcortical structures from 50 subjects. Negative values indicate a larger volume of the right structure. Asterisks signify a significant difference (*p* < 0.01 with Bonferroni correction for multiple comparisons); error bars represent standard error of the mean. Abbreviations: VDC_ant, anterior ventral diencephalon; VDC_post, posterior ventral diencephalon.

Comparison of the volumes of the subcortical brain structures between the left and right sides of the brain showed left-right asymmetry in two regions (Figure 4). The nucleus accumbens was larger on the left, whereas the hippocampal formation showed greater volume on the right side.

**FIGURE 5:**
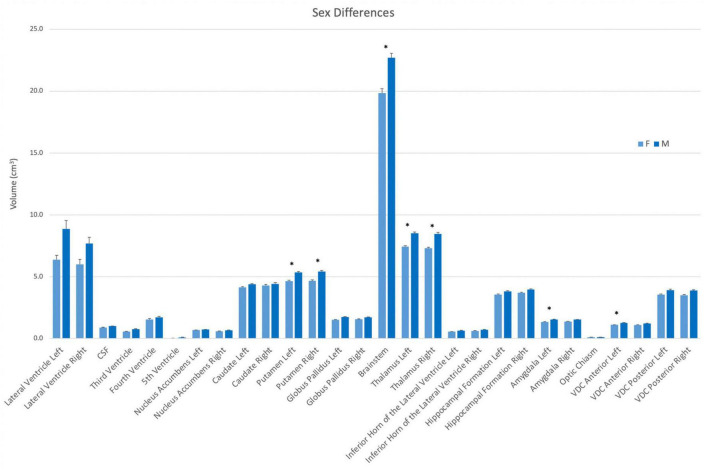
Volumes of each structure in the present dataset (*n* = 50) according to sex (female–light blue; male–dark blue). Asterisks signify a significant difference (*p* < 0.01), with multiple comparisons controlled for by Bonferroni *post-hoc* comparisons. Error bars represent the standard error of the mean. Abbreviations: CSF, cerebrospinal fluid space in the transverse cerebral fissure (TCF); ILV, inferior horn of the lateral ventricle; VDC, ventral diencephalon.

Volumes were also evaluated according to sex. In this analysis, the left and right putamen, the left and right thalamus, the brainstem, the left amygdala and the left anterior VDC were larger in men than women (Figure 5).

## Discussion

In this study, we developed a novel software tool for manual segmentation, implemented as a module in the open-source 3D Slicer software platform. This module incorporates principles and tools created by the CMA at Massachusetts General Hospital. These tools were used in conjunction with refined neuroanatomical definitions based on the human HOA, to manually segment selected subcortical structures in 50 high resolution T1w MRI datasets from the HCP Young Adult database. Moreover, the present neuroanatomically driven delineations of subcortical ROIs for MRI analysis were explicitly related to discrete ontological terminology to more systematically reflect the relationships between MRI-based morphometric and histological neuroanatomical definitions. The neuroanatomical definitions and the specific procedures used to generate subcortical segmentations were incorporated into a detailed manual. This manual provides a high level of definitional and methodological transparency for segmenting each defined brain structure. The present datasets of anatomically curated brains have been made publicly available and represent the initial release of a larger database that will be expanded on an ongoing basis. Furthermore, the tools and definitions developed in this study can be refined to produce more precise neuroanatomical ROIs as technology improves.

### The importance of manual segmentation of brain structures

Manual and semi-automated editing methods comprise the gold standard for precise neuroanatomical segmentation. These approaches are used to perform highly accurate anatomical segmentation of brain structures by incorporating expert neuroanatomical knowledge. The CMA was among the first laboratories to develop such an approach to brain structure analysis in MRI datasets. The CMA developed a software suite called CardViews for manual and semi-automated segmentation of brain structures. This software suite provided tools to enable segmentation based on neuroanatomical accuracy and was used to validate segmentation of subcortical brain structures, including those currently used by automated systems such as FreeSurfer (Fischl et al., 2002). Although manual and semi-automated editing approaches afford a high degree of neuroanatomical precision, such editing is time and labor intensive, which limits the efficient production of high throughput data.

The advent of automated segmentation of brain structures made high throughput and low cost analysis of MRI datasets possible. Automated systems utilize computational models to estimate the borders and volumes of neuroanatomical structures (e.g., Fischl et al., 2002, 2004; Huo et al., 2019; Paschali et al., 2019; Roy et al., 2019; Coupé et al., 2020; Henschel et al., 2020). However, such estimates are not necessarily accurate representations of the underlying neuroanatomy.

An optimal balance between throughput and neuroanatomical accuracy has not yet been achieved. Recent technological developments such as deep learning provide the opportunity to improve the quality and accuracy of automated segmentation results. To accomplish this goal, these methods require large, high-quality and well-curated training datasets to (1) provide specific control datasets that match the populations of interest in clinical and basic neuroimaging studies, and (2) reflect the demographic characteristics of the general population. These curated datasets can be utilized to train automated segmentation algorithms that may then be deployed to segment extremely high numbers of datasets with greater anatomical accuracy. Thus, the approach described herein using 3D Slicer is expected to provide the capability to curate brain segmentations and to improve neuroanatomical accuracy in high throughput analyses of neuroimaging data. In the current study, this capability has been advanced through the implementation of the HOA subcortical framework using newly developed 3D Slicer modules for structural segmentation of HCP high resolution T1w datasets.

### Subcortical volumetric segmentation using the Center for Morphometric Analysis Harvard-Oxford Atlas

The current volumetric results for subcortical structures are comparable to previously reported findings from the CMA (Table 3; Filipek et al., 1994; Makris et al., 1999; Seidman et al., 1999, 2002). It should be noted that the volumes of brain structures as reported previously by the CMA differ to varying degrees from those in the present study. These differences are a result of both improved anatomical resolution on MRI scans and refined neuroanatomical definitions and curation.

**TABLE 3:** Comparison of subcortical structural volume estimates from Center for Morphometric Analysis (CMA) studies.

Region	Filipek et al., 1994 (*n* = 20)	Makris et al., 1999 (*n* = 20)	Seidman et al., 1999 (*n* = 26)	Present study (*n* = 50)
Lateral Ventricle	18.1	n.d.	14.1	14.5
Third Ventricle	1.3	n.d.	0.7	0.6
Fourth Ventricle	1.9	n.d.	1.1	1.6
Lentiform	19.6	19.51	17	19.9
Caudate	9.5	7.83	7.7	8.6
Putamen	10.1	10.23	9.3	10
Nucleus Accumbens	n.d.	1.45	n.d.	1.3
Pallidum	3.9	3.97	2.9	3.2
Brainstem	23.4	n.d.	23.8	21.3
Hippocampus	9.9	12.54	9.8	7.5
Amygdala	5.5	2.97	5.5	2.9
Central Gray	20.5	20.14	n.d.	25.6
VDC	n.d.	5.47	n.d.	9.7
Thalamus	n.d.	14.67	14	15.8

n.d., not determined.

Filipek et al. (1994) were the first to define many subcortical regions as ROIs on the brain MRI, including components of the ventricular system, the caudate nucleus, the putamen, the pallidum, the brainstem, and the hippocampus. Diencephalic areas such as the thalamus and more ventral nuclear regions were combined into a single central gray ROI. Subsequent studies (Makris et al., 1999; Seidman et al., 1999, 2002) used similar procedures for segmenting many of the same subcortical ROIs and provided more refined procedures and definitions as certain other structures were emphasized and incorporated. For instance, the central gray ROI was divided into a thalamic ROI and a ventral diencephalic ROI, and the nucleus accumbens was extracted as a separate ROI from the caudate and putamen ROIs of Filipek and colleagues (Filipek et al., 1994; Makris et al., 1999; Seidman et al., 1999). For other structures, definitions were modified to increase neuroanatomical precision. Specifically, the amygdala as defined by Filipek explicitly included the nucleus basalis of the basal forebrain and was separated from the hippocampus using a coronal plane positioned at 10/24th of the distance between the anterior and posterior commissures (Filipek et al., 1994). Later studies with improved imaging resolution and signal quality used precise neuroanatomical separation between the amygdala and hippocampus to refine their borders (Seidman et al., 2002). In general, these later studies reported ROI volumes of similar or smaller size when compared to the corresponding ROI volumes of Filipek et al. (1994) (Table 3). Differences in neuroanatomical definitions, in the sample size, and possibly in the age of the subjects may account for some of the dissimilarities in volumetric results among these studies.

The current study utilized anatomical definitions and segmentation procedures similar to those developed originally by the CMA (e.g., Filipek et al., 1994; Makris et al., 1999; Seidman et al., 1999, 2002), but found consistently lower ROI volumes (Table 3). These differences in ROI volumes are likely due to significantly improved image quality. The spatial resolution of the HCP T1w images reflects an approximately fourfold increase from the images segmented in previous studies from the CMA. This greater resolution allows for improved visualization and delineation of finer borders that previously would have been obscured by large voxels, thus yielding larger ROI volumes. These confounding partial volume effects can occur where multiple tissue classes are contained inside a single voxel. Thus, including such a voxel may erroneously inflate the ROI volume by assigning to that ROI a tissue class that does not belong to it.

### Refinements of neuroanatomical definitions in the present study

**FIGURE 6:**
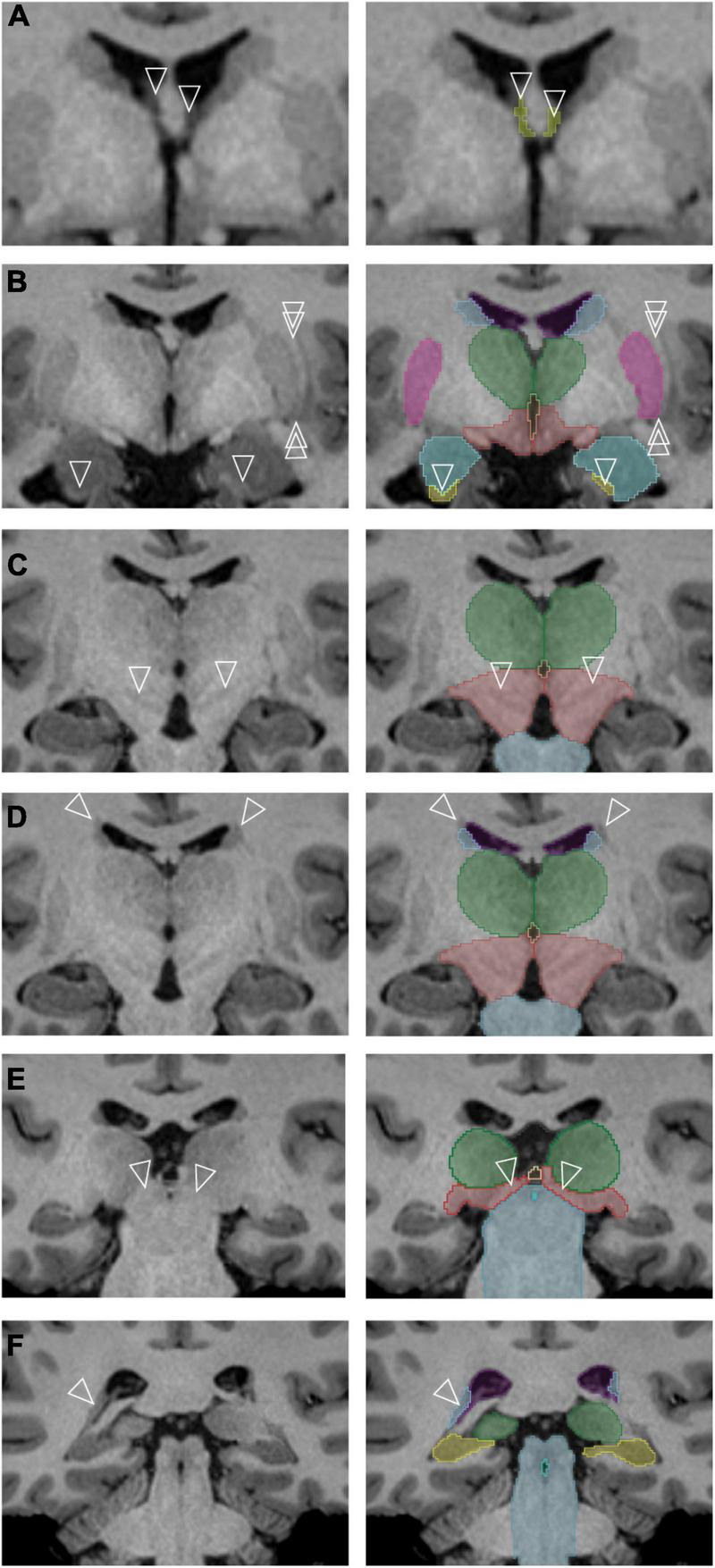
Specific subcortical regions for which refinements of the neuroanatomical definitions originally used by the Center for Morphometric Analysis (CMA) were made. The left column contains unlabeled images, and the right column represents the corresponding images after segmentation. **(A)** The anterior portions of the transverse cerebral fissure (TCF) (arrowheads) are segmented as they separate along the columns of the fornix [cerebrospinal fluid (CSF) space of the TCF shown in yellow]. **(B)** The single arrowheads point to the anterior sliver of the hippocampal formation, definable by its white matter capsule. The double arrowheads indicate a thin white matter pathway that forms the lateral border of the putamen (pink) and is clearly observed in this dataset. The amygdala is shown in teal; the caudate, light blue; hippocampal formation, yellow; thalamus, green; ventral diencephalon (anterior portion), red. **(C)** Gray matter regions in the posterior ventral diencephalon (light red) are visible in the higher resolution dataset (arrowheads). The thalamus is shown in green; brainstem, light blue. **(D)** A white matter pathway superior and lateral to the caudate nucleus (blue) is indicated by the tips of the arrowheads. The thalamus is shown in green; ventral diencephalon (posterior portion), light red; brainstem, light blue. **(E)** The pretectal region (tips of arrowheads) is now included in the posterior portion of the ventral diencephalon (light red) rather than the brainstem (light blue). The thalamus is shown in green; third ventricle, beige. **(F)** The posterior bend of the caudate nucleus (tips of arrowheads, light blue) is identified adjacent to the atrium of the lateral ventricle (purple). The brainstem is shown in blue-gray, the; hippocampal formation in yellow, the fourth ventricle in aquamarine and the thalamus in green.

To the best of our knowledge, this is the first study to revise anatomical definitions of subcortical brain structures by leveraging high resolution HCP MRI data. Several examples, as discussed below and shown in Figure 6, illustrate revisions of established neuroanatomical definitions in the present study:

1. The TCF ROI is better visualized and defined as containing anterior wings that extend on either side of the columns of the fornix (Figure 6A).
2. The encapsulating white matter of the hippocampal formation ROI could be clearly visualized in the high-resolution dataset, allowing for a highly precise anterior border of the hippocampal formation ROI to be delineated (Figure 6B, arrowheads).
3. The lateral border of the putamen ROI is now clearly separate from the external capsule and the claustrum. The discrete separation of the putamen from the posterior amygdala could also be visualized, enabling more systematic segmentation of the ventral portion of the putamen (Figure 6B, double arrowheads).
4. The structural heterogeneity of the posterior portion of the ventral diencephalon ROI could be observed in many datasets, allowing for the adoption of a more superiorly located border for this structure (Figure 6C).
5. A clear distinction of the occipitofrontal fascicle from the caudate nucleus ROI (Makris et al., 2007) at the superolateral border of the lateral ventricle could be observed in most brains (Figure 6D).
6. The anterior border of the brainstem ROI excludes the pretectum, whereas the inferior border adopts a definition based on anatomical landmarks rather than a transverse brainstem plane (Figure 6E).
7. The bend of the caudate nucleus ROI posteriorly along the anterolateral margin of the ventricular trigone could be visualized with certainty in the HCP data, and the voxels of this region are now included in the caudate nucleus ROI (Figure 6F).

**FIGURE 7:**
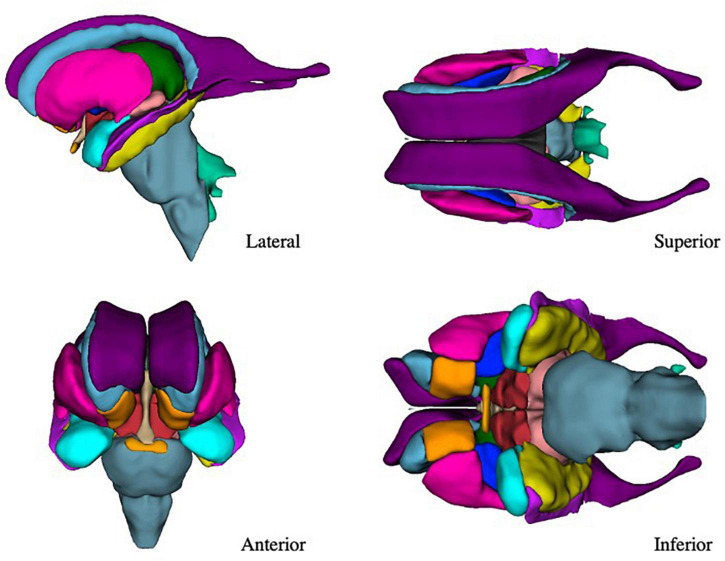
Three-dimensional view of the subcortical regions of interest (ROIs) from the lateral, anterior, superior, and inferior perspectives. Purple–lateral ventricle, beige–third ventricle, aqua–fourth ventricle, light green–fifth ventricle, orange–nucleus accumbens, blue–caudate nucleus, pink–putamen, dark blue–globus pallidus, blue-gray–brainstem, green–thalamus, red–anterior ventral diencephalon, light red–posterior ventral diencephalon, light purple–inferior horn of the lateral ventricle, yellow–hippocampal formation, light blue–amygdala, light orange–optic chiasm.

The present refinements in the CMA ROI definitions, enabled by the increased resolution of the HCP images, have allowed for a greater level of visualization and anatomical precision in the subcortical segmentations (Figure 7).

### The importance of open science in morphometric studies

The HCP has provided a major advance for open science in neuroimaging by providing well-characterized neuroimaging datasets (Glasser et al., 2013; Van Essen et al., 2013). This has led to increased transparency, comparability, and reproducibility of results from morphometric and other types of neuroimaging analyses (e.g., Bohland et al., 2009). This approach is representative of a growing trend of releasing neuroimaging primary data (e.g., ABCD, Lifespan, United Kingdom biobank, ABIDE, ADNI, CONP, OpenNeuro). The present study contributes to this initiative by providing open access to curated segmentation datasets, a detailed segmentation manual that describes the context of the theoretical and neuroanatomical approaches used to develop them, and an actively maintained 3D Slicer software segmentation module that implements these approaches. Specifically, the anatomically curated segmentation results of the 50 HCP datasets included in this study are expected to provide a basis for the creation of atlases by the neuroimaging community and are being expanded continuously with additional segmented brains. In addition, the software tools developed for manual brain segmentation in 3D Slicer are generalizable and may be used to refine or generate new ROIs by any researcher pursuing structural neuroimaging.

To our knowledge, this is the first time that segmentation methodology and software tools have been explicitly provided along with the data they produced. In previous approaches, methods have been provided (e.g., by the CMA), but the tools were not publicly available. Alternatively, segmentation tools have been provided, but not combined with explicit instructional methods for assessing neuroanatomical accuracy and producing precise segmentations of brain ROIs. For instance, the procedures for manual anatomical segmentation developed at the CMA were highly explicit and publicly available, but the full software platform used to segment brain ROIs was limited to and used only by the CMA. This situation made it difficult for segmentation to be performed using the same procedures across different research groups. By contrast, automated brain segmentation systems (e.g., FreeSurfer, SPM, FSL, Mindboggle) have made software available to the broader research community. However, these automated systems do not provide explicit neuroanatomical and technical training to evaluate the anatomical accuracy of MRI based segmentation. Furthermore, the manual segmentation tools within these systems have relatively limited editing capabilities. Here we provide both neuroanatomical and technical training materials (e.g., manuals; see Supplementary material) in conjunction with novel, freely available software that contains manual editing tools to enable more refined, anatomically rigorous, and precise brain segmentation.

The use of common, explicit neuroanatomical definitions is a critical aspect of open science that helps to promote consistency among brain research groups. These definitions should be grounded in fundamental neuroanatomy such that manual segmentations across laboratories are explicitly comparable and, ideally, based on a common ontological framework (Bowden et al., 2012). The present study provides such neuroanatomical definitions of several subcortical ROIs. Coupled with the aforementioned software, segmentation tools and training materials, these resources will help promote comparability and consistency in the interpretation of results among research groups.

### Curated brain templates for automatic segmentation

The advent of deep learning techniques has led to a paradigm shift in medical image analysis and in computer assisted intervention. Brain segmentation and parcellation, in particular, can benefit greatly from machine learning technology, leading to automated parcellation tools with unprecedented precision and speed (e.g., Huo et al., 2019; Coupé et al., 2020; Henschel et al., 2020). One major factor influencing the success (or failure) of such unsupervised learning algorithms is the availability of large datasets of carefully curated training data. Thus, our effort has the potential to become a critical resource for computer scientists interested in developing the next generation of segmentation algorithms. It should be pointed out that the 50 anatomically curated brains presented herein constitute the initial dataset of a larger cohort of labeled brains to be used as an open science resource.

### Relevance of precise anatomical segmentation to basic and clinical neuroscience

Neuroanatomical segmentation is an essential first step in both structural and functional imaging. Delineation of brain structure ROIs is the defining aspect for any study of localization in the human brain, which applies both to structural (e.g., morphometric, connectional) and functional or metabolic (e.g., fMRI, PET) studies. Inaccurate localization of ROIs can lead to errors in any kind of neuroimaging study. For example, inclusion of thalamic ROI voxels that are actually in the internal capsule has led to erroneous measurements of the thalamus and the corticospinal tract. Such errors result in a smaller corticospinal tract, which in turn leads to misinterpretation of the extent and connectivity of neural structures associated with the motor system and movement disorders (e.g., Dalamagkas et al., 2020). Another critical aspect of ROI localization relates to understanding the results of functional connectivity studies. Since structural connectivity is often fine-grained and systematically related to architectonic organization that underpins functional systems (e.g., Pandya and Yeterian, 1985; Schmahmann and Pandya, 2006; Pandya et al., 2015), exact segmentation is crucial for elaborating precise connections in the brain. Any error in structural neuroanatomical segmentation of ROIs may result in false negatives and positives in both functional and structural connectivity. Moreover, these errors may lead to misinterpretations and confusion in the specification of neural systems in basic and clinical neuroimaging studies. Thus, accurate, precise segmentation of ROIs is the bottleneck in many structural or multimodal neuroimaging studies (Worth et al., 1998). This applies not only to volumetry, but also to studies of brain structures based on shape analysis (e.g., Reyment and Bookstein, 1992; Caviness et al., 1999). In the words of Brodmann (1999), “Anyone wishing to undertake physiological localisational studies will thus have to base his research on the results of histological localization (p. 267).”

Improving the neuroanatomical precision of automated methodologies in neuroimaging research will help reduce the gap between neuroimaging and neuroanatomy. Precise segmentation will affect quantitative measurements of brain structures, which will then have significant impact in any clinical study using neuroimaging methods. Furthermore, it will elucidate with greater anatomical precision aspects of the brain such as cerebral dominance and structural laterality, structural sexual dimorphism and changes in development and aging. The present investigation was conducted with these broader goals in mind.

### Importance of ontological classification in brain science

Ontology in the present context of structural neuroanatomy is a formal means of defining and delineating brain structures that highlights their interrelationships. Ideally, any ontology should encapsulate relationships of brain structures across species. Thus, ontology is important for translational neuroscience because a comparative perspective is critical for understanding how experimental animal findings can be applied to the human brain. Specifically, for experiments involving subcortical nuclei in non-human primates, accurate translation of the findings to human brain and behavior requires precise correspondence between human and non-human primate brain structures. This correspondence should be precise and formal, based on a universal ontology for neuroanatomical structure [e.g., Neuronames (Bowden and Martin, 1995; Bowden and Dubach, 2003; Swanson, 2015]. In more practical terms, ontological coherence and accuracy between non-human primate and human brains is necessary for validating the wiring diagram of the human brain (e.g., Rushmore et al., 2020a). In two recent publications by our group, we have elaborated in detail how critically our understanding of the human brain is based on comparative data (Rushmore et al., 2020a,b). More broadly, any studies of structural connectivity depend on anatomically accurate, precise segmentation and parcellation of subcortical and cortical ROIs.

Imaging of neuroanatomical structures raises a fundamental ontological question, namely, how an imaged structure relates to the corresponding histologically defined, anatomic structure. For instance, how does a structure such as the caudate nucleus ROI as visualized using MRI relate to the histologically defined caudate nucleus? These two distinct representations of a specific anatomical structure differ in terms of (1) the methodologies and technologies applied to visualize them, (2) their assigned borders, (3) their visual composition (e.g., voxels vs. stained cellular or acellular material), and (4) the potential for visualization artifacts (e.g., imaging artifacts such as indistinct borders, or histological artifacts such as tissue shrinkage and distortion). Although both seemingly represent the same ontological construct, in reality they are fundamentally different, having been generated using different methodologies and rules and conventions of interpretation. Thus, it may be reasonable to consider the use of specific terminology to differentiate an MRI structure, i.e., an ROI, from the corresponding histologically defined structure. Although not a focus of the present study, we consider this an important issue in the field of neuroanatomical imaging. As imaging technology improves, the gap between the two representations, MRI and neuroanatomical, will become smaller. Nevertheless, this remains a fundamental issue that must be addressed when interpreting and validating neuroimaging results in translational neuroscience.

### Limitations and future studies

Manual and semiautomated segmentation procedures face two broad limitations: they are costly in terms of time and effort, and they may be less repeatable and reliable across multiple human operators and studies. To address the latter point, we have developed manuals to increase reliability by improving the level of detail and consistency of training for demarcating each brain ROI. This is important because even though fully automated segmentation provides optimal reliability, it does not provide the level of anatomical precision of manual and semiautomated procedures. The field of morphometric analysis currently relies on publicly available structural MRI datasets such as those of the HCP, with spatial resolution approximating gross anatomy more than histology (Glasser et al., 2013). Future studies should strive to increase scanner capabilities such as higher signal-to-noise ratio and spatial resolution; doing so will bring the level of *in vivo* MRI closer to that of histological characterization. This level has already been achieved in post-mortem settings in humans and experimental animals (e.g., Baroncini et al., 2012; Calabrese et al., 2015; Rushmore et al., 2020c). It is expected that segmentation accuracy at a finer grained level of brain morphometry will advance using multimodal MRI in conjunction with precise anatomical criteria. This will lead to segmentations that will more closely approximate a histological level of analysis for individual subjects, such as identifying and delineating component nuclei of complex brain ROIs (e.g., hypothalamic and thalamic nuclei).

We plan to expand from our current 50 brain datasets to a target of 200 anatomically curated datasets over the next 2 years. We expect that this will lead to further refinement of our subcortical segmentations. Given recent findings that brain-wide association studies are underpowered (Marek et al., 2022), the results presented in this paper should be viewed as preliminary. Similarly, comparisons with other segmentation methods will be important, but to produce robust findings, such an evaluation should be performed with considerable numbers of subjects. It is also critical that future data curation studies pay careful attention to the demographic information associated with the data being generated, as it has been shown that current popular atlases do not always generalize well (e.g., to younger, or non-western populations; e.g., Tang et al., 2010; Rao et al., 2017; Sivaswamy et al., 2019; Holla et al., 2020; Wu et al., 2021).

Although we feel confident in the anatomical accuracy of the present segmentation data, it is important to note that their use with deep learning or other automated technologies may produce results of which the accuracy cannot be predicted. Thus, it is critical that the results of deep learning technologies be evaluated carefully by the user to insure anatomical accuracy in line with the definitions provided in our manual.

Another point of caution is that the detection of details in certain structures depends on the resolution and quality of data. For instance, in this study, we were able to identify and segment the fifth ventricle as well as the disconnected portions of the posterior horn of the lateral ventricle, mainly because of the high quality and spatial resolution of the HCP data. The current definitions were developed using high resolution data and may not necessarily apply to segmentation of brain structures in MR images of lower quality or resolution. Thus, care should be taken when the method described herein is used for volumetric analysis of low-resolution data.

## Conclusion

We have created 50 precise anatomically based manual segmentations of subcortical structures in the human brain based on high resolution T1w structural MRI HCP acquisitions. These datasets are based on a detailed segmentation methodology implemented using a custom-developed, open-source software module for 3D Slicer. A unique feature of this study is the provision of detailed and practical manuals as a resource for self-teaching of neuroanatomical image segmentation of subcortical structures and as a reference for segmenting brain structures based on a precise and repeatable methodology. Our manuals, software and datasets are publicly available to the neuroscience community as open science resources. Overall, these resources will provide the basis for numerous neuroscience applications, including the development of future machine-learning based automatic segmentation algorithms featuring both high throughput and high anatomic precision.

## Data availability statement

The raw data supporting the conclusions of this article will be made available by the authors, without undue reservation.

## Ethics statement

Ethical review and approval was not required for the study on human participants in accordance with the local legislation and institutional requirements. Written informed consent for participation was not required for this study in accordance with the national legislation and the institutional requirements.

## Author contributions

NM, MK, SB, YR, MH, KS, AL, EY, and RR contributed to study design. RR, NM, and EY wrote the first draft of the manuscript. ER, BV, NP, HC, JC, PW-B, NM, EY, and RR performed data collection and analysis. All authors read, revised, and approved the submitted manuscript.

## Footnotes

1
www.slicer.org

2
https://github.com/PerkLab/NeuroSegmentation

## Appendix

The 50 datasets described in this manuscript are available for download on GitHub at the following URL: https://github.com/HOA-2/SubcorticalParcellations. Data files versioned at the time of this publication can be downloaded at: https://doi.org/10.5281/zenodo.6967315.

The repository includes the segmentation masks stored in NIfTI files, compressed using GZIP compression, and stored in the “nii” directory. The repository also contains an exemplary probabilistic atlas. Filenames correspond to the Human Connectome Project (HCP) subject numbers from the HCP1200 dataset. The segmentation masks are in the same coordinate system as the original data, so that there is a voxel to voxel correspondence between acquisition and mask.

The lookup table, which associates the label values stored in the mask files, the anatomical structure names, and suggested colors, is stored in the “lut” directory. The file “dseg.tsv” uses the BIDS (Brain Imaging Data Structure) format convention, while the “dseg.ctbl” file is a lookup table suitable for use in 3D Slicer. The label information is consistent across all subjects and files. The labels include the left and right structures for each of following: the amygdala, caudate, globus pallidus, hippocampal formation, lateral ventricles, inferior horn of the lateral ventricles, nucleus accumbens, putamen, thalamus, and ventral diencephalon. Labels are also included for unpaired structures: the brainstem, optic chiasm, third, fourth, and fifth (terminal) ventricles, and cerebrospinal fluid.

In addition, native MRB format data files for 3D Slicer and the Slicer NeuroSegmentation module are available upon request. They contain the auxiliary information required by the NeuroSegmentation module to guide the segmentation process as described in this paper. These files include original HCP data files and are thus subject to HCP access and redistribution agreements. As a result, we cannot freely distribute them without proof of acceptance of HCP agreements.

3D Slicer (https://www.slicer.org/) is available for download at https://download.slicer.org. The NeuroSegmentation module is available through the Slicer extension manager built into Slicer.

3D Slicer and the data described in this paper are subject to the 3D Slicer License Part B, an open-source license that permits commercial use, but provides no warranty nor liability for clinical use. 3D Slicer and these datasets are not approved for clinical use.
