# Supplementary material for: Anatomically curated segmentation of human subcortical structures in high resolution magnetic resonance imaging: An open science approach
Source: Front Neuroanat. 2022 Sep 30;16:894606. doi: 10.3389/fnana.2022.894606 (PMC9562126; doi:10.3389/fnana.2022.894606)
Supplement: Supplementary file 1 [file Data_Sheet_1.pdf]

Center for Morphometric Analysis
Massachusetts General Hospital
Harvard Medical School
cma.mgh.harvard.edu

Harvard-Oxford Atlas 2.0 (HOA2.0) Manual for Segmentation of Subcortical Brain Structures using 3D Slicer

J. Rushmore
H. Carrington
J. Chen
N. Prunier
E. Rizzoni
B. Vessey
P. Wilson-Braun
E. Yeterian*
N. Makris*

*equal contributions

Introduction

The purpose of this manual is to present procedures for reliable and accurate neuroanatomical segmentation of imaging data. These procedures use the 3D Slicer software platform, in which a specific segmentation module has been developed. This module is based on principles set forth by the founders of MRI-based volumetric morphometry or volumetrics (Caviness. et al., 1999). Volumetric morphometry began in 1987 at the Center of Morphometric Analysis (CMA) Massachusetts General Hospital (MGH) and later was used to validate FreeSurfer automated volumetric methodology (Fischl et al., 2002, 2004). The original CMA approach to MRI-based volumetric analysis used a custom-designed software platform called Cardviews. The tools and procedures developed for CardViews, which incorporated semi-automated and manual editing, have been adapted to the 3D Slicer environment as a specific neurosegmentation module. This module is designed to be used in conjunction with procedures described in this manual to perform semi-automated and manual editing of subcortical brains structures.

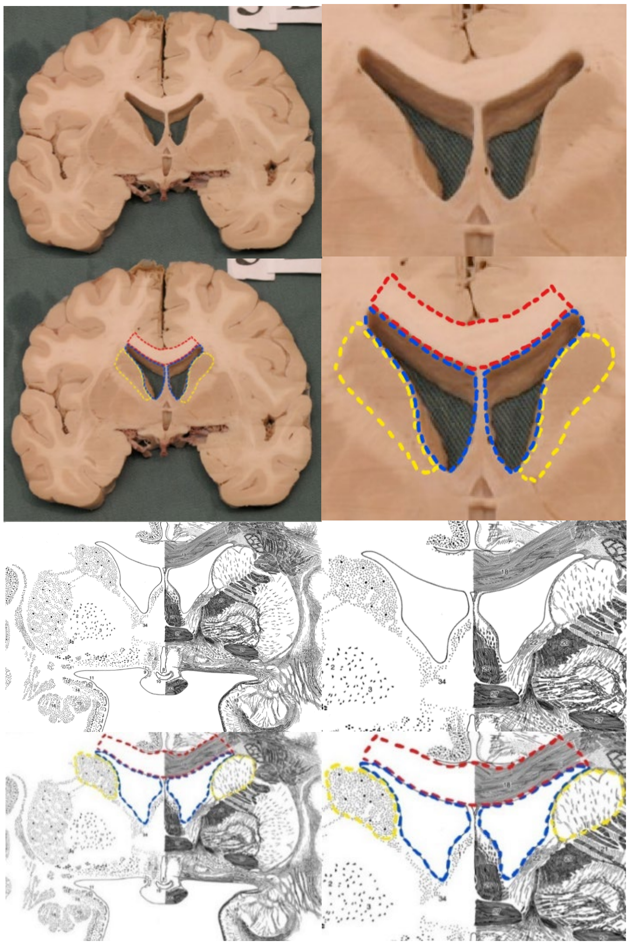

Figure 1: The caudate nuclei (yellow), lateral ventricles (blue) and corpus callosum (red) in unlabeled (first row) and labeled (second row) gross anatomical slabs, and in unlabeled (third row) and labeled (fourth row) atlas drawings (Nieuwenhuys et al., 2008).

Neuroanatomical and computational principles of MRI-based volumetric segmentation

The term *segmentation* has different meanings in *neuroanatomy* and *MRI-based computational processing*. ***Segmentation*** in general is the delineation of a group of elements (such as cells or voxels) that constitute a region of interest (ROI) and the assignment of an identifying label to that region. In neuroanatomy, segmentation involves the delineation and identification of directly visualized brain regions, which are labelled using structural, biological criteria. By contrast, in MRI analysis, the delineation and identification of brain structures are performed on computer-generated images using imaging-related criteria. The end goal of MRI-based segmentation is to parcellate images into identified groupings of voxels corresponding to neuroanatomical structures.

In ***neuroanatomy***, segmentation is the process of delineating and identifying specific neuroanatomical structures, such as nuclei, cortical regions, or fiber tracts. In neuroanatomy, a structure is delineated by outlining its exterior border by naked eye or under the microscope. For instance, in the gross anatomical coronal section in Figure 1, the caudate nucleus, a gray matter structure, is outlined in yellow. The corpus callosum, a white matter structure is outlined in red, and the lateral ventricle, a cavity filled with cerebrospinal fluid (CSF) is outlined in blue. Labeling is based on strictly neuroanatomical criteria.

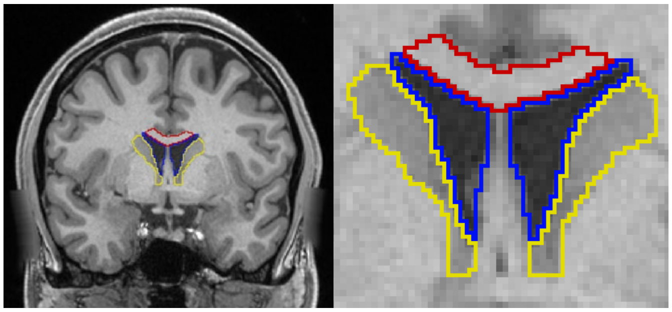

Figure 2: The appearance of the caudate nuclei (yellow), lateral ventricles (blue) and corpus callosum (red) in a T1-weighted MRI scan.

In ***MRI-based computational processing***, segmentation is the process of *classifying* voxels in the domain of magnetic resonance imaging (MRI). Labeling is based strictly on imaging-related criteria.

Classification involves grouping pixels (or voxels) with similar characteristics into different classes, based on the measurement or estimation of different features (Duda and Hart, 1973). In T1 weighted MRI a principal biophysical characteristic used to classify voxels is intensity. The classic intensity values range from 0 to 255 (Gerber and Peterson, 2008)

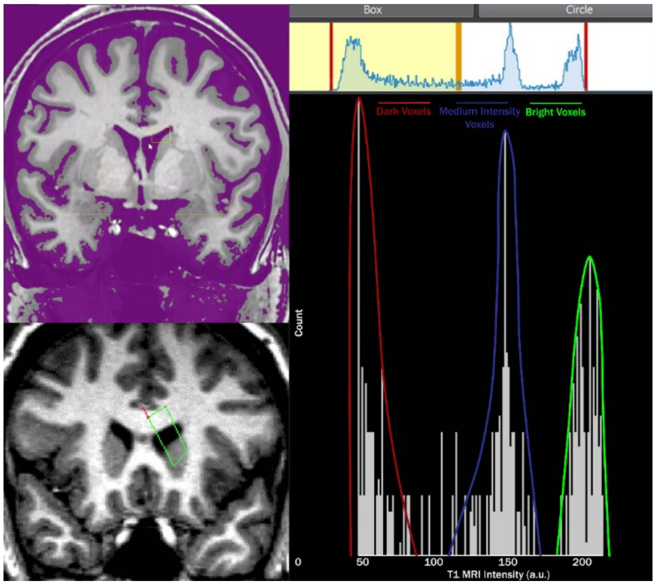

Figure 3: Brain images (left) in which rectangles were used to measure voxel intensities and generate corresponding histograms (right), the peaks of which correspond to representations of different tissue classes.

In Figure 2 we show a coronal section of a T1-weighted MRI dataset in which specific ROIs have been delineated based on intensity. The ROIs depicted in yellow represents the caudate nuclei, the ROIs in blue represent the lateral, and the ROI in red represents the corpus callosum. These ROIs are delineated through a process of classification and labeling as follows.

A group of contiguous voxels that have been classified based on intensity are typically referred to using a neuroanatomical term. However, this term is not the same as the actual neuroanatomy. In reality, an MRI-based region of interest is a correlate of a neuroanatomical structure that has been detected and sampled by a computational process. It is therefore a representation of a neuroanatomical structure rather than that structure *per se* (Caviness Jr et al., 1989; Filipek et al., 1991, 1989; Kennedy et al., 1989; Kennedy and Nelson, 1987; Worth et al., 1998).

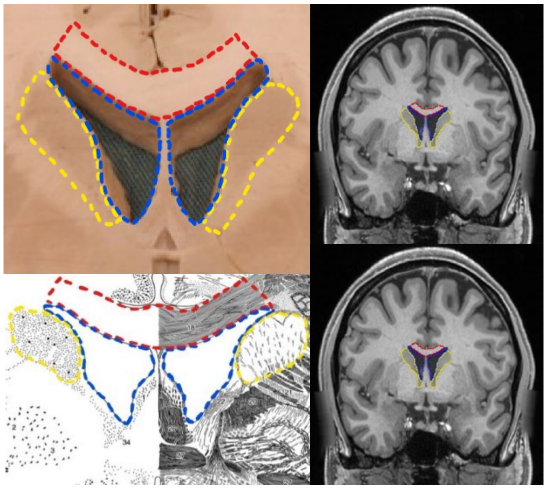

Figure 4: Correspondences of anatomical (left) and neuroimaging (right) structures.

In Figure 3, voxel intensities were collected within the green rectangle (lower brain image) to produce a corresponding histogram with three main peaks. The peak with the dark voxels (red) represents the cerebrospinal fluid (CSF), the peak with the bright voxels (green) represents the white matter, and the one with the medium intensity voxels (blue) represents the gray matter. Neuroanatomically, the CSF is primarily water molecules with inorganic ions, vitamins, and other organic molecules (Spector et al., 2015); in neuroimaging it is represented as a distribution of dark voxels. The white matter in comprises axons, myelin, supporting cells such as oligodendrocytes, and blood vessels; it is represented in neuroimaging as a distribution of bright voxels. Finally, the gray matter comprises neuronal cell bodies, neuropil (dendrites and unmyelinated axons), glial cells (astrocytes, microglial cells, and oligodendrocytes), synapses, and capillaries; this neuroanatomical tissue is represented in neuroimaging as a distribution of medium intensity voxels. The intensity distributions allow for separation of tissue classes using semiautomated techniques in accordance with neuroanatomical definitions to specify accurate and reliable ROI borders in MRI-based segmentation.

Validity in MRI-based segmentation

A morphometric analysis method is valid when it fulfills two requirements, namely, anatomical accuracy and reliability. Anatomical accuracy depends on clear definitions of brain structures, which are explicitly specified in the segmentation process as a set of detailed protocols (see subsequent sections). Reliability refers to the consistency of labeling of two or more raters (inter-rater reliability), and/or of a single rater over multiple observations (intra-rater reliability).

As an illustration of reliability, consider the following. Two observers might specify boundaries in such a way that the same number, but a substantially different set of voxels would be assigned to an ROI. A comparison of the number of voxels (i.e., volume) assigned to a given ROI may thus give a deceptive impression of the level of interobserver agreement in defining the boundaries of that ROI. Although the number of voxels may be the same, the spatial distribution of those voxels may be different. Such discrepancies may be evaluated using a percent commonality in voxel assignment (PCV) measured either in relation to two observers (inter-rater), or in the same

observer at two separate time points (intra-rater). The PCV is the percent of the common voxels assigned to an ROI by the raters, calculated in terms of the mean ROI volume (Caviness et al., 1996). This metric is similar to the Dice similarity metric. An additional measure of reliability is the intraclass correlation coefficient of absolute agreement (ICC) (McGraw and Wong, 1996) (Shrout and Fleiss, 1979), determined to be acceptable if exceeding 0.85 for the volumes assigned to an ROI by two observers or by one observed at two different time points (Makris et al., 2005).

In the remainder of this document, the procedures for specifying anatomically accurate and reliable borders of subcortical segmentations using manual semi-automated techniques are presented.

General Subcortical Brain Segmentation using 3D Slicer

Brain segmentation is a process by which the different parts of an MRI image of a brain are separated from each other using outlines. The resulting geometric metrics are then used to calculate statistically useful measures such as volume, thickness, or surface area. This procedure is typically performed for a number of brains belonging to several experimental groups and can be used to test the hypothesis that specific brain structures from two populations of subjects are different. Segmentation requires knowledge of neuroanatomy, but much of this knowledge can be acquired during the segmentation process. The goal of this manual and method is to provide a means by which efficient morphometric analysis can be performed.

Basics of segmentation

3D Slicer is a visualization and analysis program for neuroimaging. 3D Slicer loads a brain volume and visualizes 2D sections of the brain in the coronal, axial, and sagittal planes. This program is also used to identify regions of the brain and label those areas. This guide presumes that 3D Slicer is installed on the computer (https://www.slicer.org).

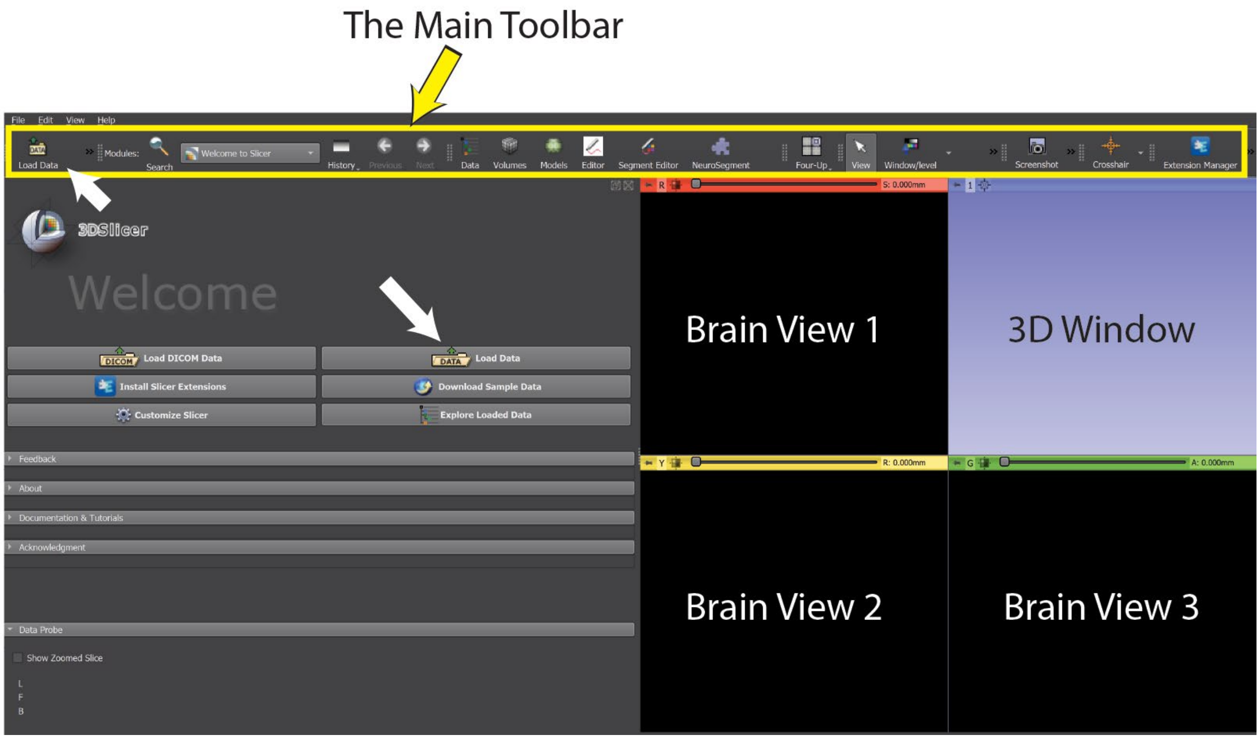

When 3D Slicer is first opened, a screen similar to the one displayed above will appear. The screen contains three main regions. The main toolbar (yellow arrow above) is a row of buttons, dropdown menus and commands. This is the location where common operations are accessed, such as the command to load data (white arrow). On the welcome screen (figure above), the Load Data button can also be found on the left-hand side of the main screen (lower white arrow). On the right-hand part of the screen, four windows are present. The window with the blue background will show a 3D rendering of the segmented brain regions, and the three windows in black will each display a

cardinal view of the MRI image - one view is in the axial plane, one is in the sagittal plane, and one is in the coronal plane. Segmenting is performed in the 2D windows to generate a 3D object.

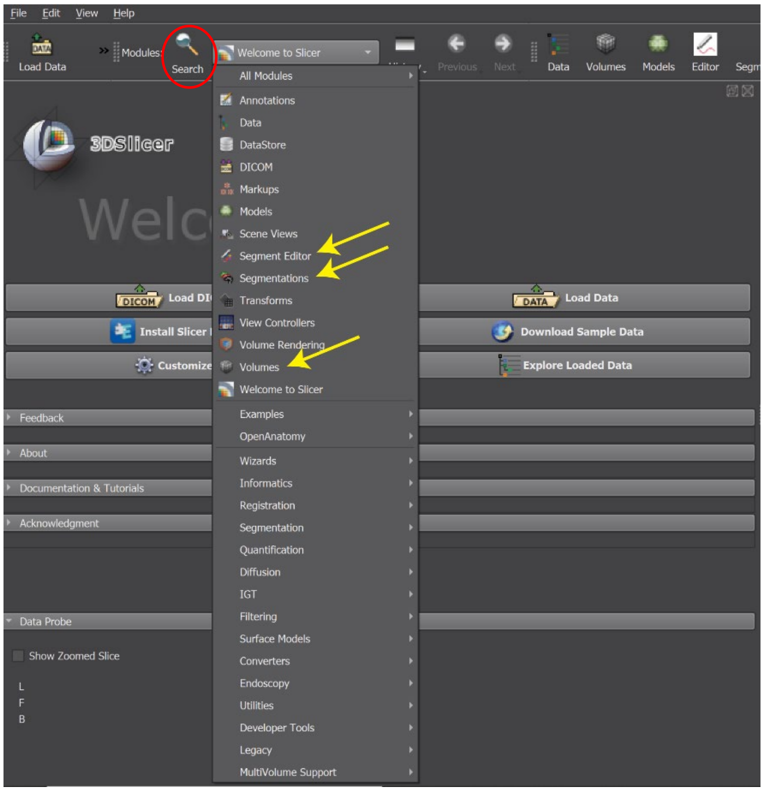

At its core, 3D Slicer is a visualization tool linked to a large number of modules. Modules are constantly being improved and added and contain specific functionalities. For segmentation, there are three main modules that will be used: Segment Editor, Segmentations, and Volumes. Access these using the drop-down menu in the Main Toolbar. The three main modules are indicated by yellow arrows in the image to the right and may also be located by entering their names in the Search button to the left of the drop-down menu. The **Segment Editor Module** contains tools to name and outline segments (regions of interest, ROIs) of the brain. The **Segmentations Module** contains commands to adjust the appearance of the segments (e.g., make them visible or invisible; change their opacity or border line width). The **Volumes Module** contains information about the MRI volume, including the number of slices, resolution, and brightness and contrast settings.

The NeuroSegmentation module contains many of the functions from these three modules. To select the NeuroSegmentatioin module, click the Search icon next to the dropdown menu (red circle), type NeuroSegmentation, and select the module that appears in the list.

1. Loading files

To begin, load an MRI dataset. This will be a T1-weighted MRI volume. Images acquired with a T1 weight accentuate certain tissue properties and are conventionally used for segmentation. After clicking the Load Data button a window will be opened. At the top of the window, identify and depress the Choose File to Add button, and in the subsequent window, navigate through the folders on the computer until the folder with the T1-weighted MRI file is located. This guide presumes that preprocessing has been performed (intensity correction, and spatial normalization). Be sure to clarify whether these operations have been performed prior to proceeding. Select the file and depress the OK button at the lower left of the subsequent window. If four visualization windows are not observed, click on the button next to the arrow button in the Main toolbar and select Four-Up.

After a brief loading window, three images should appear, one for each orthogonal viewing plane of the brain. The red window typically contains images in the axial plane (A in figure to right), the yellow window contains images in the sagittal plane (S in figure to right), and the green window shows images in the coronal plane (C in figure to right). Use the horizontal slide bars above each window to cycle through the images. The three windows each contain different views of the same volume, and it is often of utility to view a structure using all three views at once. To facilitate this, depress the Crosshair button, which is located on the Main toolbar on the far right. Position the mouse over any image and depress and hold the Shift key. Moving the mouse will reposition the crosshairs on the image such that the center of the crosshair is at the mouse point. The center of the crosshair identifies a single point in the brain volume visualized in the different orientations in the three windows.

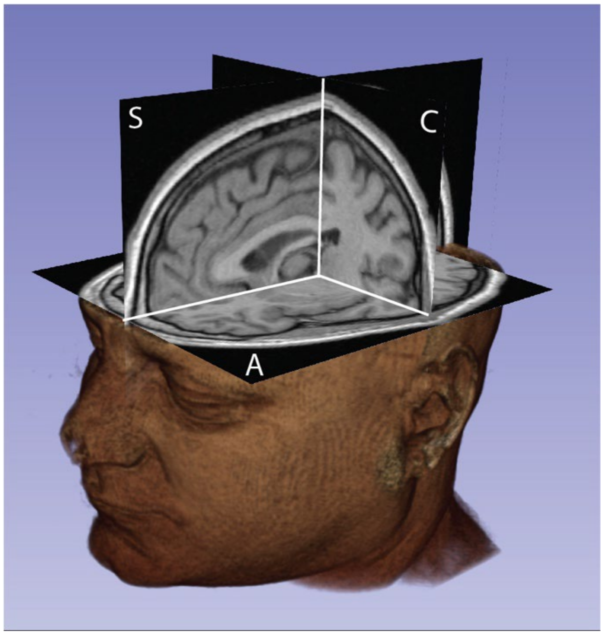

Go to the Segment Editor module in the module selector dropdown menu in the Main Toolbar. Notice that the entire left part of the window will now be filled with new buttons and drop-down menus. Identify a drop-down menu next to the Segmentation button. Select ‘Create New Segmentation As’ in this drop-down menu. In the window that then opens, name the segmentation file. It is useful to identify the case and the operator (i.e. the person performing the segmentation) in the segmentation name. For example, for the subcortical segmentation of Case 015, name the segmentation Case015_SubcorticalSegmentation_XX, where XX are the initials of the operator. For the master volume drop-down menu underneath the ‘Segmentation:’ menu, select the T1 image.

2. Segment Names

Under the Segmentation and Master Volume drop-down menus, depress the large Add button. In the table underneath, a name will appear, called Segment 1. To the left of the name is an eye icon, which toggles the visibility of the segment. Next to the eye icon is the color and the opacity. Double-click on the name to rename the segment in the text box. Clicking on the color box will produce a second window. Here the segment name can be selected and changed, the color can be altered, and the segment may be selected based on a list of preexisting structures.

This list of structures should be pre-loaded. In the case it is not, click on the left-hand arrowhead, an operation which will expand the window. Above the list and at the top of the window, notice a dropdown menu. Click on the menu and select the relevant list for the study. For subcortical segmentation, the dropdown is named ‘General Segmentation category and type.’ Make sure the correct list is present before beginning the study.

A Note About Radiological Imaging. Brain orientation is standardized according to a radiological convention; namely, the brain is viewed from the perspective of standing at the foot of the bed of a patient or research subject. Viewed this way, the ***left*** side of the patient is on the ***right*** of the screen. This is counterintuitive. A good reminder (initially, at least) is to attach ‘Left’ and ‘Right’ written labels on the monitor.

Segmentation

In this part of the manual, the tools of segmentation will be introduced. When described, segmentation tools will be referenced by bold and italicized text (e.g., ***Draw*** tool). They will be applied to the lateral ventricle as an example of how to segment a structural brain region of interest (ROI). Each brain structure will be segmented according to intensity values and neuroanatomical definitions. Neuroanatomical definitions are provided later in this manual. The means by which these definitions may be operationalized in the segmentation of MRI images are detailed in the explanations of specific segmentation tools below.

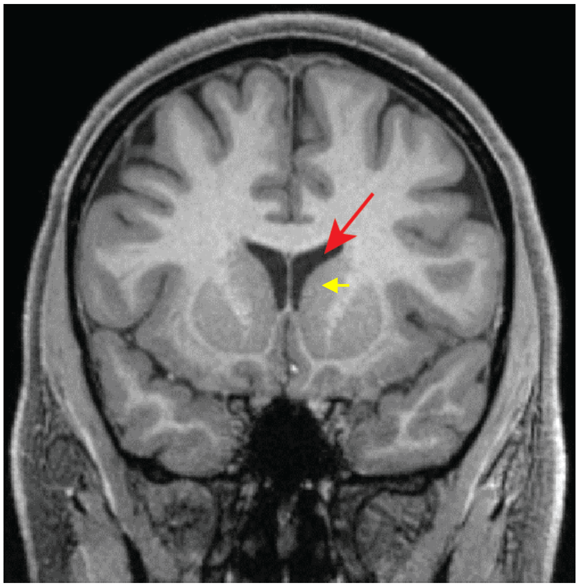

In the T1-weighted MRI image to the right, the lateral ventricle appears as a roughly triangle-shaped black space at the tip of the red arrow. The lateral ventricle contains cerebrospinal fluid (CSF), which appears dark on T1-weighted MRI images. The lateral ventricles are paired, and there is a left and right lateral ventricle, one in each brain hemisphere. Move the horizontal slide bar above the green (coronal) window until an image like the one depicted to the right is seen. The up/down arrow keys or the mouse trackwheel may also be used. In this particular MRI slice, the upper border of each ventricle is the white matter of the corpus callosum that is composed mainly of axons. The lateral border of each ventricle at this level is a gray matter structure called the caudate nucleus (yellow arrow) that bulges into the ventricle and appears gray.

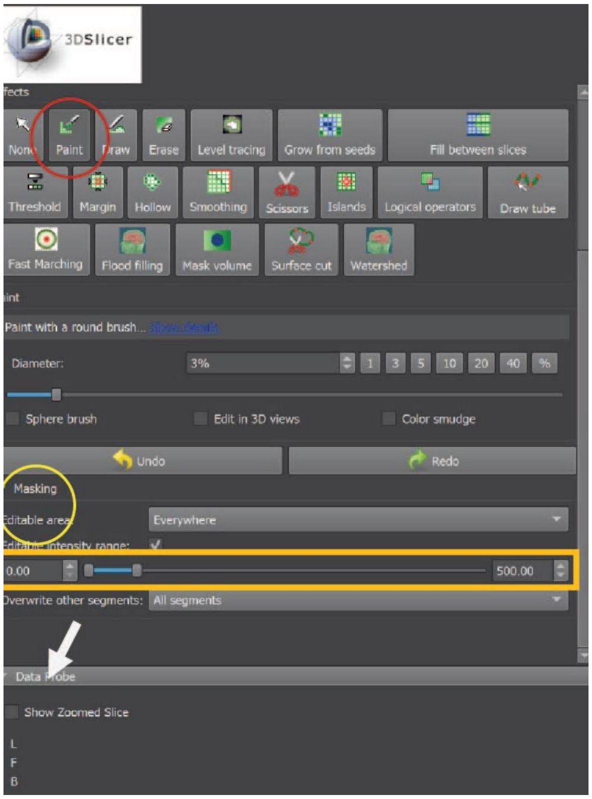

A fast and accurate way to segment each ventricle or any structure with defined borders is to use the ***Paint*** tool (left image, red circle) or the ***Draw*** tool (to the right of the ***Paint*** tool). By themselves, each of these tools will generate an overlay on top of the brain image. Select the ***Paint*** tool, adjust the diameter halfway down the screen to 10%, and click and drag on top of a brain image. Notice that this action produces labeling of the selected region of the image. The same type of labeling can be performed with the ***Draw*** tool. For this tool, use a series of left clicks to enclose a space if using a mouse, or just draw with the pen. Once the region is enclosed by the line, press Enter or right-click with the mouse to finalize the labeling. The ***Erase*** tool (the button to the right of the ***Draw*** tool) will eliminate previously drawn label. The ***Paint*** and ***Draw*** tool are useful, but more powerful in

conjunction with the editable intensity range option located under the masking sub-tab (yellow circle). Masking will allow the selection of voxels that fall within a certain range of specified intensities (orange rectangle). In the image illustrated above, drawing using masking will only label voxels with intensities between 0.00 and 500.00.

Understanding the neuroanatomical definition of a brain ROI is critical to specify any structural border. To label voxels that should be included in the ventricle and exclude voxels that are part of surrounding white or grey matter, an intensity range needs to be specified in advance. One way to do this is to select the pointer tab (labeled as ‘None’ to the left of the paint button) and point the cursor at different voxels in the image. The voxels that correspond to the ventricle on a T1 image should have low values, and the corresponding intensity values are observed in the lower left-hand part of the window, under Data Probe (white arrow; the number will appear on the far left of B). By sampling the voxels in the ventricle and those in the surrounding structures, the intensities to include in the range to mask the ventricle are obtained. Pick a value that is a good estimate of the border of the lateral ventricle. Set the lower editable intensity range to 0. Set the upper value as the estimate that was just made. Label one of the ventricles using the ***Paint*** or ***Draw*** tool with this masking range. Note that only the voxels in the specified range should be selected, and those above the range should be unlabeled. Label the gray and white matter, and then zoom out to evaluate the results. Try a few different intensities as the upper limit of the mask and view the results. Note that this approach is time consuming, imperfect, and arbitrary. In addition, changing the brightness of the image will change the perceived border, and the above method of determining the border would yield inconsistent results.

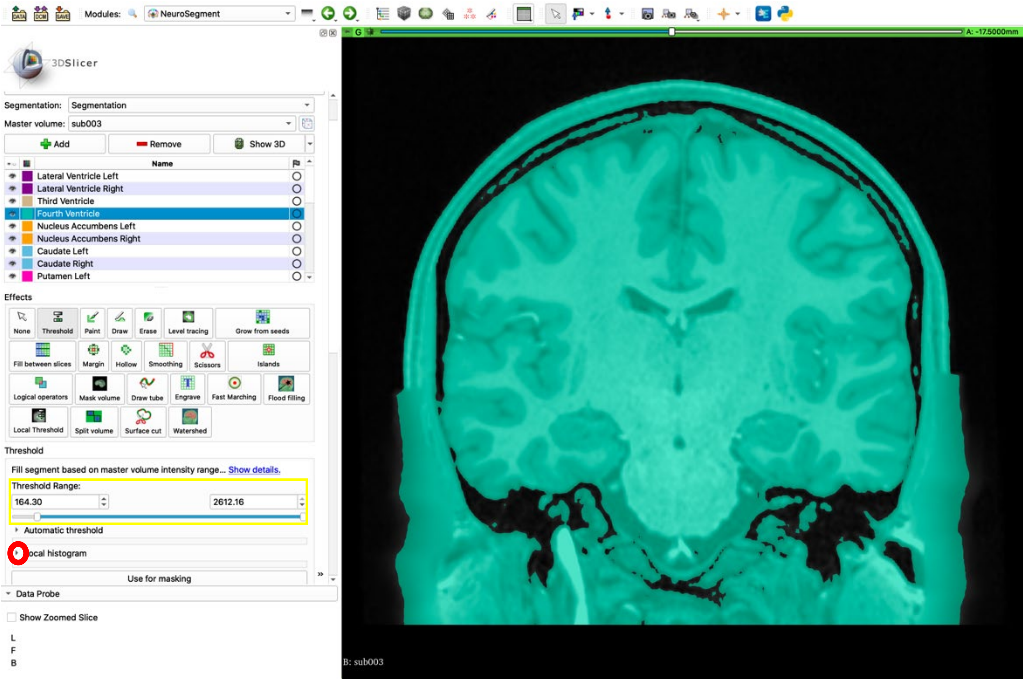

A more precise method has been developed to eliminate the issues identified above. Before illustrating this approach, click on the Erase button, and click and drag over labeled voxels to eliminate them. Click the button labeled ***Threshold*** and observe that the whole image flickers. More accurately, an image overlay will cyclically become more and less transparent. This overlay is tied to the intensity values that appear in a bar under Threshold Range (yellow box in figure above).

This bar is adjustable, and the upper and lower bounds of the editable intensity range can be varied and seen in real time. This approach is more reliable and accurate than that described above because a threshold can be generated and the results viewed for the whole image at once, rather than determining the range by looking at representative voxels. Once a threshold value is generated, click on the Use for Masking button, and the range is automatically imported into the masking section.

This tool will now be applied to generate the border of the lateral ventricle. With the ***Threshold*** tool on and the image flickering, click on the arrowhead to the left of 'Local histogram' (red circle in image above). This will produce a graph and several buttons.

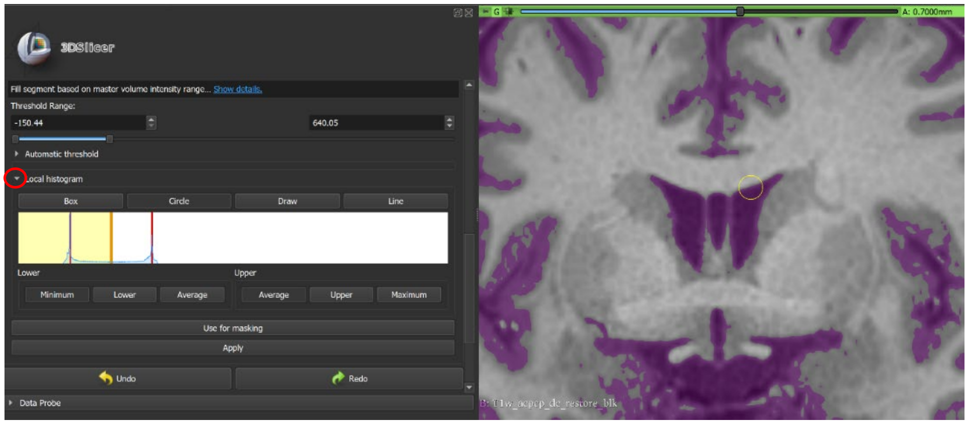

Select the coronal view and drag the slider until a section similar to the image on the right is seen. Select the Circle button under local histogram, click on the border of the white matter and the ventricle and drag. Drag the mouse until the circle is of the size seen on the image above. In the histogram window on the left, a histogram with two peaks will then be generated. The left peak represents the voxels associated with the lateral ventricle and the right peak represents the voxels associated with the white matter. Change the Lower bound button under the window to "Minimum" and the Upper bound button to "Average." Three vertical lines will be observed. Click and hold on the apex of the first (left-hand) peak and drag the mouse to the second (right-hand) peak. The orange line is now situated in the middle of these two peaks and represents the threshold between the white and dark voxels. Click the "Use for masking" button. This button will now apply this threshold range to the segmenting tools.

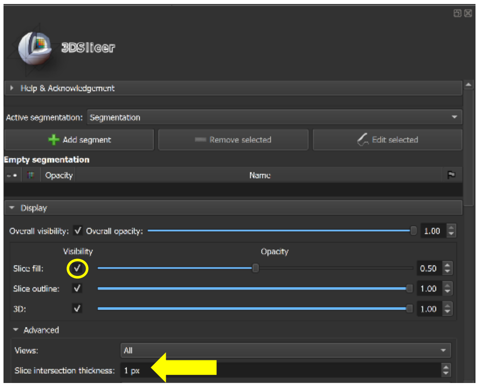

At this point, it is helpful to change some of the viewing settings of the segments. Go to the **Segmentations module**. Under display, <u>uncheck</u> Slice Fill (yellow circle). Then click the arrowhead to the left of 'Advanced' (located immediately below the slide bars) and increase the Slice Intersection Thickness (yellow arrow) to 3 px. The resulting labeled segments will not be filled by a colored overlay but instead indicated by a line. These settings will allow visualization of the voxels within a segmentation rather than obscuring the data with an opaque or semi opaque overlay.

Now that the tools have been introduced, it is time to segment the lateral ventricle.

The Lateral Ventricle

The shape of the lateral ventricle changes from anterior to posterior. In the image below, the three-dimensional morphology of the lateral ventricle is depicted in the left panel in a sagittal plane. The main parts of the lateral ventricle are labeled: the anterior horn, the body, the atrium, the posterior horn, and the inferior horn. The atrium is the region where the body, the posterior horn and the inferior horn are confluent. Each numbered panel shows a coronal image through the vertical lines in the left-hand image, and the outlined ventricle is shown in purple. In the anterior coronal section (1), the anterior, or frontal, horn of the lateral ventricle appears roughly triangular in shape. The body of the lateral ventricle is principally located in the frontal lobe of the brain and is represented in purple outline in the second coronal section (2) where it appears more ovoid. The atrium of the lateral ventricle is principally located in the parietal lobe and is depicted in the third coronal image (3) where it extends inferiorly. The posterior, or occipital horn is shown as a small space in the fourth coronal section (4). The inferior, or temporal, horn of the lateral ventricle (ILV) is identified in (2) but, by convention, is not included in the segmentation of the lateral ventricle. The ILV will be segmented as a separate ROI in a later step.

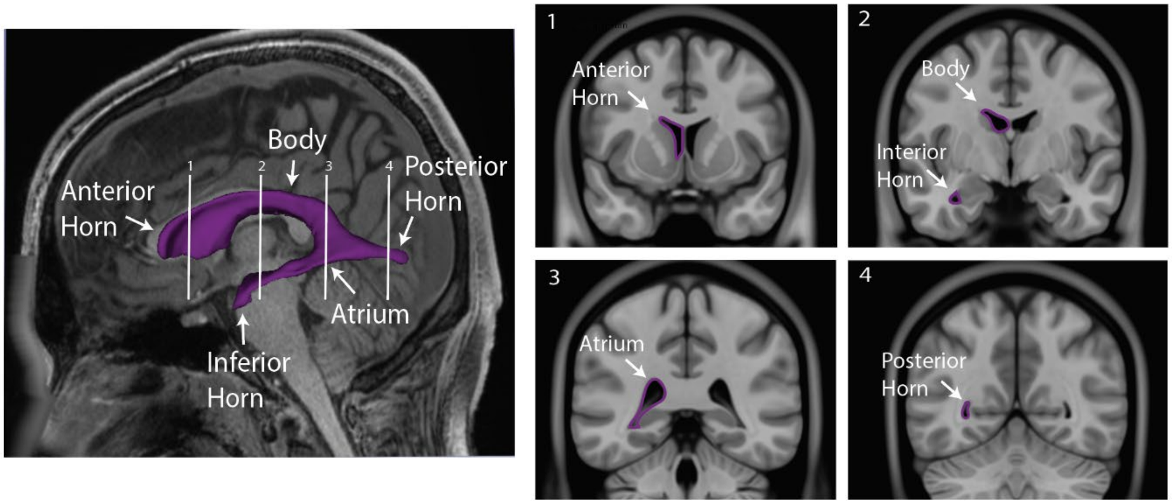

To begin, move the slider on the green window to identify an image similar to that shown on the right. Use the ***Threshold*** tool to generate the border, as discussed in the previous section, and use the ***Paint*** or ***Draw*** tool to segment the ventricle at this coronal level. Move anteriorly with the same threshold value to segment the anterior end of the ventricle. Return to the first segmented coronal slice and continue segmenting the ventricle posteriorly, taking a new threshold value for each coronal section.

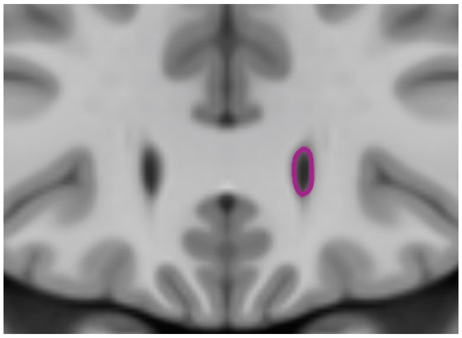

In more posterior sections, the caudate nucleus will appear to bulge into the lateral aspect of the lateral ventricle. The medial border of the lateral ventricle is a thin membrane called the septum pellucidum (between white arrowheads). When segmenting one ventricle, be sure not to extend the labeling to include the septum pellucidum. Continue to take the threshold between the white matter and the CSF to segment the lateral ventricle.

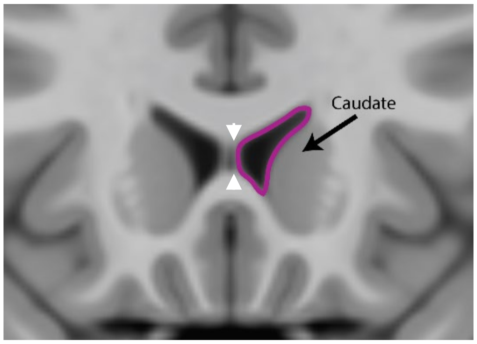

More posteriorly, the caudate nucleus will change shape and the ventricle will enlarge. Continue to take the threshold value with the white matter on the superior border of the ventricle, and segment according to the purple outline in the image to the right.

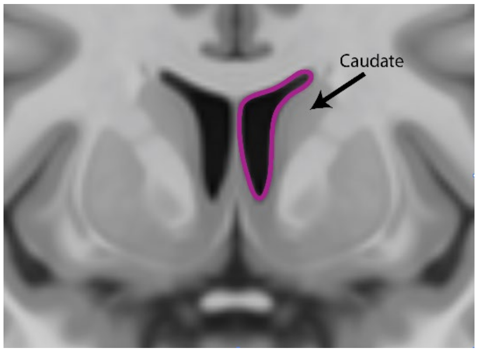

Further posterior, each lateral ventricle communicates with the third ventricle, which is located on the midline (number 3). The communication between the lateral ventricle and the third ventricle is called the foramen of Monro, and this foramen is considered to be part of the lateral ventricle segmentation. Segment the ventricle such that the outline approximates the purple line shown in the image.

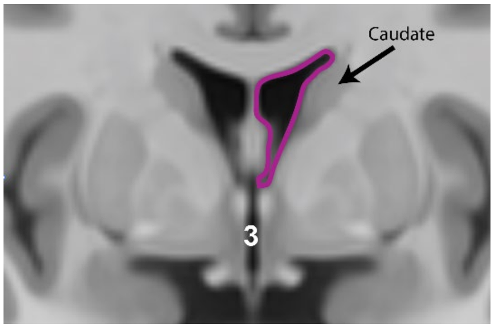

Posterior to the foramen of Monro, a small space will intervene between the third ventricle (3) and the lateral ventricle. This space is called the *transverse cerebral fissure* (orange). It contains cerebrospinal fluid (CSF), but is not a ventricular space. Do not segment this space as part of the lateral ventricle. Continue to segment the border of the lateral ventricle as shown, using the threshold value with the white matter. Notice in this section, the caudate nucleus is smaller and laterally located. In more posterior sections, the caudate will diminish in size even further.

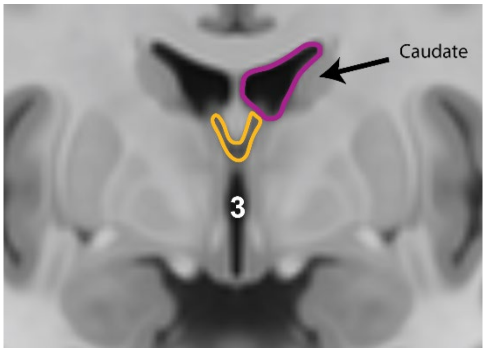

Important: using the prescribed threshold value will leave some unlabeled voxels within the ventricles. These voxels will be dealt with in a subsequent step.

In a more posterior section the inferior part of the lateral ventricle is bordered by a white matter pathway called the fornix (yellow arrowhead). The caudate nucleus has diminished in size. Segment the ventricle as shown using the threshold setting between white matter and ventricular CSF as before. Notice that the inferior horn of the lateral ventricle (double white arrowheads) is not segmented. This portion of the ventricle is given its own segment and not included in the lateral ventricle segment *per se*.

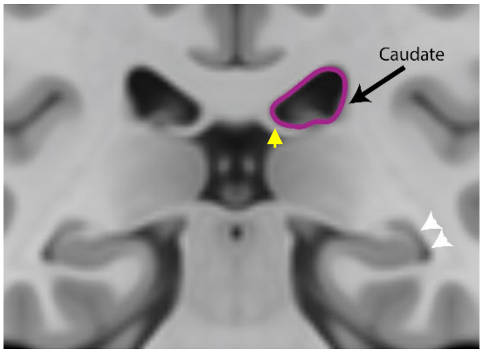

Continuing posteriorly, a thin portion of the lateral ventricle extends inferiorly. This coronal section is considered the beginning of the atrium of the lateral ventricle (see the earlier anatomical overview). The caudate nucleus forms part of the ventricular wall laterally.

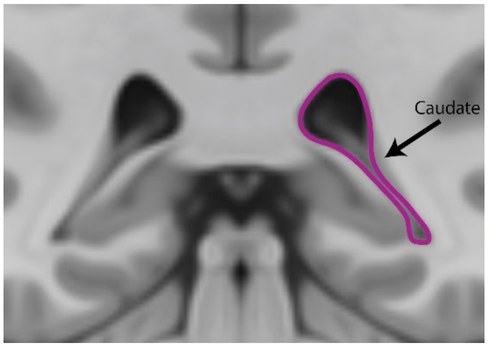

The atrium will expand in width as shown in the section to the right. Continue to threshold with the white matter as before. At this section, the use of the ***Threshold*** tool will determine a distinct border between the lateral ventricle and adjoining white matter, but will typically leave unlabeled voxels within the lateral ventricle. These voxels belong to the choroid plexus (tip of yellow arrow), the organ that secretes CSF. These voxels will be included in the lateral ventricle ROI at a subsequent step.

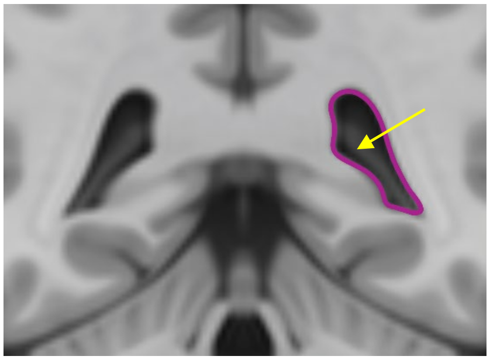

Towards the back of the brain, the atrium of the ventricle will shrink until it has the appearance of the small purple outline shown on the right side of the image (also shown at the tip of the red arrow on the left side of image, and in the 3D image). This is the posterior horn of the lateral ventricle. In many brains, the posterior horn appears to end in the anterior occipital lobe, only to reappear in more posterior sections. In anatomical dissections, these two compartments are continuous and connected by a collapsed space. This collapsed space is not discernable using current MRI technology. To be sure this posterior compartment is segmented, continue scrolling through more posterior coronal sections.

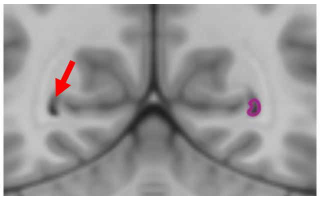

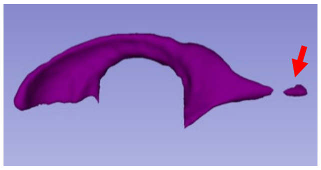

As indicated previously, the segmentation of the lateral ventricle will contain unlabeled voxels within. These voxels belong to intense portions of the choroid plexus, the vascular structures that secrete the cerebrospinal fluid inside the ventricles. By convention, the choroid plexus is included within the lateral ventricle segmentation and therefore these voxels should be labeled. To segment these pieces of the choroid plexus in the ventricle, use the ***Draw*** tool or ***Paint*** tool, but turn off the threshold mask (i.e., uncheck the '***Editable intensity range***').

Start in the most posterior section that shows the choroid plexus (yellow, see image on right). Segment any residual voxels remaining within the lateral ventricle segment so the choroid plexus is included. The brightness and contrast of the images in this section have been increased to better visualize the choroid plexus.

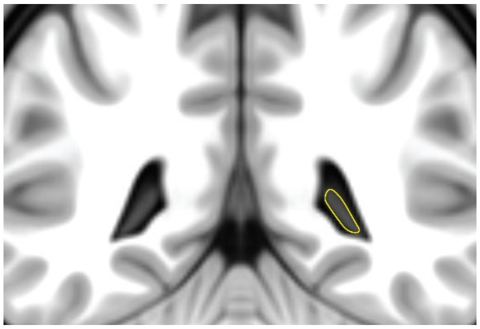

In more anterior sections, the choroid plexus will change shape and will typically fill the inferior extension that characterizes the anterior-most part of the ventricular atrium. Segment the lateral ventricle so that it includes the choroid plexus (yellow outline and arrowheads) within the ventricular borders. It may be of utility to turn off the segmentation overlay to view the border of this extension more precisely – use the '***g***' key to toggle the segment visualization.

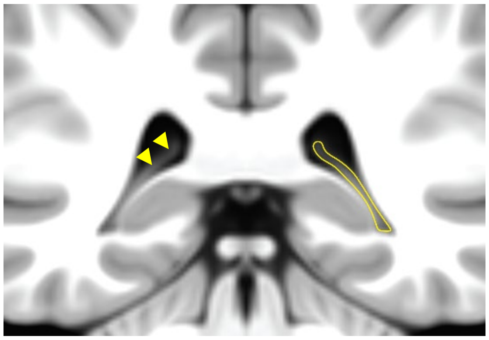

Slightly more anteriorly, the lateral wall of the lateral ventricle will become the gray matter of the caudate nucleus (dotted line). Be sure to identify the curved border of the ventricular margin with this region of the caudate nucleus and to not include the caudate as choroid plexus. This gray matter border will be verified in a subsequent step below.

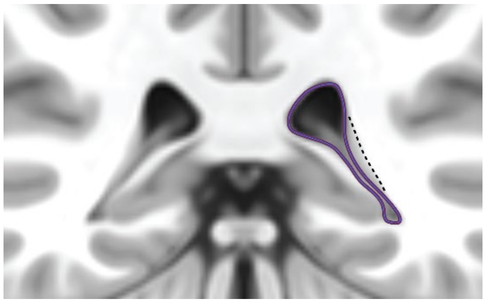

At the coronal level of the body of the lateral ventricle, the choroid plexus is located adjacent to the fornix (red arrowhead). Include unlabeled voxels as the lateral ventricle segment if they are in the outlined yellow location that corresponds to the choroid plexus.

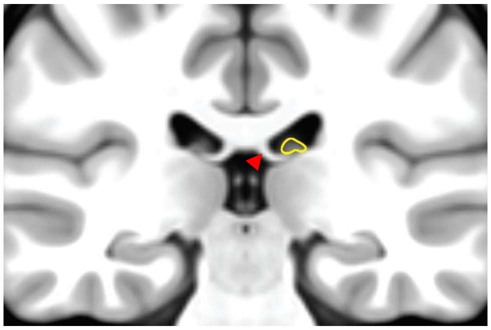

Keep in mind that the ventricular segmentation that includes the choroid plexus should not extend into the transverse cerebral fissure. At the level of the foramen of Monro, the space of the transverse cerebral fissure is shown as an orange oval. The outlined yellow portion represents the choroid plexus filling the foramen and adjacent to the transverse cerebral fissure. This choroid plexus should be included in the lateral ventricle segment.

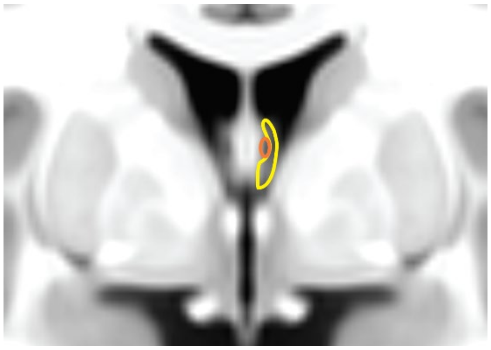

In the first pass of this segmentation, the border of the lateral ventricle was created with the white matter using the ***Threshold*** tool. This is accurate and appropriate for the CSF-white matter border of the lateral ventricle, but not for the border of the ventricle with gray matter structures (e.g., the caudate nucleus, white arrowheads in image below) that line the lateral ventricle. In the left-hand image below, the border is illustrated by a purple line. Notice that the superior border of the lateral ventricle with the white matter is accurate, but the medial, lateral and inferior borders of the ventricle with gray matter are inaccurate (see arrowheads). In these locations, the borders need to be modified to produce a correct border (yellow line, right image). Thus, the next step in this segmentation procedure is to revise the border of the lateral ventricle with gray matter structures.

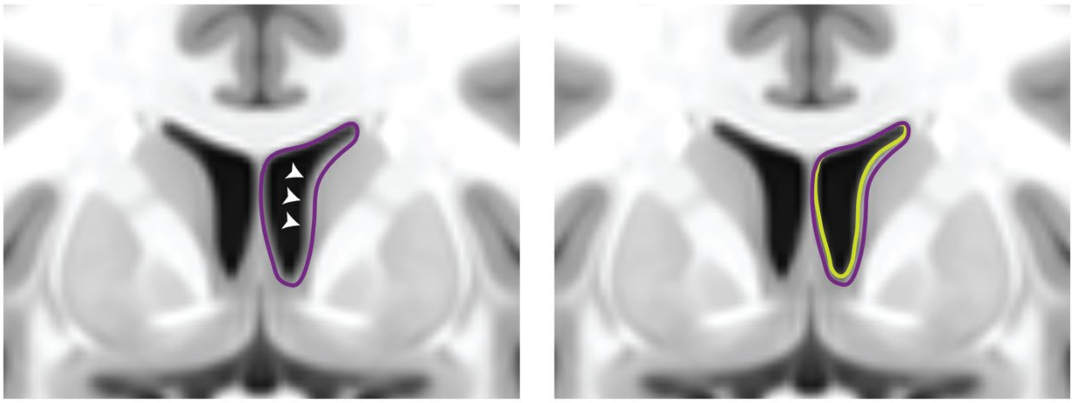

To do so, select a coronal section similar to that shown to the right. Click on the Threshold button, and select a circle that includes the CSF space of the lateral ventricle and the caudate nucleus (yellow circle). As previously, select the peaks. However, this time, change the settings and depress the Average button as the lower bound, and the Maximum button as the upper bound. Then select the '***Use for masking'*** button.

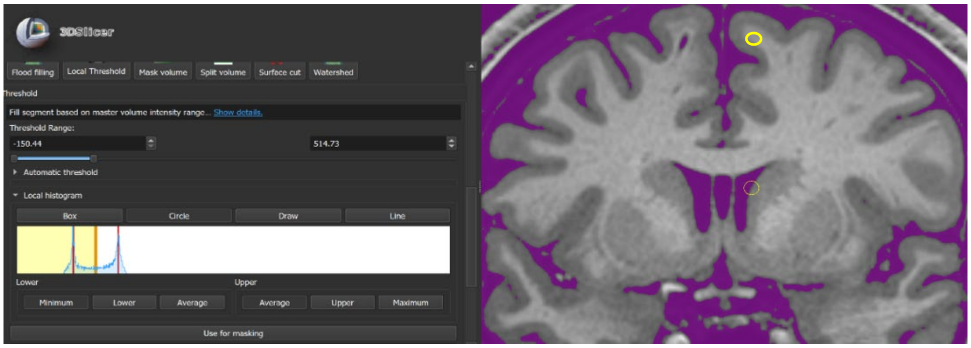

Now select the Erase button. Using an appropriately-sized circle from the ***Paint*** tool, click and drag over the border

of the gray matter structure with the ventricle. Notice that the segmented voxels that were overlying the caudate nucleus have now been erased, and that the border of the ventricle with the caudate has been revised and is now more accurate.

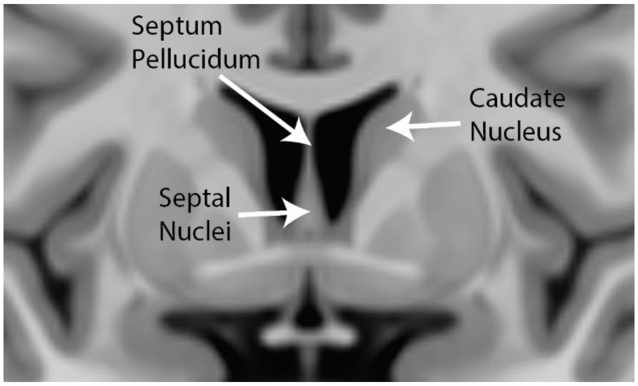

Start from the anterior portion of the lateral ventricle and move posteriorly. Use the ***Erase*** tool to refine the borders of the ventricle with the caudate and other gray matter structures (see image at right). Be sure to take a threshold measurement at each section to correctly specify the border between the gray matter and CSF space. The caudate will be the largest gray matter structure impinging on the ventricle, but the medial border of the ventricle (septum pellucidum) and the gray matter on the ventral aspect of the anterior part of the ventricle (e.g., septal nuclei) will also need to be revised according to the above procedure. Continue posteriorly until the caudate nucleus ends.

Review the segmentation on axial and sagittal views, and then on the 3D view. For the latter viewpoint, go to the Segment Editor module window and click on “Show 3D” above the label names. This will reveal a 3D representation of the lateral ventricle. In the 3D window, click and drag inside the window to rotate and inspect all aspects of the lateral ventricle (see image below).

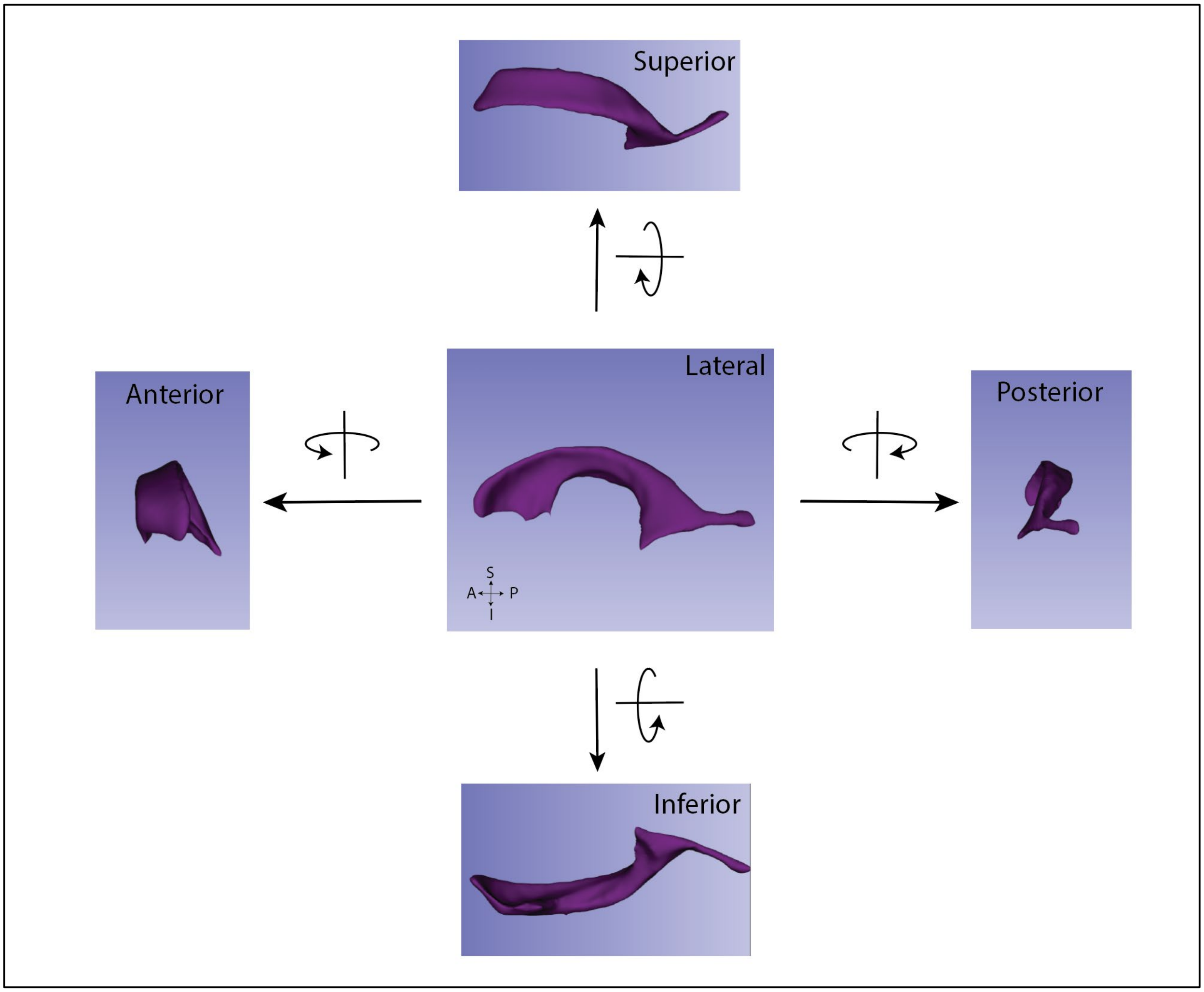

This is a good way to examine whether there are any inconsistencies or missing voxels. Inclusion of the choroid plexus in the lateral ventricle should produce a relatively smooth inferior surface of the lateral ventricle in 3D. If there is an irregular surface, use the ***Crosshair*** tool in the menu bar. Select 'Basic Crosshair.' In Four-panel window view, press Shift and move the cursor to specify a location at the crosshair in 3D. View the corresponding location in coronal, sagittal or axial slices to verify the segmentation.

Once the lateral ventricle segmentation is complete, be sure to save the segmentation file. The ***Save*** button is on the Main Toolbar, typically third from the left. Click this button and a window will appear with several files. The scene file (with a .mrml at its end) saves all the visual preferences, and files associated with the work. Underneath the scene box are the images (e.g., T1w images). Since there are no changes to this file, it will not have a check mark next to it and will thus not be saved. Below the image file is the segmentation file (e.g., Segmentation.seg.nrrd). This file will have a check mark next to it and will therefore be saved. The names of the files may be changed by clicking on them and retyping. Specify the directory in which the files will be saved by clicking '***Change directory for selected files***.' Navigate to the folder where the files will be saved, click OK, then ***Save***.

The process of segmentation is flexible and can be changed as needed. For the current segmentation of subcortical structures, the following workflow is used.

Subcortical Segmentation Workflow

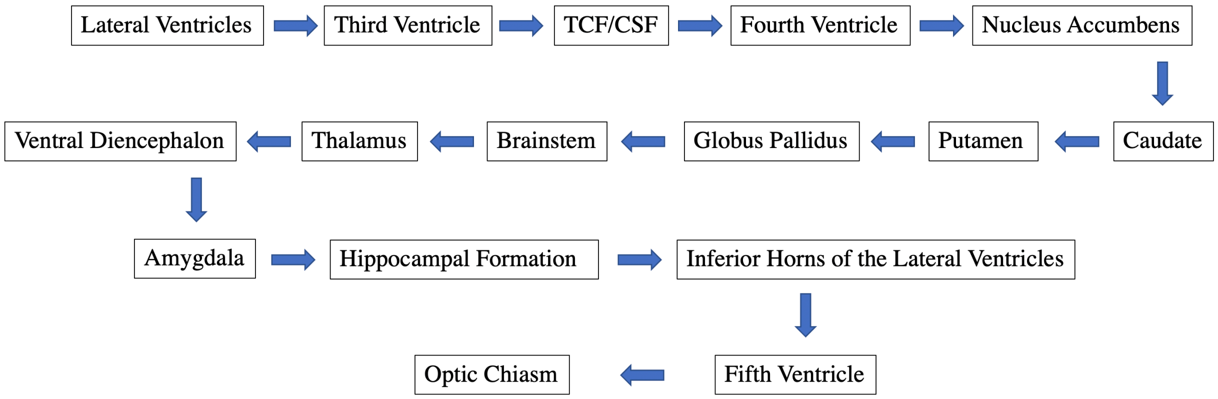

The Third Ventricle

The third ventricle is a singular CSF-filled space on the midline of the brain. It is best appreciated on a sagittal view of the brain as a dark space (left; tips of orange arrows), which has a characteristic shape when segmented (tan object, right). It is segmented in the coronal views using the ***Threshold*** tool.

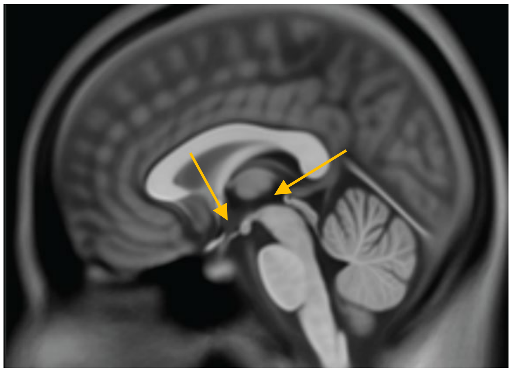

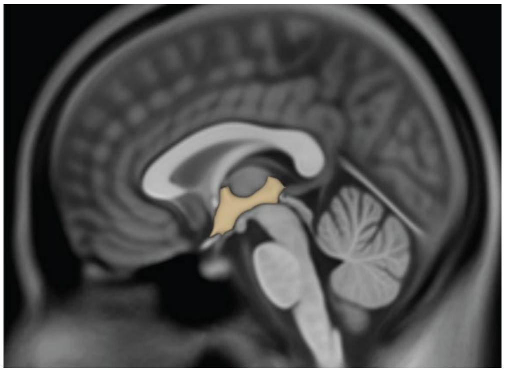

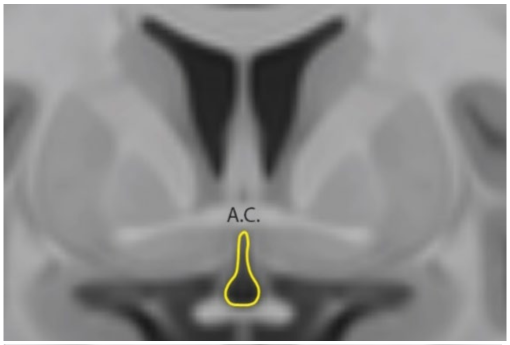

Select the third ventricle segment in Segment Editor. In the coronal view, progress to the level of the anterior commissure (A.C. in figure to left). In this coronal section, the third ventricle is shaped like a hanging tear-drop. Use the ***Threshold*** tool to generate the borders with the gray matter of the hypothalamus laterally and the white matter of the optic chiasm ventrally.

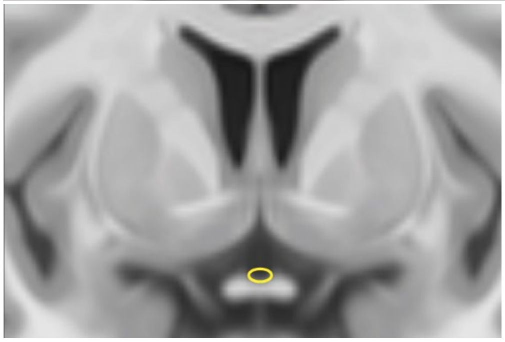

Next, proceed anteriorly. The teardrop shape will attenuate, move inferiorly, and compress until the third ventricle appears as a flattened pancake on the top of the optic chiasm (see figure to left). Given the small size of the third ventricle at this level and the thin membrane that separates it from the surrounding subarachnoid space, the ***Threshold*** tool may be of limited utility in delineating the superior border; here, adjust the contrast to find the thin membrane and segment without masking.

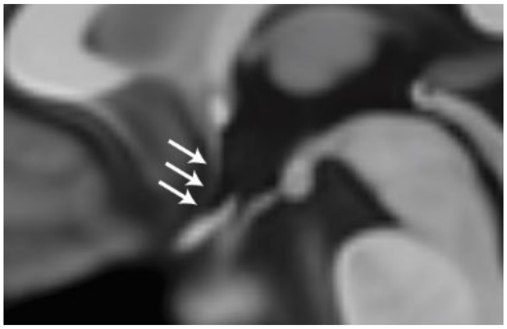

Use the ***Crosshair*** tool along the midline to view the third ventricle from a sagittal perspective. This will insure the ventricular segmentation does not extend past the anterior and superior border of the ventricle (white arrows). It may be useful to change the brightness and the contrast to visualize this border. Proceed anteriorly until the third ventricle comes to an end.

Return to the anterior commissure. Move to the next posterior coronal section. At this level, the teardrop shape changes to a tall and thin profile (yellow, below). Segment the third ventricle using the ***Threshold*** tool. In this or the next posterior segment, the lateral ventricle segmentations (purple) will extend inferiorly to form portions of the lateral and superior borders of the third ventricle. These relationships are depicted below.

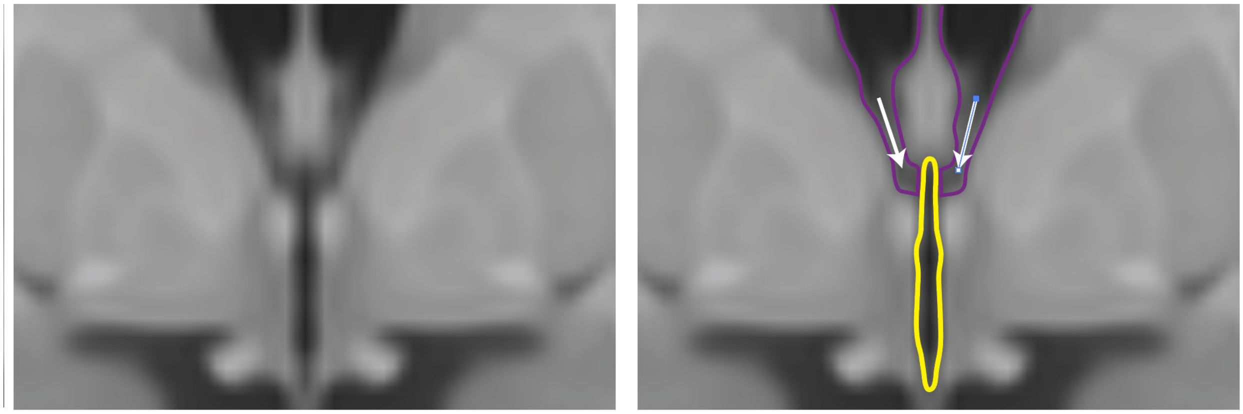

Segment the third ventricle as described above.

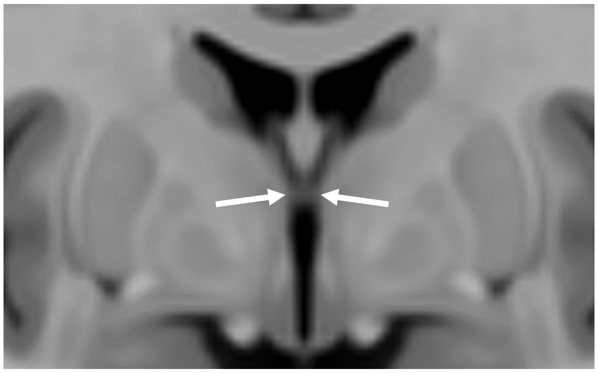

More posteriorly, a structure will appear to delimit the superior border of the third ventricle. This structure typically has the same intensity as the choroid plexus of the lateral ventricle. This is the choroid plexus of the third ventricle (tip of white arrows), and you will return to this region at the end of the third ventricular segmentation step. For now, segment the dark portion of the third ventricle as previously described.

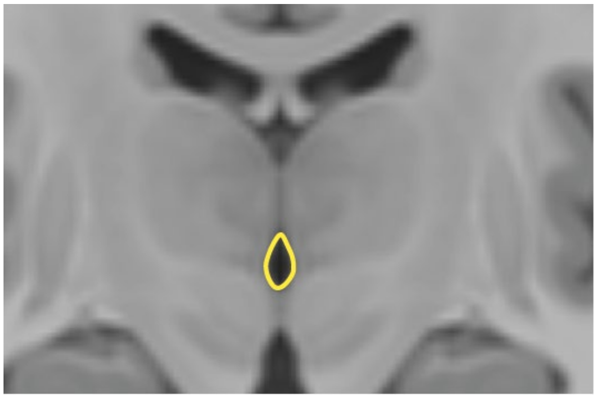

Still more posteriorly, the third ventricle will become more restricted in size, and appear less like an upright rounded column, and more like an oval.

Continue to segment until the superior portion of the ventricle opens up (see below). When it does, careful inspection reveals a membrane that forms an inverted V shape between the separating gray matter on either side and serves as the superior border of the third ventricle (white arrows, left). Adjust brightness and contrast, if necessary to visualize this border. Segment as shown below (right).

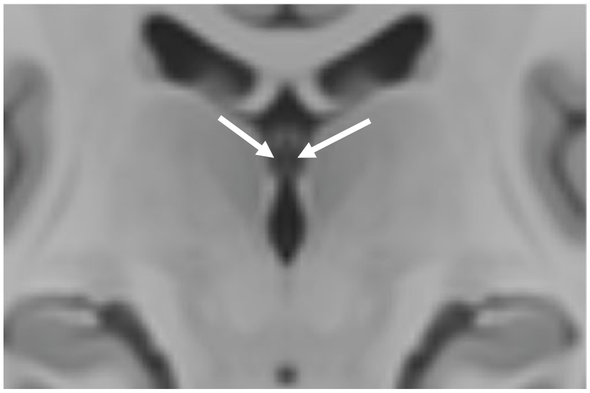

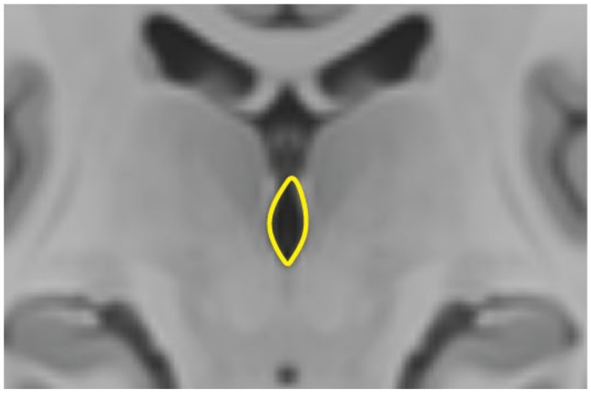

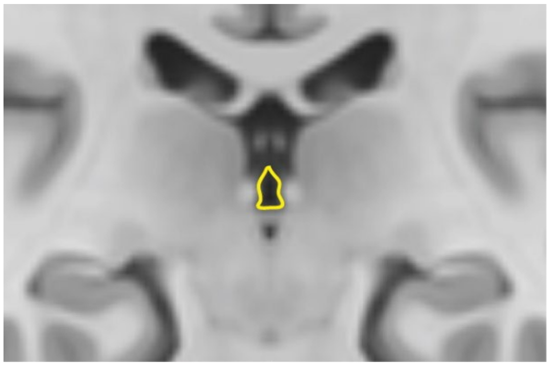

Shortly after the above section, a horizontal white matter commissure will divide the space in two. The space below this posterior commissure is considered the beginning of the fourth ventricle and should not be segmented as the third ventricle. The space above the commissure is the continuation of the third ventricle. The same membrane as described above is present and should be used as the border.

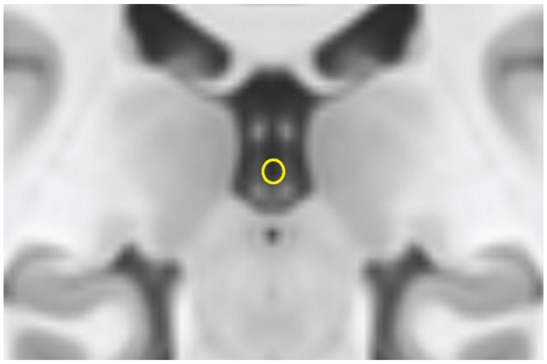

The most difficult border of the third ventricle to conceptualize and segment is a small extension above the midbrain. This extension is present for 2-4 sections after the posterior commissure, and appears in the location to the right. It is separated from the surrounding subarachnoid space by a small membrane, but this may be difficult to see. To visualize the membrane, adjust the brightness and contrast, and view the sagittal and axial sections.

Return to the section displayed below. Turn off the thresholding mask and extend the segmentation to include the choroid plexus, as depicted below. Do not extend into the space(s) above this structure; these angled spaces are the transverse cerebral fissure.

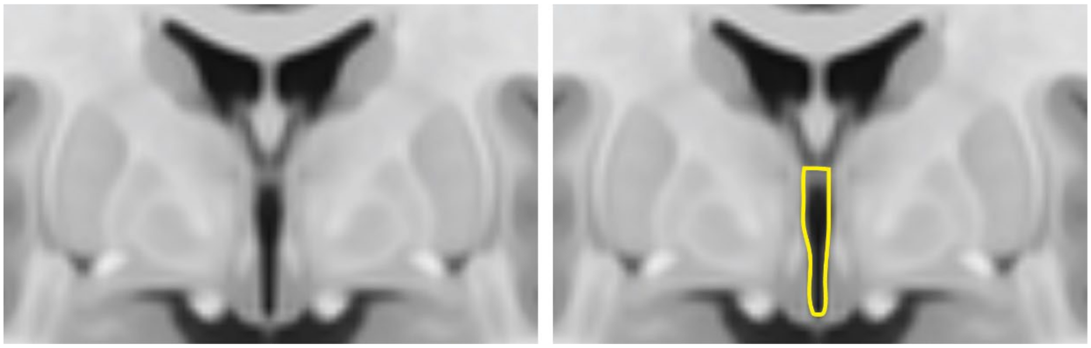

Review the segmentation on the axial, sagittal, and 3D views. Keep in mind that the third ventricle is a small structure, and the ***Threshold*** tool may not be optimal in some locations and in certain brains. When the segmentation is reviewed, make sure the Opacity box is not checked (use the Segmentation Module), and minimize the line width. As indicated above, use your anatomical judgment, and intervene when you decide the segment is too generous or too conservative.

Transverse Cerebral Fissure

The transverse cerebral fissure (TCF) is a cisternal subarachnoid space that extends anteriorly from the subarachnoid space above the cerebellum into the center of the brain. It is filled with CSF and while it apposes and forms a border with the lateral and third ventricles, there is no direct communication between the ventricular CSF and the CSF in the TCF. In middle coronal sections, the TCF is roughly diamond-shaped, with its inferior borders formed by the thalami. The superior borders of the TCF are formed by the inferior borders of the fornices. In some brains, the TCF sends lateral extensions underneath the fornices. More anteriorly, the TCF is split by the descending columns of the fornix into two small vertical slits that extend rostrally for a short distance before ending. The TCF is labeled as the CSF segmentation ROI.

Segmentation of the TCF is first performed in coronal sections near the middle of the brain where the thalami are prominent. The section below shows such a coronal section without (left) and with labels (right). The lateral ventricle is depicted in purple, the third ventricle in red, and the TCF in yellow. The ***Threshold*** tool is used to determine the border of the TCF (approximate location indicated by the black rectangle). Note that the border of the TCF abuts the lateral ventricle (purple).

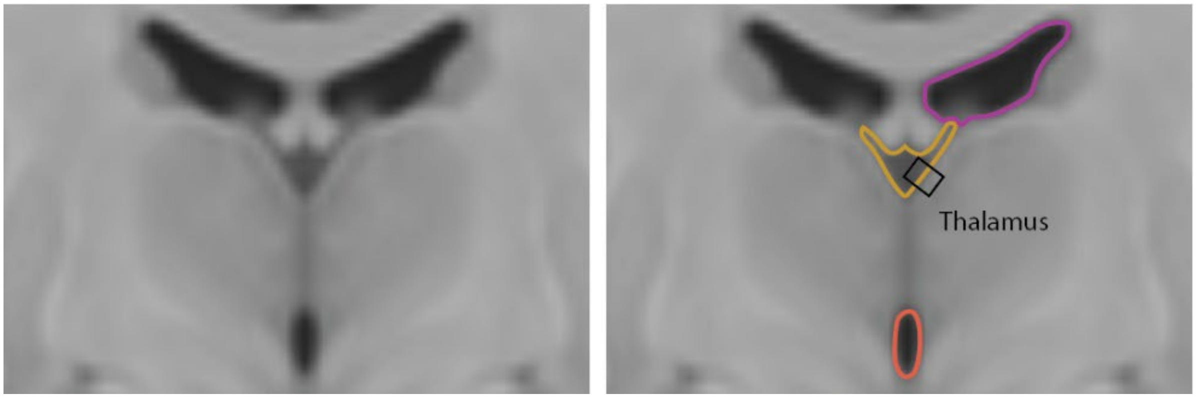

Several blood vessels (i.e., internal cerebral veins) are contained within the TCF and their voxels are often more intense than the threshold used to denote the border of the TCF. Thus, several voxels within the thresholded TCF space will typically remain unlabeled after the TCF is segmented. The blood vessels are considered to be within the TCF as a matter of definition and should be labeled. Remove the thresholding mask to label these voxels before moving on to label the next section.

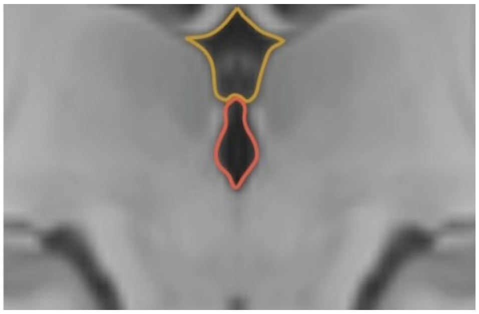

Label the TCF on more posterior sections using the method described above. When the third ventricle (red) is directly apposed to the TCF (left), make sure there are no unlabeled voxels between the two segments (i.e., the TCF should directly abut the third ventricle segmentation).

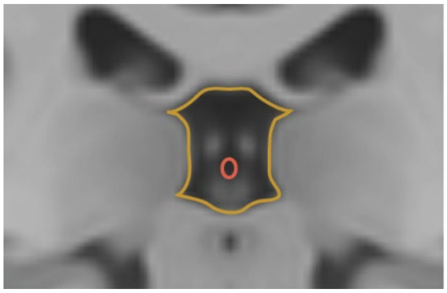

In sections posterior to the posterior commissure, recall that the third ventricle (red) appears as a small bubble within a larger space. This space is the transverse cerebral fissure. Segment as previously, but set the masking selection to 'Outside all segments' to insure the third ventricle segmentation is not overwritten.

End the posterior segmentation of the TCF at the same level the third ventricle ends.

Return to the first section that was labeled. Continue segmenting the TCF in more anterior sections.

Near the beginning of the thalamus, the choroid plexus of the third ventricle (white asterisk below) will be labeled as the third ventricle as described in the third ventricle segmentation procedure. At this level, the TCF will form lateral and superior extensions around the fornix (yellow). Make sure there are no empty voxels between the TCF (yellow) and the third (red) or lateral (purple) ventricle segments.

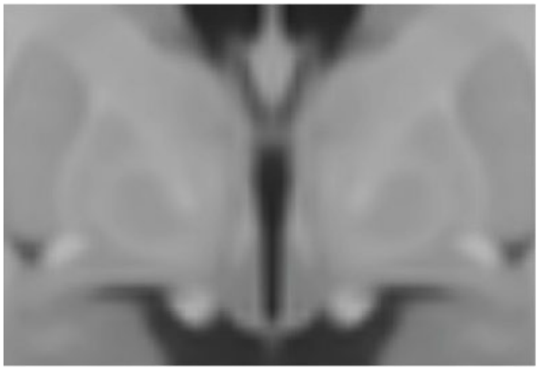

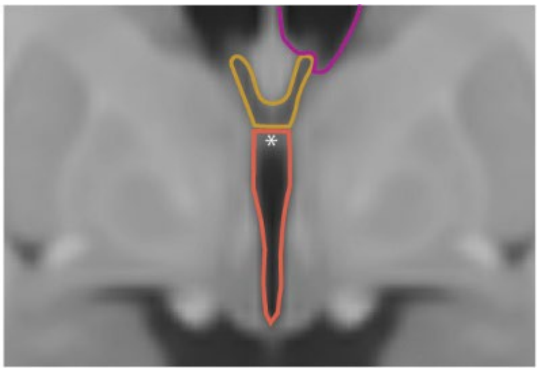

In more anterior sections, the TCF will separate into two small slivers, one on either side of the fornices (see below in yellow). These anterior wings of the TCF will be difficult to segment using the ***Threshold*** tool due to their small size, so use your anatomical judgment to outline their borders.

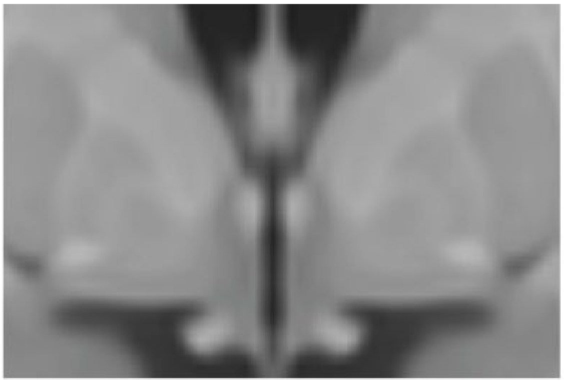

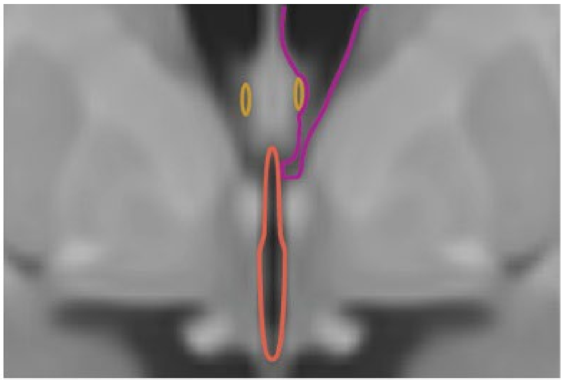

When you have completed segmenting the TCF, review its borders. Look for inaccurate borders as well as unlabeled voxels within the TCF. Keep in mind that, just as in the third ventricle, the borders may not be perfectly amenable to definition with the threshold masking alone. This is particularly true in the anterior portion of the TCF. Accordingly, use your anatomical judgment to determine whether a voxel should be included within the TCF or an adjacent structure (e.g., thalamus, third ventricle).

The Fourth Ventricle

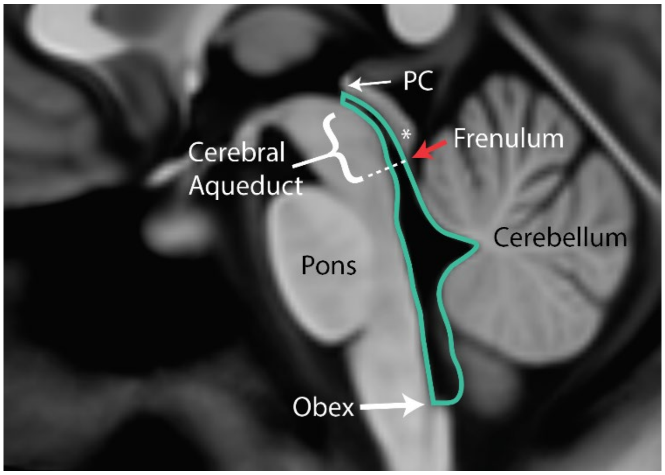

The fourth ventricle is the brainstem component of the cerebral ventricular system. It is connected to the third ventricle anteriorly, and starts as a narrow canal called the cerebral aqueduct under the posterior commissure (PC). This narrow aqueduct comes to an end at the inferior aspect of the inferior colliculus (asterisk) at the frenulum (tip of red arrow). The ventricle then expands into a triangular or tent-like space between the pons and the cerebellum, where the points of the triangle are the frenulum superiorly, the apex of the roof of the fourth ventricle posteriorly, and the obex inferiorly. In traditional anatomy, the cerebral aqueduct and the fourth ventricle are separate entities. However, the CMA convention includes both within the fourth ventricle ROI.

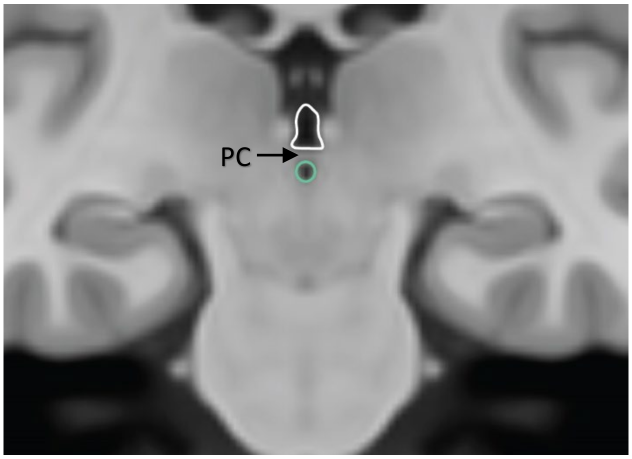

Find the coronal level of the posterior commissure (PC). In this location, the third ventricle was previously segmented as the space superior to the posterior commissure (white outline) and the space inferior to the commissure was unlabeled. Select the fourth ventricle label and segment this space (green) using the ***Threshold*** tool. Keep in mind that in some brains, the fourth ventricle at this level may be so small that the threshold technique may not work well. In this instance, use your judgment and employ the threshold from nearby slices where the ventricular space is more sizable.

Proceed caudally in coronal sections. The fourth ventricle space will lengthen inferiorly. The walls of the superior aspect of the fourth ventricle appear thin and are shaped similarly to a pitched roof. In this location, the threshold masking may not recognize this superior border, and care should be

applied to make certain that the segmentation does not extend beyond this thin superior border. Adjustment of the brightness of the image may be useful in this regard.

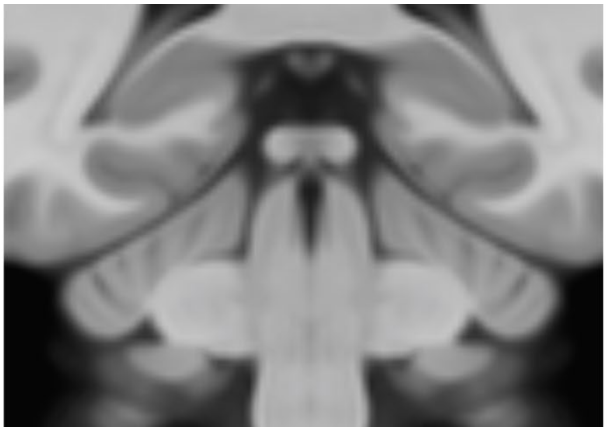

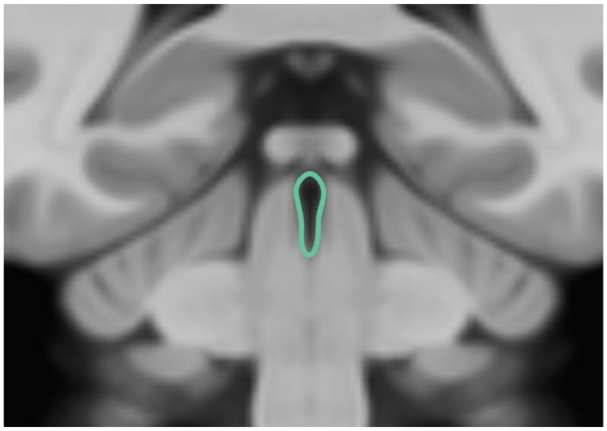

In more posterior sections, the fourth ventricle will expand laterally. The brainstem that forms the superior and lateral borders with the fourth ventricle is dominated by white matter, while the brainstem at the ventral border comprises gray matter. Thus, the threshold values may differ between these gray and white matter brainstem areas and should be adjusted accordingly to produce optimal borders between the fourth ventricle and the brainstem.

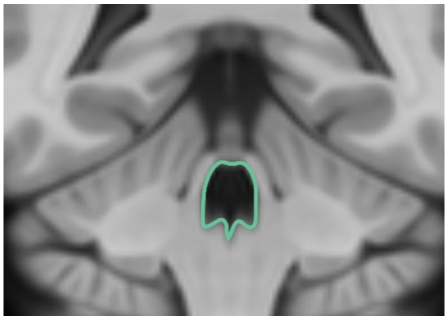

In posterior sections the fourth ventricle will appear as two profiles, superiorly and inferiorly. These profiles are separated by the intervening gray matter of the cerebellum. The superior

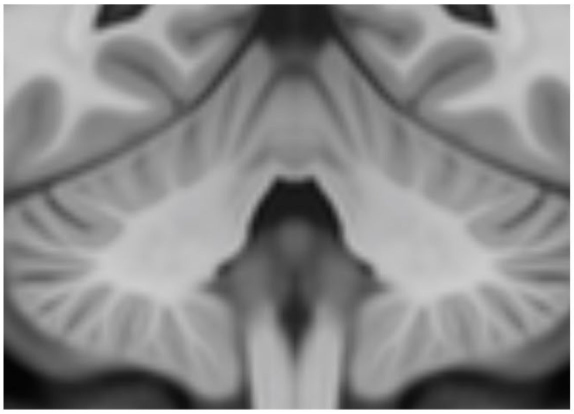

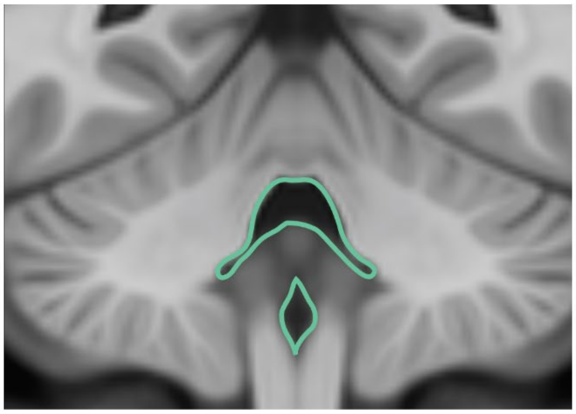

ventricular profile will appear to have lateral extensions, which in some brains may or may not be directly connected to the main cavity of the ventricle.

Segment the fourth ventricle as described above, using a threshold between the gray matter and the ventricular space to determine the borders, and a separate threshold for the border between the white matter and the ventricle.

In more posterior sections, the cerebellar gray matter between the two ventricular profiles will become larger. Notice the V-shape of dark voxels between the superior and inferior ventricular profiles. This comprises the brain exterior rather than a space connecting the two ventricular spaces. Small membranes are visible between the exterior space and the ventricular profiles. Alter the brightness to identify these borders if needed.

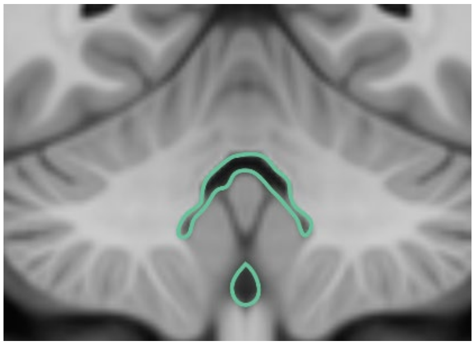

In the most posterior coronal sections, the fourth ventricle will be separated into two parts by the cerebellar gray matter.

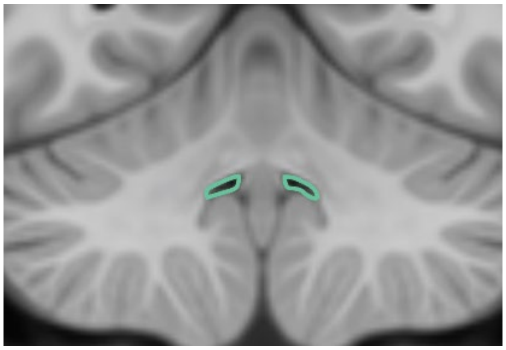

Review the fourth ventricle segmentation on the sagittal sections; identify an image similar to the one displayed to the right. Here, the fourth ventricle has a triangular shape between the brainstem (left) and the cerebellum (right). Note that the ventricular border with the brainstem appears as a straight line. At the inferior end of this line (i.e., the inferior end of the fourth ventricle), a small invagination known as the obex appears (left white arrow). The ventricular segmentation should extend only a line extending posterior to the obex (between tips of the left and right white arrows).

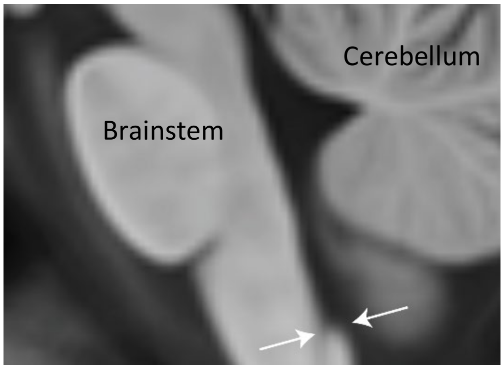

The final portion of the fourth ventricle segmentation involves segmenting the choroid plexus. Recall that the choroid plexus was included in the lateral ventricular segmentation as a matter of definition. This is the case when segmenting the fourth ventricle as well. Use the sagittal images to identify and include the choroid plexus in the fourth ventricle segmentation. Turn off the threshold masking. The left column of images on the right shows sagittal sections of the fourth ventricle from lateral (upper) to medial (lower). The tips of the arrows point to the choroid plexus (CP), which should be included in the fourth ventricle segmentation, as outlined in green in the right column images. A space outlined in blue adjacent to the ventricular segmentation comprises the subarachnoid space and should not be included as part of the fourth ventricle.

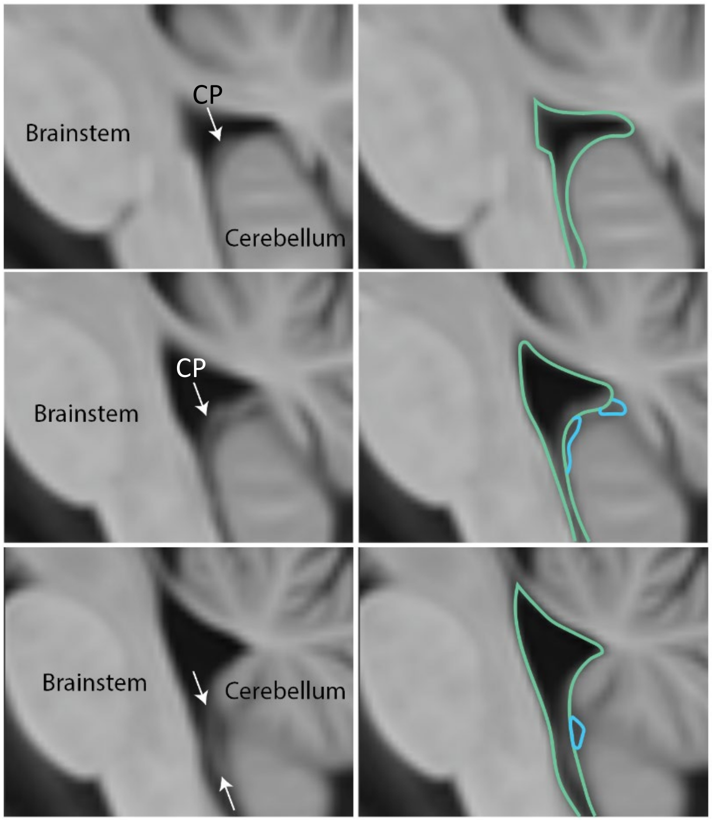

Finally, review the segmentation on the coronal and then the 3D views.

The Nucleus Accumbens

The nucleus accumbens is a gray matter component of the forebrain basal ganglia. It is very similar in intensity to, and contiguous with, surrounding gray matter structures. The borders between the nucleus and contiguous gray matter regions are created using operational definitions based on precise anatomical landmarks and rules.

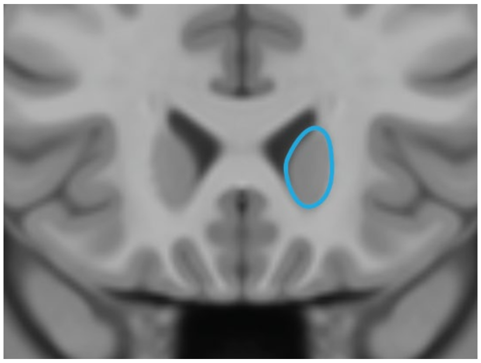

The first step in the nucleus accumbens segmentation is to determine its anterior border. Find a section similar to the one displayed on the right near the anterior portion of the lateral ventricle. The lateral border of the ventricle is comprised of a blue-outlined gray matter structure called the caudate nucleus. The lateral border of the caudate nucleus is white matter. Do not segment anything in this section.

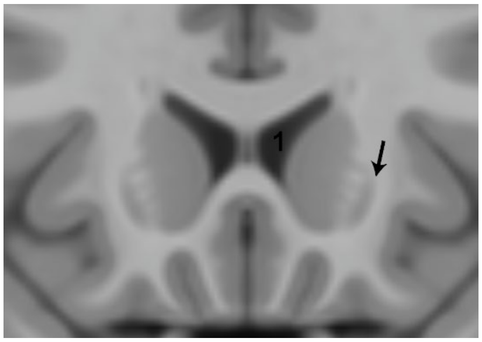

Move posteriorly and observe the lateral aspect of the caudate nucleus. Initially, a few voxels of gray matter will appear as clusters in the white matter lateral to the caudate. More posteriorly, these clusters will coalesce to form a confluent sliver of gray matter (arrow). This is the anterior-most section of the putamen. The identification of this coronal section is important for the present segmentation because it also serves as the anterior-most coronal section of the nucleus accumbens.

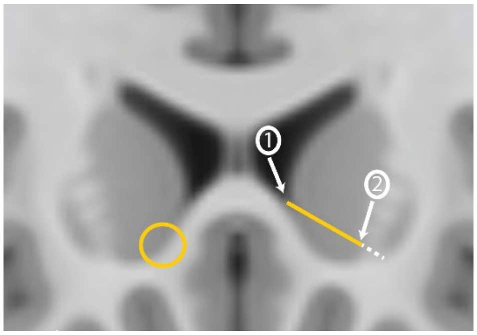

Select the Nucleus Accumbens segment from the list. Under the masking menu, select 'Outside all segments' to avoid overwriting the lateral ventricle segment. Next, obtain a threshold value between the nucleus accumbens and the white matter in the location denoted by the yellow circle (right-hand image, left side of image). Set the Lower bound to 'Minimum' and the Upper bound to 'Average'. Select the ***Draw*** tool and click on the inferior and lateral most point of the lateral ventricle (shown as (1) on image to right). The second point will be left-clicked at the lateral border of the gray and white matter interface (shown as (2) on image to right). To determine the location of this second point, imagine a line extending from the origin (1) through the white matter to the inferior-most border of the

putamen (dotted line). The point at which this line intersects the gray/white matter border of the caudate nucleus (2) is where the second point is selected.

Continue left-clicking inferiorly (or draw with the pen) around the inferior grey-white interface (3), return back to the origin (1), and right-click to close the segment. The threshold mask will insure the segmentation follows the inferior gray-white border of the nucleus accumbens. Even if you 'over draw' this inferior border into the white matter, the threshold masking algorithm will limit the border to the inferior edge of the nucleus.

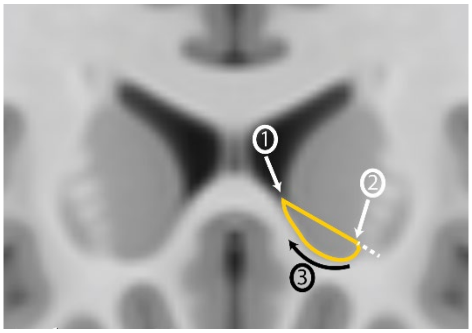

Continue segmenting in more posterior sections using the procedure detailed above. As the putamen becomes larger, continue to use its inferior border to guide the selection of the second point (2 in figure above) of the initial segment line. Remember to take the threshold between the gray matter of the nucleus accumbens and the adjoining white matter in each section.

As the putamen continues to increase in size posteriorly, the white matter separating it from the nucleus accumbens will change in position and disconnect from the inferior surface. This places the putamen directly in contact with the nucleus accumbens. When the white matter moves superiorly, the second point (2) of the initial segmentation line will be located at the midpoint of the inferior surface of this white matter. Next, left-click at the inferior white matter/gray matter interface to create a vertical line between points 2 and 3. Then, as before, draw or left-click around the gray-white interface (4) to connect back to the origin (1).

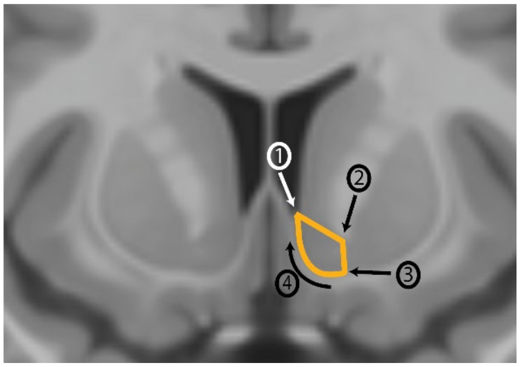

The above picture shows a large distance between 2 and 3 for illustrative purposes. It should be noted that the distance is initially small and grows with more posterior sections. Once there is ***any*** distance between points 2 and 3, the above rule should be applied.

Continue segmenting in more posterior coronal sections. The caudate nucleus will diminish in size, particularly in its inferior aspect. The contrast of the gray/white matter border on the medial aspect of the nucleus accumbens will start to diminish (see arrows at right), and the ***Threshold*** tool will be of limited utility. However, the border is still visible and should be consistent in position with previously segmented

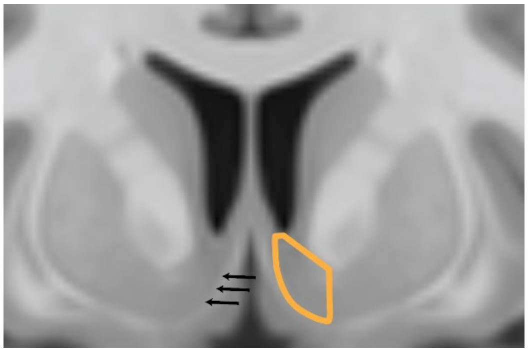

coronal sections. Turn the threshold mask off to draw the medial and inferior border.

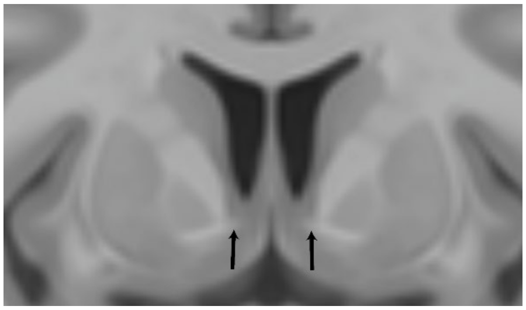

The final stage of the nucleus accumbens segmentation is to know when to stop. In the image to the right, note a horizontal white matter pathway called the anterior commissure extending medially from the putamen. The medial spur of this commissure is indicated by the tips of the black arrows. When this medial spur appears, the nucleus accumbens should no longer be segmented. Keep in mind that some brains are asymmetrical, and the spur may appear on one side before the other side. In these instances, segment the nucleus accumbens on the side in which the spur has not yet appeared; do not segment it on the side in which the spur is present.

Review the nucleus accumbens segmentation on the sagittal and axial sections. The use of the ***Threshold*** tool may occasionally result in the incomplete labeling of intense voxels within the borders of the nucleus accumbens. Turn off masking and segment the unlabeled intense voxels within the nucleus accumbens segment.

The Caudate Nucleus

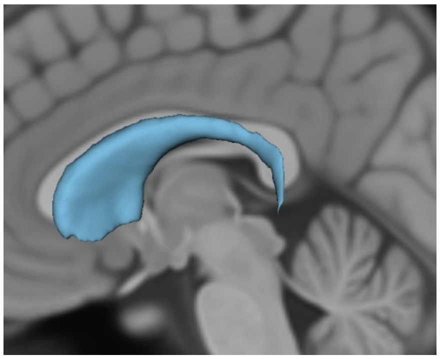

The caudate nucleus is one of the basal ganglia of the forebrain. In three dimensions it has the appearance of a comma, with a large anterior head that tapers to a body, and then to a thin curving posterior bend. The caudate also has a tail, which extends anteriorly from the inferior portion of the posterior bend into the temporal lobe. However, by convention, the tail portion is not segmented. In coronal sections, the medial border of the caudate is the lateral ventricle, and the lateral border is white matter.

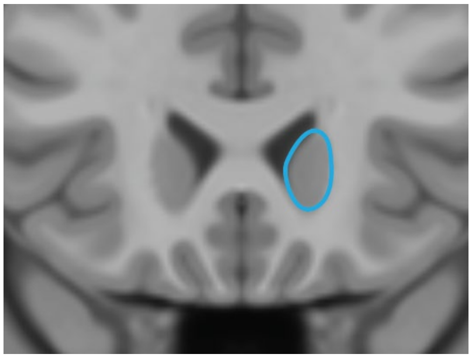

Start the segmentation process by selecting the caudate nucleus label and identifying a section similar to that seen to the right. The border of the caudate with the lateral ventricle will already have been determined in the lateral ventricle segmentation. To insure the caudate segmentation does not overwrite the lateral ventricle, select 'Outside all segments' under the masking tab. Next, use the ***Threshold*** tool to take a threshold value at the lateral border of the caudate with the white matter. Select 'Minimum' as the lower bound, and use 'Average' as the upper bound. Segment the caudate so that its borders appear similar to the blue outline on the image to the right.

After segmenting this section, move anteriorly. Continue segmenting the caudate with the above procedure until the caudate appears to end. The precise anterior border will be specified at the end of the segmentation process for the caudate (see below).

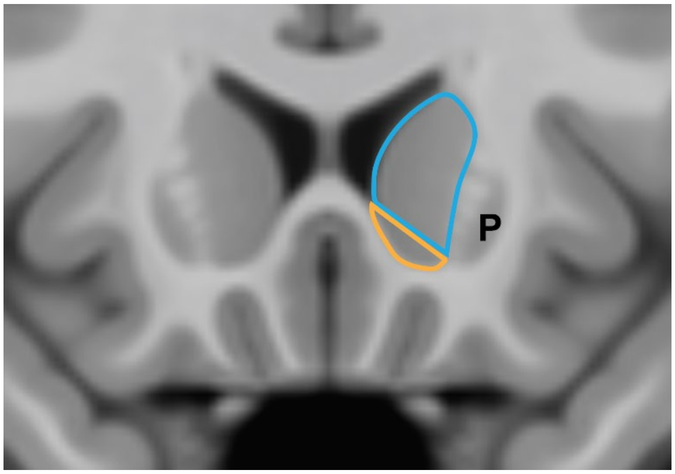

Return to the first segmented section of the caudate nucleus and begin segmenting in the posterior direction. A small gray matter sliver will appear lateral to the caudate. As indicated previously in the nucleus accumbens segmentation guide, this is the putamen (P). At this level, the putamen is separated from the caudate (blue) by the intervening white matter (the internal capsule). The appearance of the putamen triggered the segmentation of the nucleus accumbens (orange). Notice that strands of gray matter extend between the caudate and putamen through the intervening white matter. By convention, these strands are not included in the caudate or the putamen segmentation. Draw a line on the lateral border of the caudate similar to that displayed on the image on the right, which excludes connecting strands of gray matter.

Note: Strands of gray matter between the caudate and putamen will appear throughout the antero-posterior extent of these nuclei, but should not be included in either segmentation.

Continue segmenting the caudate nucleus in more posterior sections. Recall that the nucleus accumbens ends immediately before the anterior commissure. In the section after the anterior commissure ends, the caudate nucleus segmentation will use the anterior commissure (AC) as its inferior border. This slender inferior portion of the caudate nucleus ROI is most appropriately termed the bed nucleus of the stria terminalis. However, this region is included in the caudate nucleus segmentation because of the reliability of this inferior border.

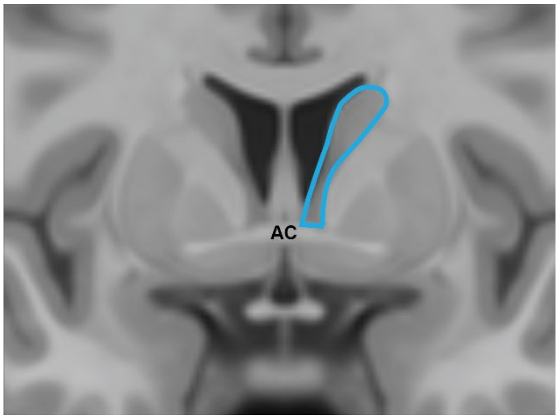

More posteriorly, the slender inferior portion of the caudate nucleus ROI will disappear and the caudate will be located on the lateral aspect of the lateral ventricle. Continue to segment the caudate nucleus by thresholding at its lateral border with the white matter. As before, avoid segmenting strands of gray matter that extend laterally and inferiorly towards the putamen. In addition, do not extend the segmentation to the superior-lateral portion of the lateral ventricle (arrowhead). This location is occupied by a white matter bundle (Muratoff's bundle) that appears less intense than the surrounding white matter and can be mistaken for the caudate nucleus.

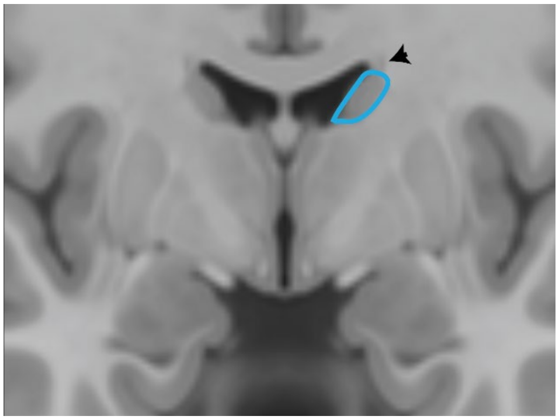

Continue to segment the caudate in more posterior sections using the threshold with the white matter. Again, do not extend the border of the caudate superiorly – the shape of the caudate should be oval rather than a teardrop. With more posterior sections, the caudate becomes smaller and the intensity difference between it and the white matter may be more difficult to visualize in some brains.

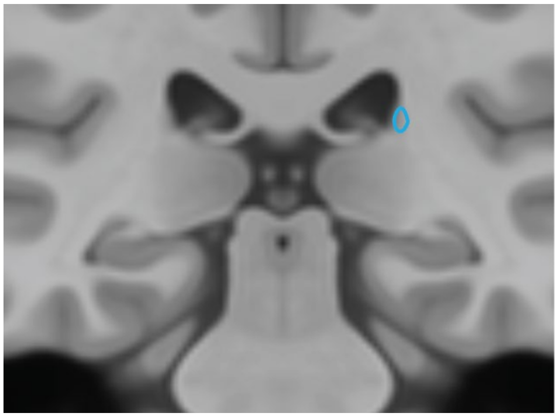

Further posteriorly, the caudate will descend along the lateral border of the atrium of the lateral ventricle. Use the ***Threshold*** tool in this location and pay particular attention to insuring that the caudate nucleus segmentation at this level does not include the choroid plexus of the lateral ventricle (see Lateral Ventricle Segmentation Guide above). In more posterior sections, the caudate will shrink and disappear and the lateral border of the atrium of the ventricle will be white matter.

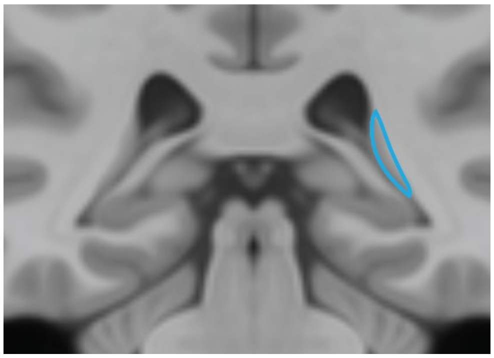

As the caudate nucleus is segmented, there may be voxels inside the caudate segmentation that remain unlabeled when using the threshold mask. These unlabeled voxels are a result of white matter fibers within the caudate that confer an intensity to the voxels that exceeds the threshold mask. Nonetheless, these voxels should be included as part of the caudate nucleus segmentation. To include these voxels, select 'Everywhere' in the masking menu and label them with the ***Paint*** or ***Draw*** tools as caudate nucleus. This process can be performed during the segmentation procedure (i.e., at each section), or after the initial caudate segmentation.

It is important to review the caudate nucleus segmentation on the sagittal and axial sections, as well as on the 3D model. Use this review to also determine whether the segmentation extends into the white matter strands by viewing it in multiple orientations.

Finally, use the reviewing step to evaluate the anterior border of the caudate. In this location, there are voxels that are classified as white matter hypointensities that do not belong to the caudate nucleus segmentation. By using the sagittal and axial sections (right image), you can use the curved shape of the caudate to better identify its anterior border and exclude these hypointensities (arrowhead). Note that the distinction between the caudate and the white matter hypointensities is less clear in brains where the ventricle is more compressed anteriorly.

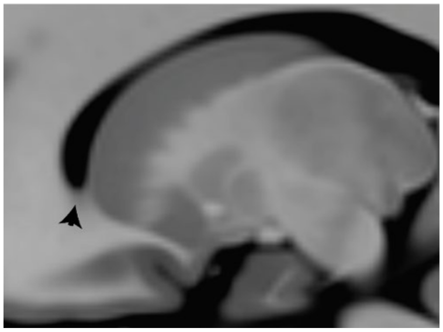

The ***Guide Markup*** tool in the NeuroSegmentation module should be used to draw lines in multiple sagittal sections to delimit the anterior and inferior borders of the caudate nucleus.

These lines will produce transects in the coronal plane (image below, left) that will separate the caudate border from adjoining hypointensities and will thus guide the reliable segmentation of the anterior caudate. To use Guide Markups, select the checkboxes for Red and Yellow Line Views, and select the checkbox for the Green Intersection Views. Select circle as the glyph type and set the scale to 0.50. Press the plus sign to create a Guide Markup. Use multiple clicks in the sagittal view to draw a line similar to that shown below on the right image. Drawing the inferior border of the caudate in multiple sagittal sections will produce transects in the coronal plane. In the image to the left (below), the green and blue represent intersections of the sagittally drawn lines. These intersections represent the borders of the caudate, and are used to guide the segmentation of the caudate borders in the anterior and inferior caudate.

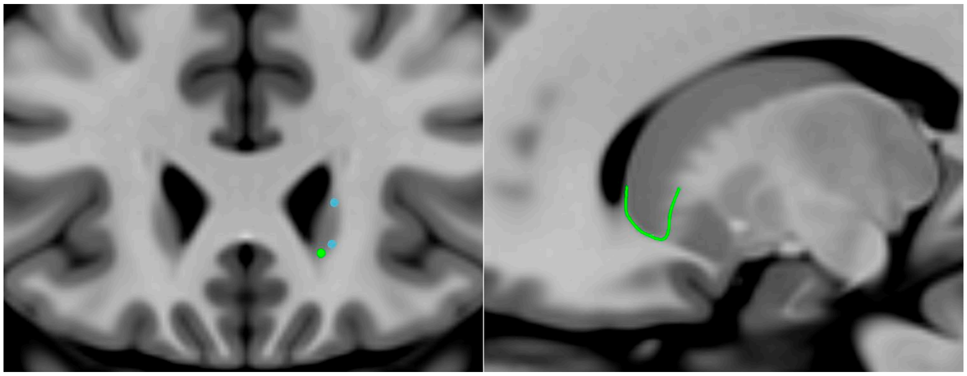

The Putamen

Along with the caudate nucleus, the putamen is one of the principal basal ganglia of the forebrain. It is considered a subdivision of the lenticular nuclei (the other division being the globus pallidus). In the axial view (see image on right), the lenticular nuclei resemble a rounded triangle. The lateral part of the triangle is the putamen (P; pink outline) and the medial portion is the globus pallidus (G.P.; blue outline).

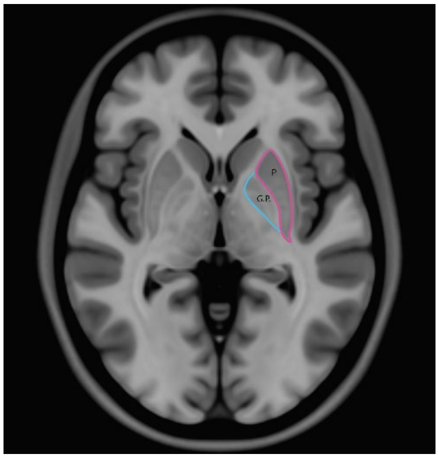

On coronal sections, the putamen is lateral or inferior to the caudate nucleus and separated from it by the white matter of the internal capsule. Bridges of gray matter may extend through the internal capsule to connect the caudate with the putamen. As described previously, these voxels of gray matter are not included in either segmentation. The putamen is larger anteriorly, and tapers towards the back of the brain.

The first, anterior-most section of the putamen should be segmented when the islands of gray matter lateral to and separate from the caudate coalesce into a thin sliver. Recall that this section also defines the first section of the nucleus accumbens. Use the ***Threshold*** tool to segment the putamen. Since the histograms generated by the ***Threshold*** tool depend on the number of selected voxels, the histogram may be unclear at this level. In this case, advance to the next most posterior section to acquire a histogram where the putamen is larger. Then return to the anterior-most section of the putamen to segment it using this threshold.

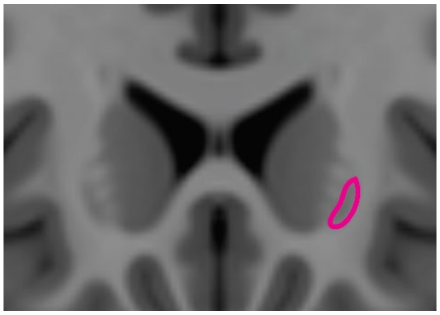

Continue to segment the putamen on more posterior sections. Create a smooth medial line with the internal capsule to avoid segmenting the gray matter bridges. Be sure to set the masking option to 'Outside all segments' to avoid overwriting the nucleus accumbens. In some brains, there are voxels within the putamen that are considerably intense and may not be labeled with the ***Threshold*** tool. Label these intra-putamen voxels by turning off the mask and selecting them with the ***Draw*** or ***Paint*** tools.

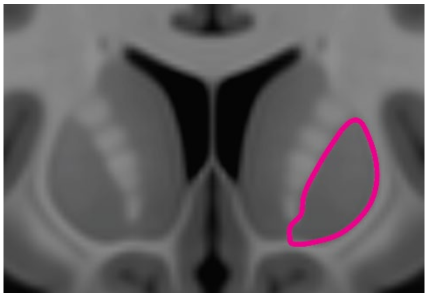

More posteriorly, the globus pallidus (G) will appear medially to the putamen. The anterior commissure (dotted black line) will also be present. An inferiorly-directed vertical line from the lateral border of the anterior commissure comprises the medial border of the putamen at this level.

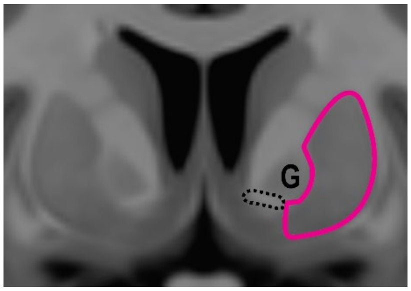

More posteriorly, the globus pallidus (G) will increase in size and push the putamen laterally. A large branch of the middle cerebral artery (asterisk) will typically be present at the inferior border of the putamen: do not segment this blood vessel as part of the putamen. Throughout the anterior-posterior extent of the putamen, a small, vertical gray matter structure called the claustrum (between the tips of the white arrows) will be separated from the putamen by a thin strip of white matter (external capsule). Be sure not to include the claustrum or the external capsule in the putamen segmentation.

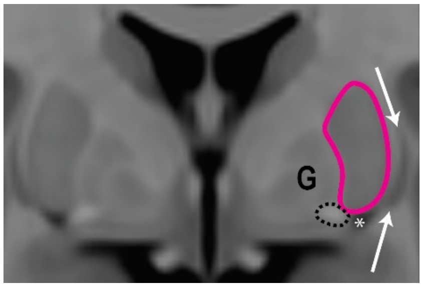

When the blood vessel disappears, the putamen extends inferiorly and in some instances will abut the amygdala (A). Do not include this inferior portion until a clear strip of white matter separates the two structures (between the tips of the white arrows). This inferior portion of the putamen is referred to as the tail of the putamen.

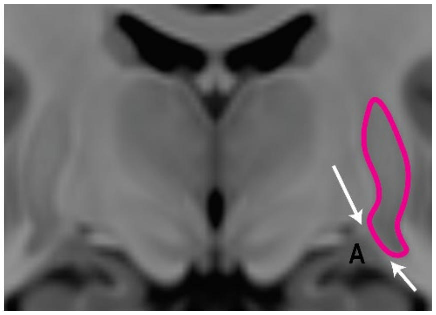

Continue to segment the putamen as it diminishes in size posteriorly. As with the anterior-most section, segment the putamen until the sliver breaks into clusters.

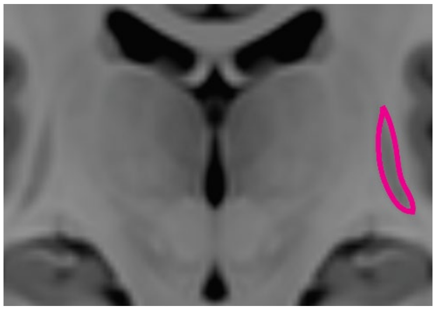

Check the segmentation on axial and sagittal views.

The Globus Pallidus

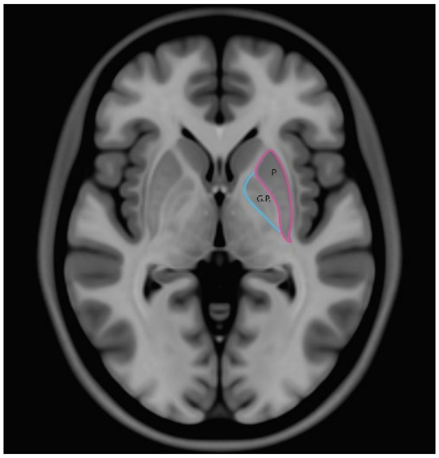

The globus pallidus is the region of the lenticular nuclei that forms the medial portion of the rounded triangle (G.P., blue outline, right). It is comprised of two subdivisions, the pars internus is more medial to the pars externus, and they are separated by a small slip of white matter (the lateral lamina). The division between the two components are not clear on T1w MRI images, thus they are combined in a single globus pallidus segmentation.

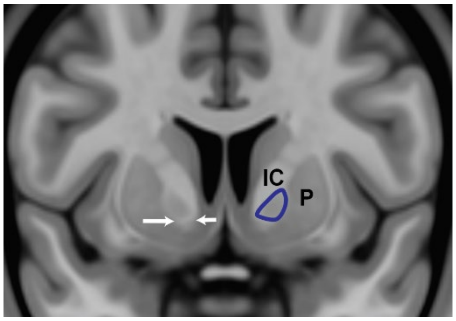

In coronal views, the globus pallidus appears first as a small structure between the internal capsule (IC) and the putamen (P). A sliver of the anterior commissure (between the white arrowheads) forms the inferior border of the globus pallidus. Do not include voxels from the anterior commissure in the globus pallidus. A small slip of white matter, the lateral lamina, is between the pallidum and the putamen; this should be segmented as part of the globus pallidus. In some brains, the border between the internal capsule and the globus pallidus is clear and evident. In these cases, use the ***Threshold*** tool in conjunction with the ***Draw*** tool. In other cases, the border is more obscure. Here, use the ***Draw*** tool without thresholding.

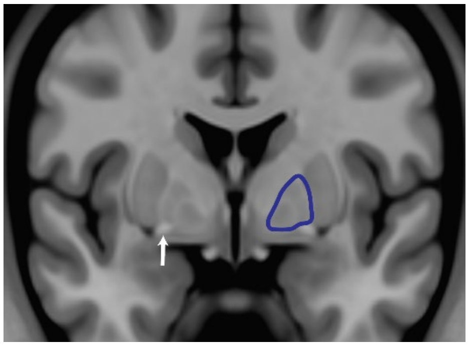

In more posterior sections, the anterior commissure moves laterally (white arrow), and medial part of the globus pallidus extends inferiorly. It may be necessary to slightly adjust the brightness or contrast (via the Window Level button) to optimally visualize the globus pallidus.

More caudally, the putamen (P) will move laterally, and the globus pallidus will diminish in size.

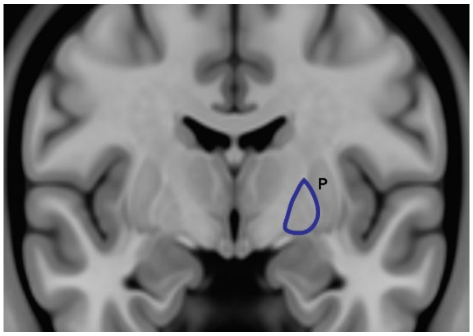

The globus pallidus continues to get smaller in more posterior coronal sections. To be precise about the caudal border of the globus pallidus, it is useful to examine it in the axial sections.

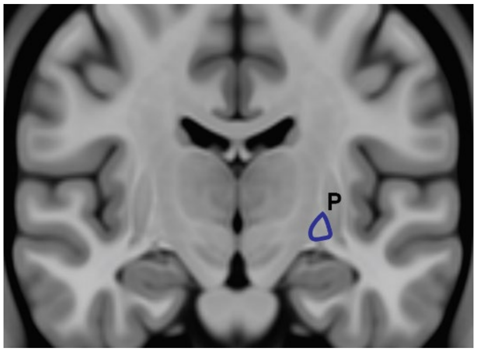

Identify an axial section similar to that seen on the right. In this section, anterior is to the top of the image and posterior is at the bottom. The caudate (C) and putamen (Pt) are separated by the anterior limb (A) of the internal capsule (dotted line), and the globus pallidus is separated from the thalamus (T) by the posterior limb (P) of the internal capsule (dotted line). Note that the border of the globus pallidus with these white matter structures is clear. Also, notice that the posterior end of the globus pallidus is evident.

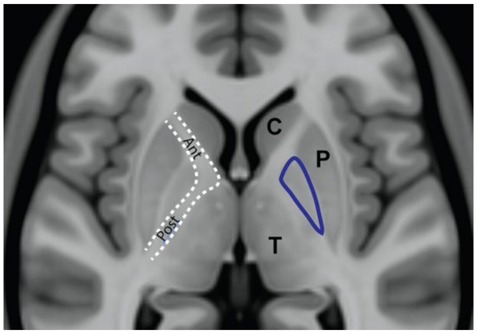

Continue evaluating the medial and posterior borders of the globus pallidus as you proceed in the inferior direction (towards the brainstem) in the axial view. Notice the anterior commissure (white arrow) and make sure it is not included in the globus pallidus segmentation.

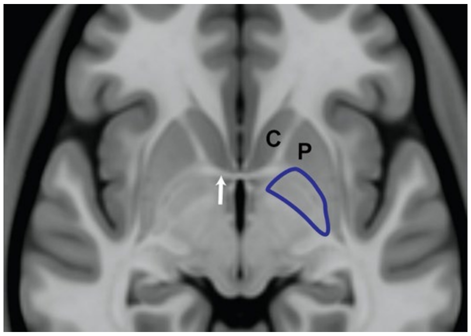

Continue in the superior direction in the axial to complete the segmentation.

Use the axials and coronal sections along with the ***Crosshair*** tool to clearly demarcate the posterior and inferior borders of the globus pallidus. Review using the sagittal series as a final step.

Given the difficulty with current MRI to reliably separate the basal forebrain and/or ventral diencephalon from the ventral (subcommissural) portion of the globus pallidus (i.e., ventral pallidum), this portion is not included in the globus pallidus segmentation.

The Brainstem

The brainstem comprises three parts shown in the figure below (left): the midbrain (Mid), the pons and the medulla (Med). On a mid-sagittal section, the pons is characterized by an anterior bulge. The medulla is inferior to the pons and is continuous with the spinal cord (SC). The midbrain is superior to the pons and is continuous with diencephalic forebrain structures such as the thalamus and the ventral diencephalon.

Unlike other structures, the segmentation of the brainstem begins in the midsagittal plane. The view is then changed to the coronal plane, and the midsagittal segmentation is used to define the superior and inferior brainstem borders. This process is detailed below.

First, identify the midsagittal section. It will appear similar to the image below (left) and will typically appear in the yellow window in 3D Slicer.

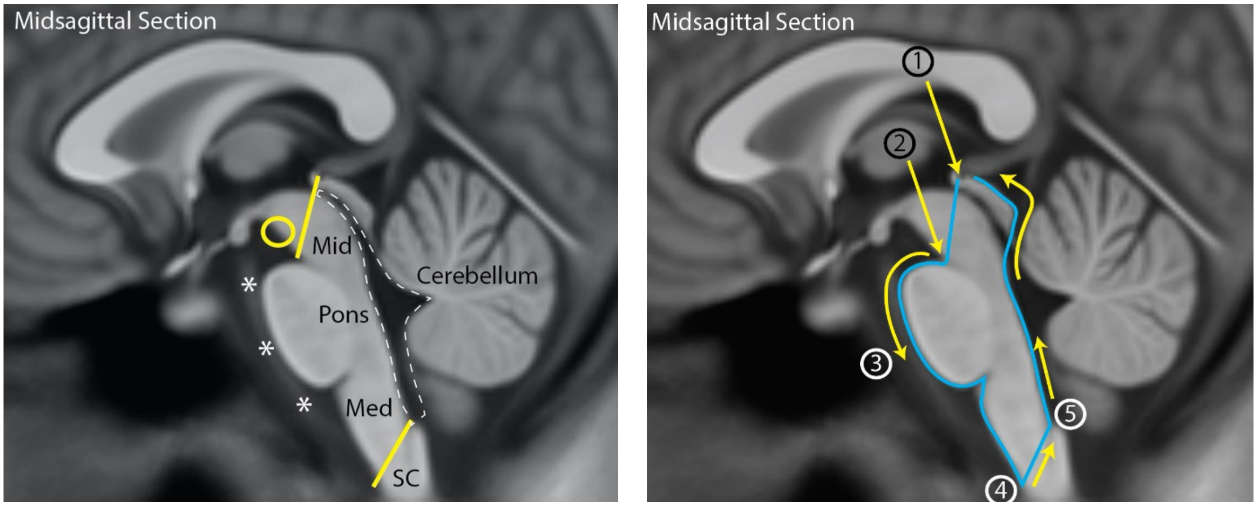

To begin the segmentation, generate a threshold between gray matter and the exterior of the brain (dark voxels). A good place to do so is indicated above by the yellow circle (left image). Be sure not to include blood vessels in the threshold estimate (white asterisks in the image above). Select 'Average' as the Lower limit, and 'Maximum' as the Upper limit. Make sure the masking is set to 'Outside all segments' to avoid overwriting the fourth ventricle segmentation. Next, select the ***Draw*** tool. Your first click should be immediately behind the posterior commissure, which is the small white structure at the tip of arrow #1 (right image). This initial point should be in the dark voxels in back of the posterior commissure. The second click will be at the tip of arrow #2. This is the prepontine or interpeduncular fissure between the midbrain and the pons. This second click will form a straight line through the brainstem and reflects the cut a neuropathologist uses to separate the brainstem from the forebrain. Next, make a series of clicks close to the border of the brainstem with the brain exterior. Be sure not to include blood vessels. Proceed around the pons (#3) and down the medulla until there is a slight change in the angle of the medulla (#4), the pyramidal decussation. Click at this point, and then click in front of the obex, the inferior portion of the floor of the fourth ventricle (#5 on right image). From here, continue drawing the border superiorly along the fourth ventricle (the fourth ventricle is shown as a white dashed line on the left figure above). Proceed anteriorly between the cerebellum and the posterior aspect of the roof

of the midbrain (yellow curved arrow) until you arrive at the point where you began. Press Enter or right click with the mouse to enclose the segment. Examine any unlabeled voxels between the fourth ventricle and brainstem and assign them to one or the other ROIs based on their intensity.

Next, go to the coronal view, which is the green window in 3D Slicer. Find a section similar to the middle panel below. This coronal section represents one of the most anterior sections through the brainstem. The left panel shows a 3D brainstem overlaid on a midsagittal section. The vertical yellow line in this panel represents the coronal section through the brainstem as illustrated in the middle section below. The blue vertical line in this middle image represents the segment previously drawn on the midsagittal section but shifted by 90 degrees. Thus, the planar appearance of the segment in the sagittal plane now appears as a vertical line in the coronal plane. This vertical line guides the segmentation of the brainstem (right panel). Use the Threshold tool in conjunction with the ***Draw*** tool (the pen is useful here) to segment the brainstem. In more anterior coronal sections, the brainstem will be completely disconnected from the rest of the brain. Make sure you move anteriorly until the brainstem disappears to insure you have segmented it all.

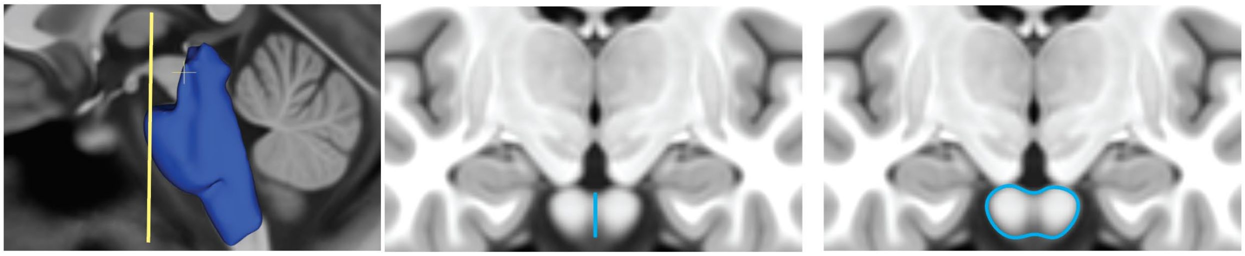

More posteriorly in the coronal series, the brainstem will clearly connect with the rest of the brain. The vertical blue line (middle panel below) will indicate the location of the brainstem (from superior to inferior). The superior border of the brainstem is defined by convention as a line connecting the lateral aspect of the interpeduncular fossa (tip of left red arrow, middle panel) with the lateral invagination of the brain surface (tip of right red arrow, middle panel).

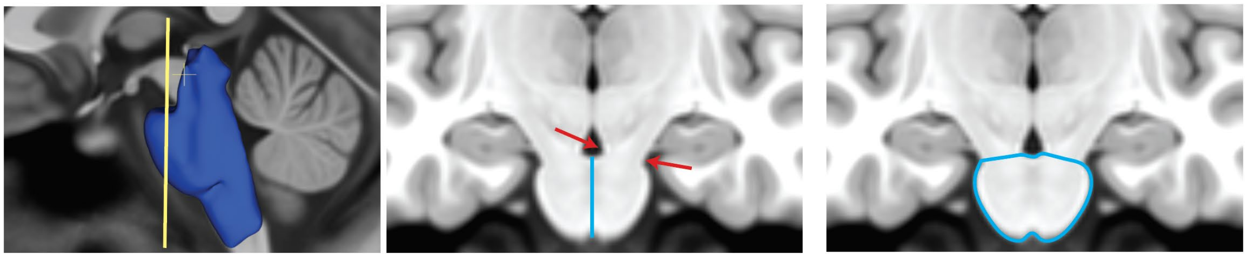

Once the interpeduncular fossa ends, the upper portion of the vertical brainstem line will end in the middle of the brain (middle panel below). When this occurs, draw a horizontal line perpendicular to the top of this vertical line to define the upper limit of the brainstem on this slice (right panel below). Remember in each section to take the threshold of the brainstem with the outside of the brain.

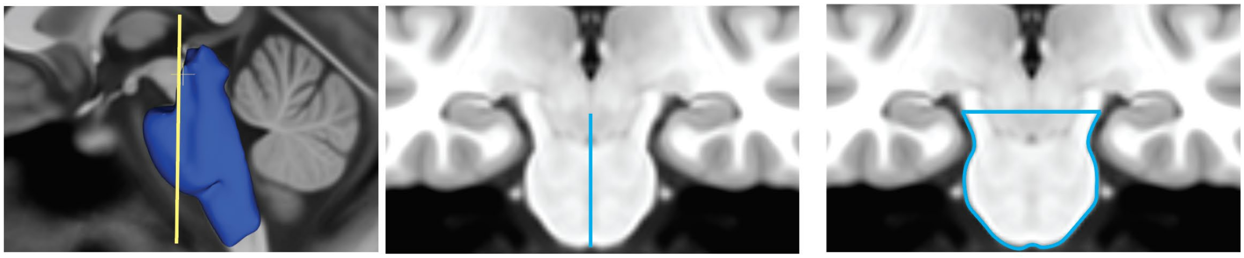

In more posterior sections, the top of the vertical line ends immediately below the cerebral aqueduct (green arrows in middle image below). Draw a line from this point to the upper inflection points of the lateral brainstem surface (tips of upper red arrows in middle image) on either side. Unlike more anterior sections, this approach is necessary because a horizontal line drawn at this level would no longer end at the exterior surface of the brainstem. In this coronal section, also notice that the lateral borders of the brainstem are vertical lines that cut into the brainstem. These lines are guided by inflection points that appear ventrally (two lower red arrows in middle image) and separate a fiber tract called the middle cerebellar peduncle (red asterisks, right image below) from the main part of the brainstem. Note that at this level, the root of the fifth cranial nerve (trigeminal nerve) penetrates the lateral edge of the middle cerebellar peduncle and can often be observed as small bumps (immediately lateral to red asterisks below).

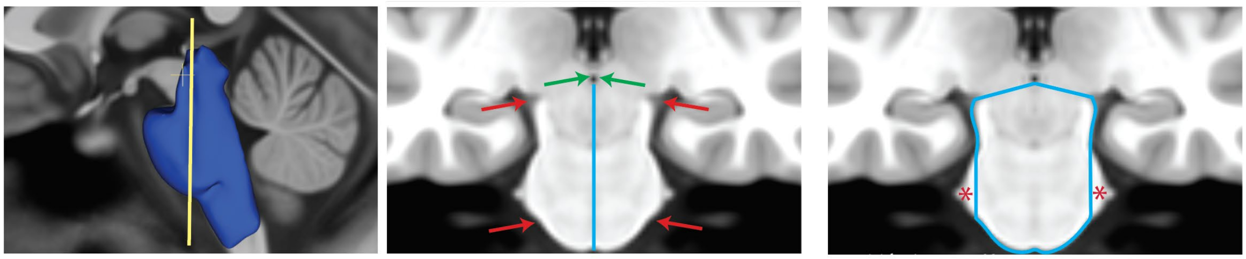

Posterior to the section above, the vertical brainstem line will end above the aqueduct at the brain exterior. Extend the line at its top until the brainstem angles superiorly (tips of green arrows on right image below). At these points, angle lines inferiorly and laterally through the brainstem to reach the inflection points (red arrows). The lateral borders of the brainstem at this level are delineated in a similar manner to that described above.

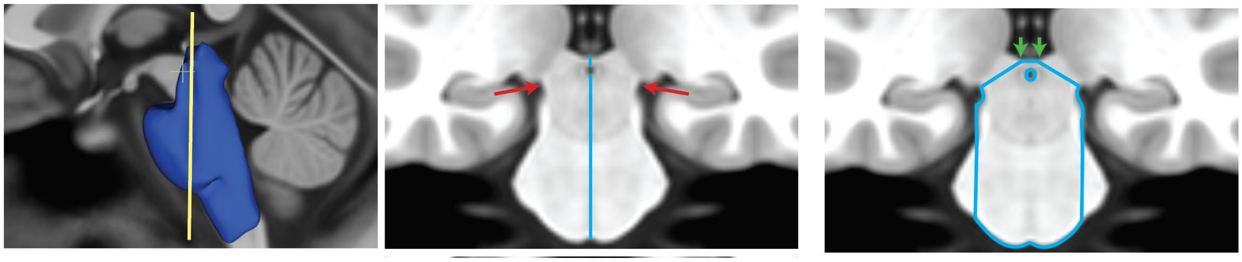

The brainstem will then appear separated from the forebrain, as shown in the image set below. The two paired bumps on the superior aspect of the brainstem, the inferior colliculi, typically are separated from the rest of the brainstem at this level. Be sure to follow these colliculi posteriorly and include them in the brainstem segmentation even if they do not directly connect, as illustrated below (right panel, below).

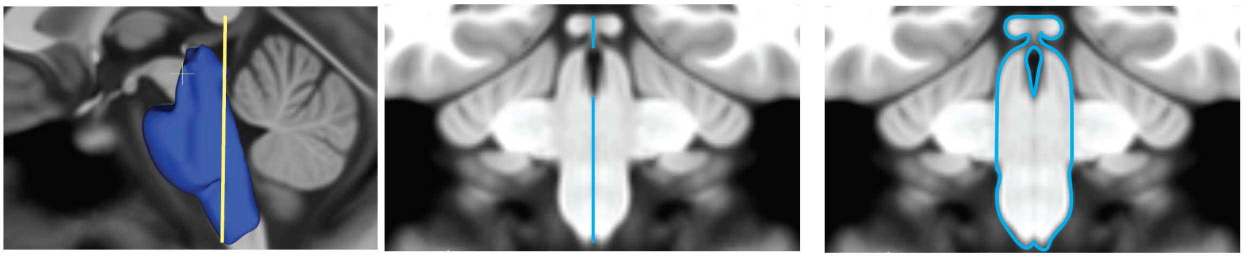

More caudally, the inferior colliculi will disappear, and the vertical brainstem line will not have a component superior to the fourth ventricle. In this instance, segment as illustrated below, using the inflection points (red arrows, middle image below) as borders. At this coronal level, the border between the brainstem and the fourth ventricle comprises mainly white matter, and a single threshold may be used. However, regions of the brainstem contain gray matter (i.e., the facial colliculi indicated by green arrows in the middle image below), and the threshold should be adjusted in these areas accordingly.

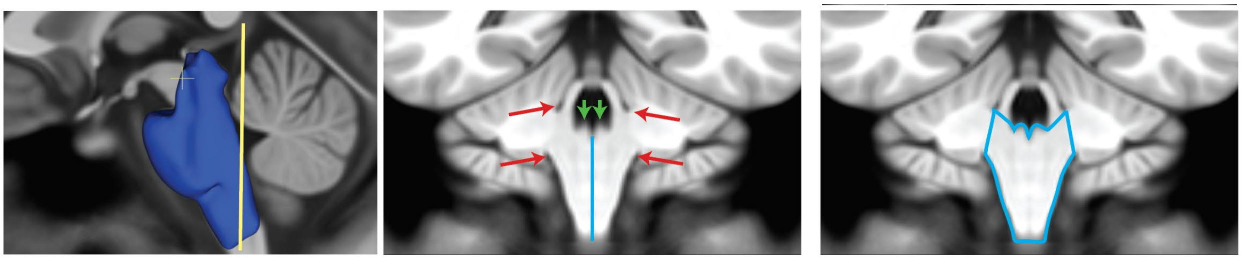

As the brainstem ends posteriorly, the vertical line will progressively shorten and end inferiorly in the substance of the brain (middle panel below). When this occurs, draw horizontal lines to complete the segmentation inferiorly (right panel below).

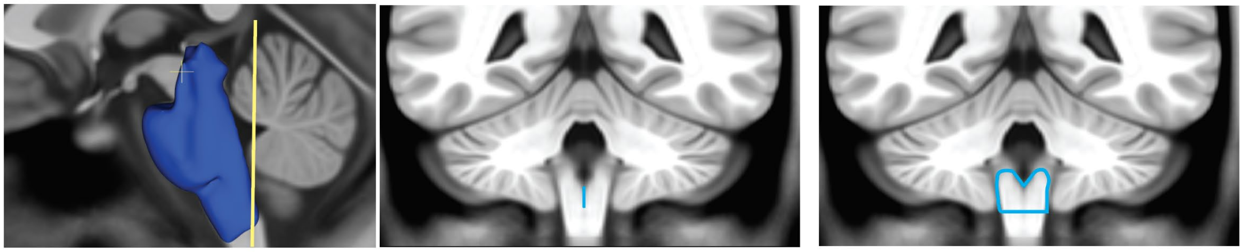

The final step in the segmentation of the brainstem is to return to the sagittal series. Since there may be slight differences in the threshold of the previously segmented fourth ventricle with the brainstem and the threshold used to define the brainstem border, unlabeled voxels may be present at the interface between the two ROIs. Scroll through the sagittal series and label all relevant voxels as needed.

The Thalamus

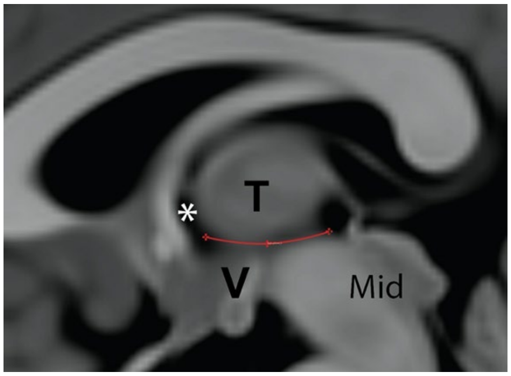

The thalamus is an egg-shaped collection of nuclei. There is one thalamus in each hemisphere. The two thalami are separated by the third ventricle, and more caudally, by the subarachnoid space of the transverse cerebral fissure (TCF). The thalamus is bordered by the foramen of Monro anteriorly (white asterisk), the internal capsule laterally, and it is superior to the midbrain posteriorly (Mid). The inferior border of the thalamus is the hypothalamic sulcus (fissure of Monro, red line) – an evagination in the third ventricle that separates the thalamus superiorly from the ventral diencephalon (V) segmentation inferiorly. It is important to note that the CMA convention does not include two classic thalamic nuclei within the thalamus segmentation, the lateral and medial geniculate nuclei. Instead, these two nuclei are included in the ventral diencephalon segmentation (vide infra).

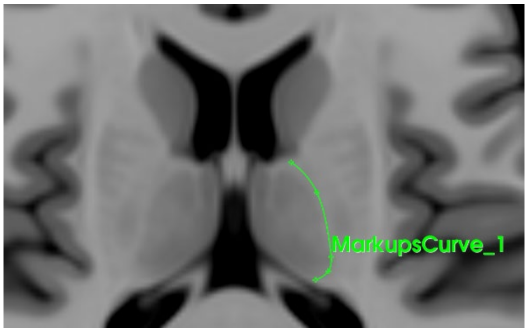

Turn to the axial sections. Here, the ***Guide Markup*** tool in the NeuroSegmentation module will be used to draw guide lines on the lateral border of the thalamus on selected axial sections. On the image to the right, the guide line is shown extending from the anterior tip of the thalamus to its posterior extent. Next, select an axial image several sections inferior to that shown to the right and repeat the process. Only perform this procedure on sections that show a clear border between the thalamus and the white matter (see image to right). Four to five Guide lines should be drawn at approximately equal intervals.

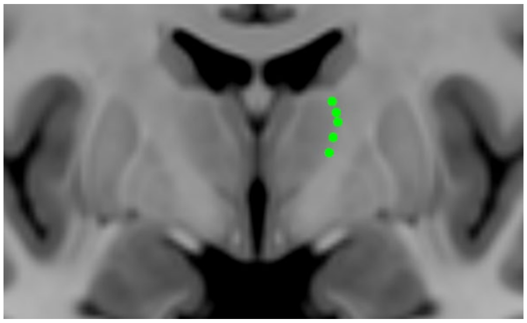

Return to the coronal series. Each of the Guide lines drawn in the previous step will now be represented as dots on the coronal series. In the image on the right, five guide lines were drawn in different axial sections. These now appear as transects (dots) on the coronal series and create an identifiable lateral border of the thalamus on coronal sections.

A critical landmark for the segmentation of the thalamus is the hypothalamic sulcus, and it is important to identify this structure in coronal and sagittal sections. On the coronal section on the right, the hypothalamic sulci are cut in cross section and observed at the tips of the yellow arrows as grooves in the third ventricle (3) laterally. These sulci thus serve as reliable borders between the thalamus (T) superiorly and the ventral diencephalon (V) inferiorly.

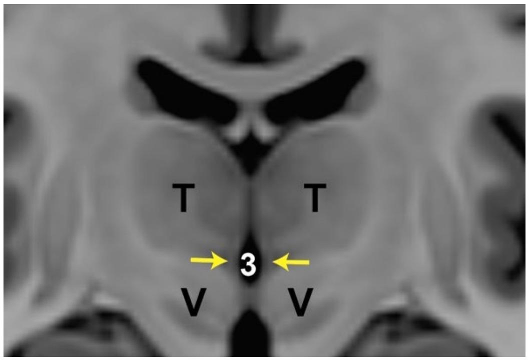

While the hypothalamic sulcus may be evident in some brains in coronal sections, it is often useful to employ the ***Guide Markup*** tool to define it more consistently. To do so, turn to the sagittal view, and draw Guide lines starting with sections that are similar to that shown below. The section on the left is closer to the midline and the sulcus is evident. The section on the right is more lateral and the sulcus is less distinct. At this point, the anterior and posterior portions of the ventricular system continue to be present. Join these points with a curved line that 'cups' the thalamus as shown below.

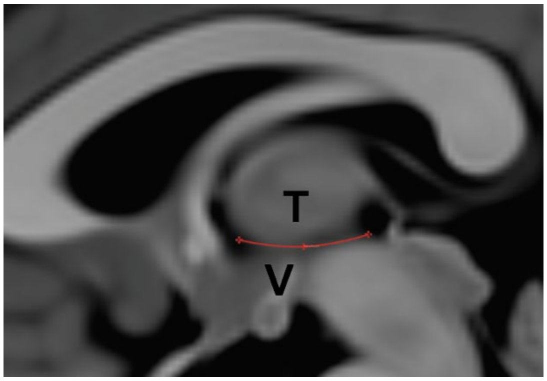

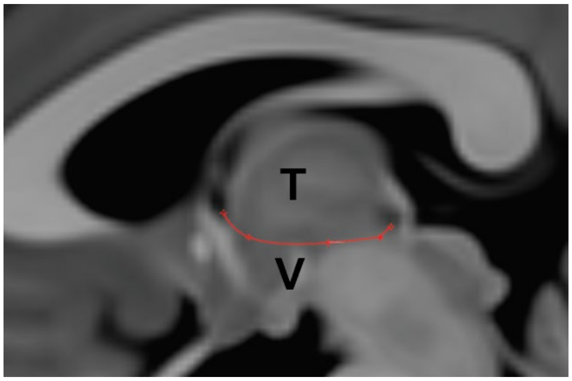

A number of such lines (red) will nicely delimit the inferior portion of the thalamus as transects (dots) on coronal images. These transects can be used in conjuction with those from the axial guide lines (green) to provide a rough border to guide the segmentation of the thalamus. Note that many thalami will have borders that are much less distinct; in these cases, the guide lines are indispensible.

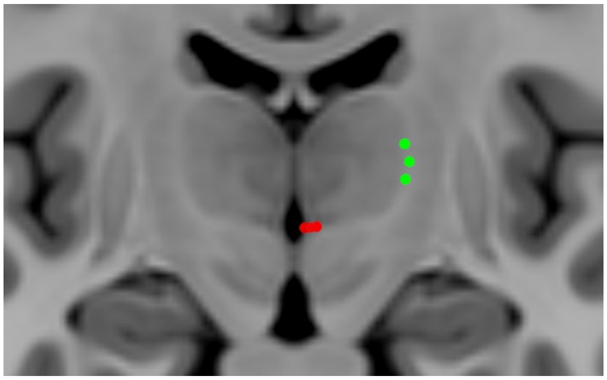

The thalamus may be initially segmented on the midline in the sagittal view where the thalamic borders with the third ventricle (3rd) and transverse cerebral fissure (TCF) are best appreciated. Since these segments have already been delimited, make sure the masking option is set to 'Outside all visible segments' and segment the thalamus as shown on the right. Proceed for 2-3 sagittal sections until the borders are no longer evident. This approach will label the medial portion of the thalamus and help to define the anterior thalamic border. Make sure by examining all views with the ***Crosshair*** tool that the thalamus that is being labeled is the correct one (left or right).

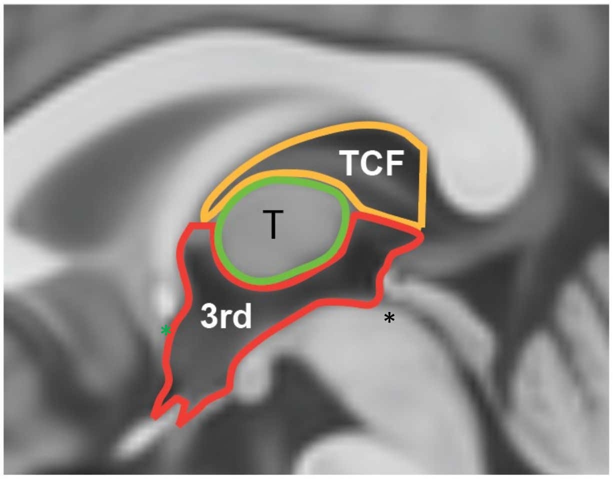

Return to the coronal series and identify the oval-shaped anterior-most section of the thalamus. Portions of the medial aspect of the thalamus at this level may already be segmented from the previous step. The lateral border may be indicated by guide lines. In this anterior location, the thalamus extends from the hypothalamic fissure (yellow arrow) which should be demarcated with the ***Guide Markup*** tool (red dots), to the interface with the inferomedial border of the caudate (C). The thalamus is bordered medially by the third ventricle (3), the transverse cerebral fissure (asterisks) and the lateral ventricle. The lateral border adjoins the internal capsule (IC). Given the myelin content, the ***Threshold*** tool may not be useful in specifying this lateral border of the thalamus, and a ***Draw*** tool without masking should be employed. Use the Guide lines to delimit the lateral border.

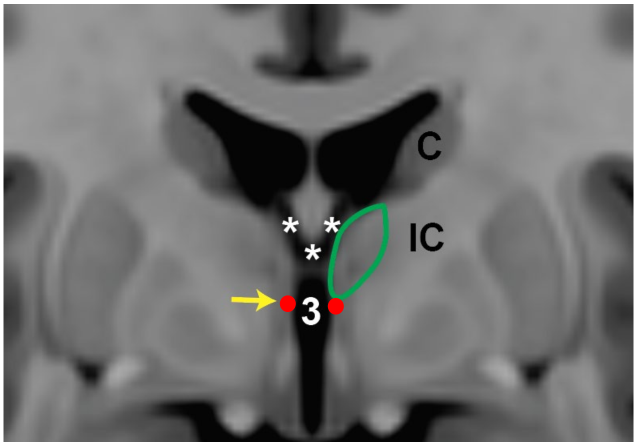

In more posterior sections, the thalamus increases in size, but the same anatomical relationships hold (labeling as in the above section). Note that there is a bump in the medial and superior aspect of the thalamus (tip of blue arrow). This portion of the thalamus has the potential to be confused with the choroid plexus of the lateral ventricle. Cycle through coronal sections at this level to insure this bump is labeled as part of the thalamus ROI.

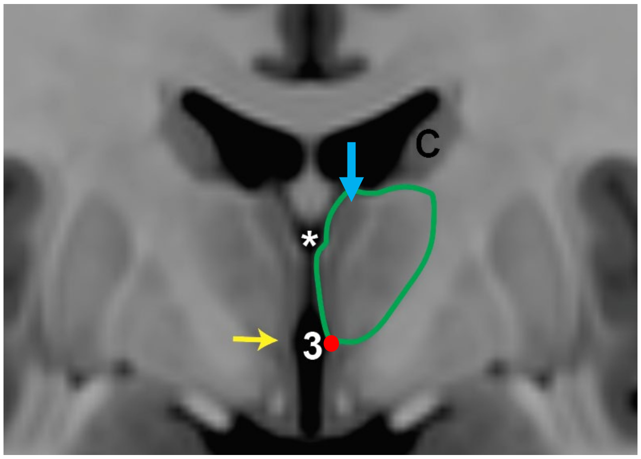

Continue labeling the thalamus posteriorly, using the Guide lines to help specify the thalamic segmentation borders. In the mid region of the thalamus shown to the right, the medial border often adjoins the contralateral thalamus, and it is important not to extend the label past the midline.

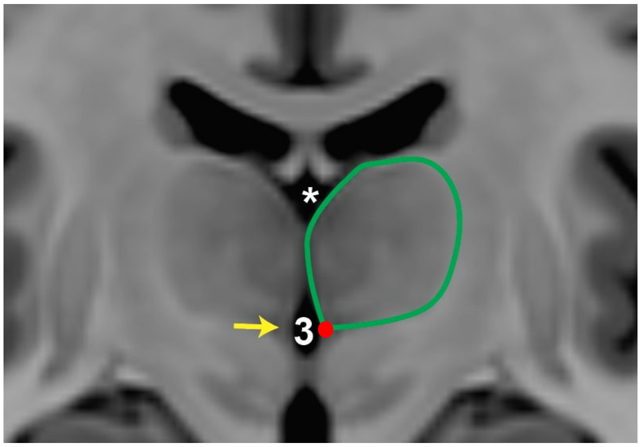

More posteriorly, the third ventricle (3) opens up and a thin membrane (dashed white line) separates the third ventricle from the transverse cerebral fissure (asterisk). At this level, a small white structure, the habenula, appears on the medial aspect of the thalamus (red arrow). This structure should not be included in the thalamic segmentation (it belongs to the VDC segmentation). The inferior margin of the thalamus should curve inferiorly and laterally from the inferior margin of the habenula, as shown in the image to the right. As before, the lateral thalamic border is specified using the transects of the Guide lines. Finally, the lateral geniculate nucleus of the thalamus (L) is present at this level but, by convention, is not included in the thalamic segmentation.

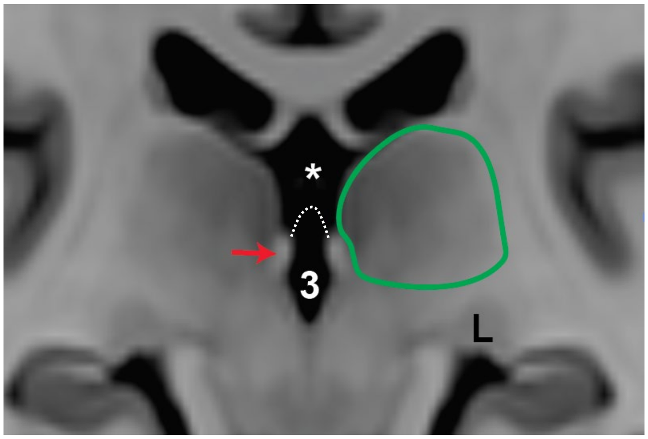

More posteriorly, the thalamus will separate from the brainstem. Here, the Guide lines are especially important in determining the lateral border. For example, the transects of two axially-drawn Guide lines are shown in the image to the right. At this level, or before, the transverse cerebral fissure will have ended, and it is important to use the ***Threshold*** tool to define the medial aspect of the thalamus.

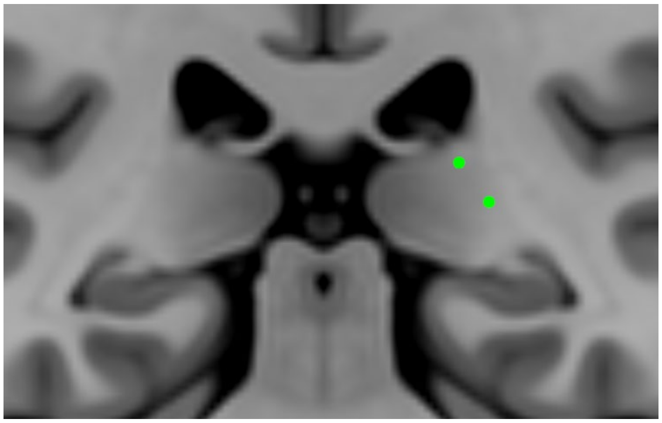

The thalamus will continue to diminish in size and its lateral border will be the white matter bundle of the fornix (F). At this level, the hippocampus will wrap around the posterior end of the thalamus. In some brains, the borders between the two structures are difficult to visualize due to limitations in MRI. Use the ***Crosshair*** tool in conjunction with the axial and sagittal views to draw the correct thalamic border.

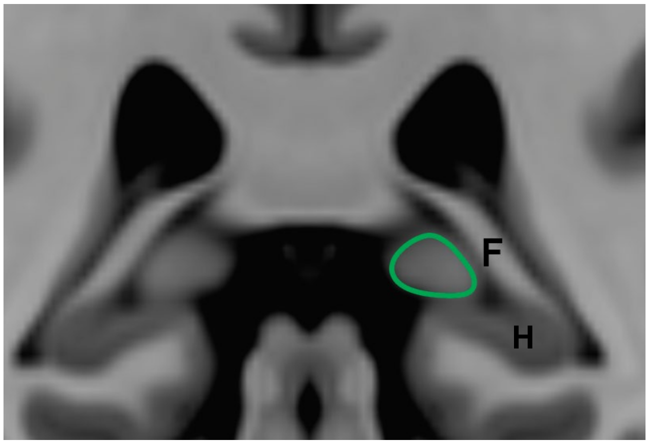

Review the thalamus on all three views and on the 3D view.

Ventral Diencephalon

The ventral diencephalon (VDC) is a group of diverse structures that includes white matter pathways such as the optic tract and the cerebral peduncles, and gray matter regions such as the hypothalamus, the habenula, and the lateral and medial geniculate nuclei of the thalamus. The VDC begins in the coronal section immediately posterior to the anterior commissure and extends to the brainstem posteriorly. The superior border for most of the VDC is the hypothalamic sulcus, which was delimited previously in the thalamic segmentation procedure. The inferior border of the VDC is the subarachnoid space at the base of the forebrain anteriorly and the brainstem more posteriorly. Medially, the VDC is bounded by the third ventricle. The lateral border of the VDC changes throughout the antero-posterior direction, but its maximum extent is defined as the lateral aspects of the optic tract or the lateral geniculate nucleus of the thalamus.

Segmentation of the VDC begins in the coronal section immediately posterior to the anterior commissure (blue asterisk). It should be noted that in previous versions of this segmentation approach, the VDC was presented and segmented as a singular ROI. In this version of the manual, the VDC is subdivided into two portions, the anterior VDC (aVDC, orange), anterior to and including the mammillary bodies (white asterisk), and the posterior VDC (pVDC, pink), posterior to the mammillary bodies.

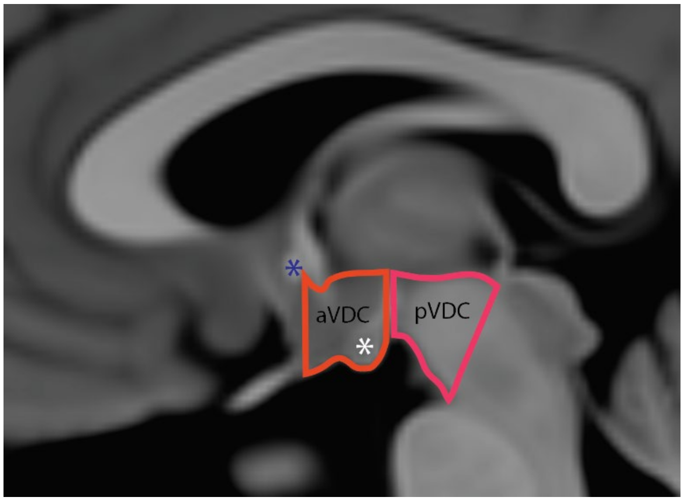

In this section, the fornix (yellow asterisk) is often observed. The fornix is not included in the aVDC at this level. Start by thresholding the aVDC against the CSF space outside the brain (yellow circle); select 'Average' as the Lower bound and 'Maximum' as the Upper bound. Set the editable area to 'Outside all segments' to avoid overwriting the thalamus or the third ventricle. Segment as shown to the right (red outline), extending as far laterally as the white matter (red asterisk). This white matter is the optic tract, and it is the lateral border of the aVDC throughout most of its antero-posterior extent. A separate threshold may be required to produce an accurate border between the outside of the brain and white matter structures of the aVDC such as the optic tract.

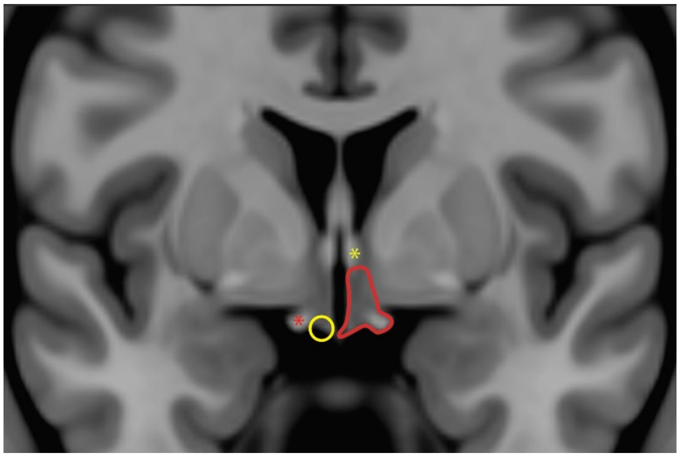

In more posterior sections, the columns of the fornix (yellow asterisk) will penetrate the gray matter of the aVDC and will thus be located inferiorly to the hypothalamic sulcus (yellow arrow), which was delimited in the thalamic segmentation procedure. The lateral aspect of the aVDC is initially specified using a border between the gray and white matter with the ***Threshold*** tool. Given the intermingling of gray and white matter in this region, the ***Threshold*** tool typically produces only an approximate border; this border must then be manually adjusted. It is not unusual to have to delineate the entire lateral border manually, which requires anatomical knowledge of the hypothalamus and adjoining structures. Insure the lateral border of the aVDC does not touch or contact the globus pallidus that was segmented earlier. The optic tract (red asterisk) continues to be the lateral most structure of the aVDC.

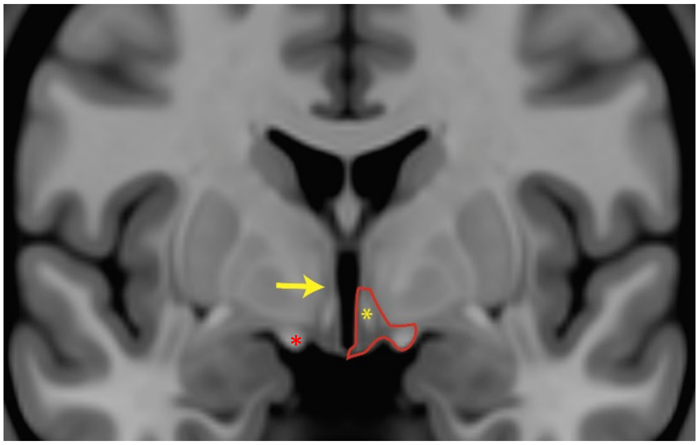

Continue segmenting using this approach, making sure to use the threshold masking for both gray and white matter of the aVDC to obtain appropriate borders. Notice that the optic tract continues to be lateral in the aVDC. In this image, the mammillary bodies (white asterisk) are present at the medial aVDC margin. The curve of the lateral margin is adjusted manually to include ventral gray matter regions in proximity to the ventral aspect of the internal capsule.

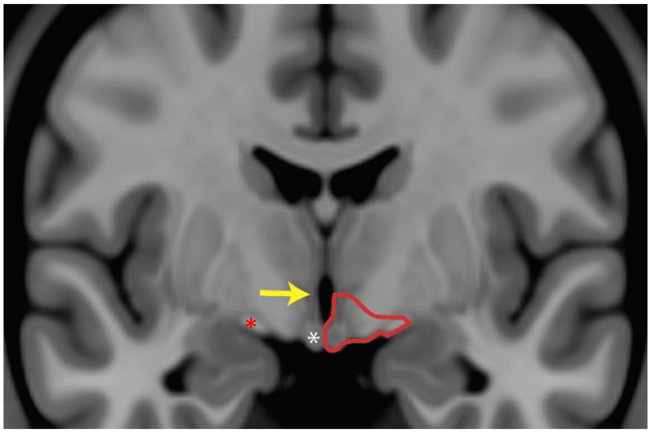

When the mammillary body ends, the hypothalamus has ended. Thus, the anterior VDC (aVDC) is also considered to end and is replaced by the posterior VDC (pVDC) ROI. In the pVDC, the trajectory of the superior margin is adjusted to be more horizontal and thereby enclose gray matter structures at the inferior margin of the internal capsule. This trajectory ends laterally by enclosing the optic tract (red asterisk).

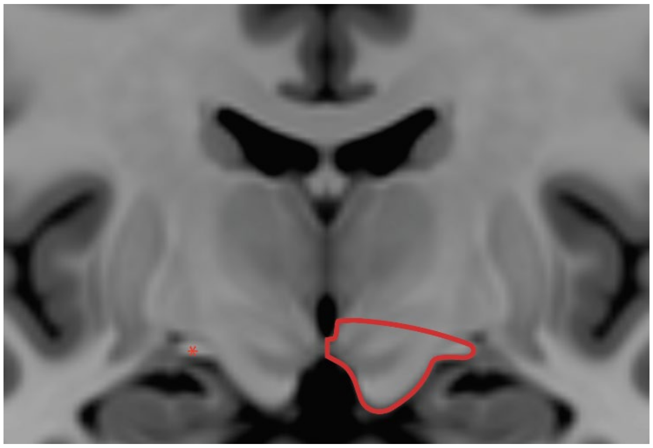

In more posterior sections, the superior pVDC border will be a slightly curved line that extends from the hypothalamic sulcus to the lateral aspect of the optic tract. The inferior aspect of the pVDC will border the subarachnoid space until the brainstem connects, as indicated in the image to the right. Since the brainstem is already segmented, the inferior border of the pVDC directly adjoins it.

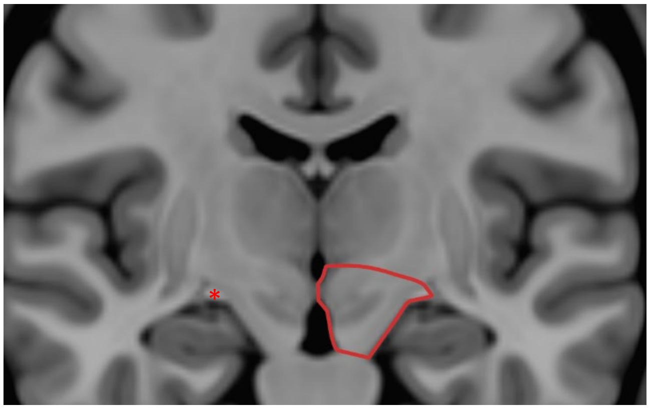

Continue to segment the pVDC in more posterior sections and note that the optic tract (red asterisk) may be more difficult to visualize as the lateral geniculate nucleus (LGN) begins to appear. Use the segmentation from previous coronal sections to confirm the location of the optic tract moving more posteriorly.

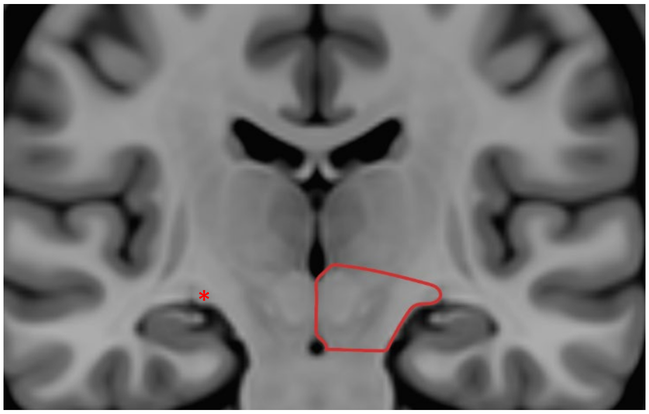

In subsequent sections, the optic tract will be replaced by the LGN, a gray matter structure (see image to right). The lateral aspect of the LGN now comprises the lateral border of the pVDC. In addition, when the thalami separate to allow the TCF and the third ventricle to be in apposition, the habenula will appear as whitish bumps medially. Segment the habenula at the medial aspect of the pVDC as shown in the image to the right.

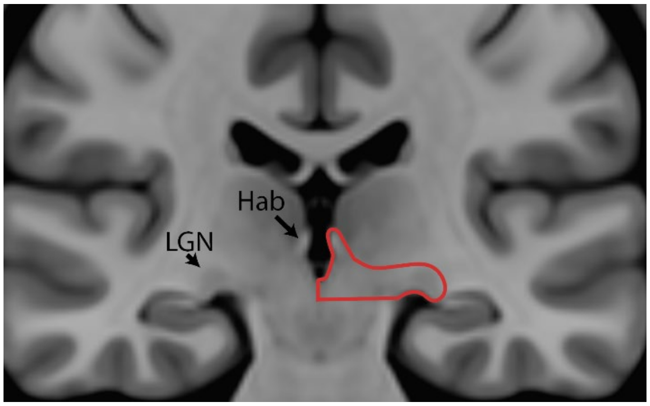

More posteriorly, the habenula will disappear, and the LGN will diminish in size. A gray matter bump will appear between the LGN and the brainstem. This bump is the medial geniculate nucleus (MGN, white asterisk), which should be segmented with the pVDC. Note that the inferior and medial borders of the pVDC will adjoin the brainstem, and the superior border will adjoin the thalamus. Confirm the LGN and MGN are completely within the pVDC segmentation, and not in the prior thalamic segmentation. When the MGN disappears, the pVDC is no longer present.

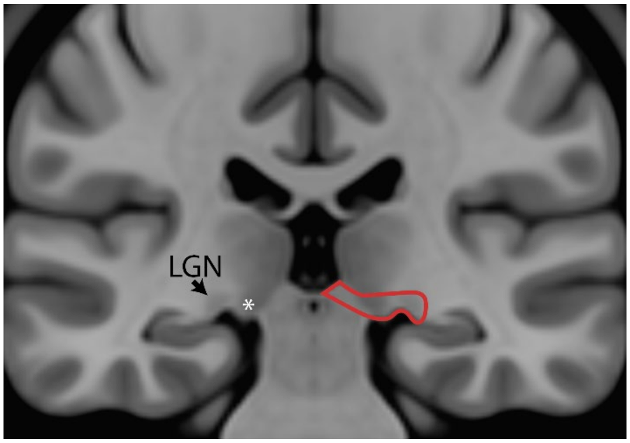

Review the aVDC and pVDC segmentations on axial and sagittal views.

The Amygdala

The amygdala is located in the medial temporal lobe. It has an ovoid, almond-like shape and is situated anteriorly and superiorly to an adjacent gray matter structure called the hippocampal formation. Anteriorly, the amygdala borders the anterior portion of the temporal cortex. Superiorly, the amygdala is bordered by the basal portion of the frontal lobe. Medially, the amygdala borders the temporal cortex as well as the subarachnoid space adjoining the medial surface of the temporal lobe. The lateral borders of the amygdala are the inferior horn of the lateral ventricle and, more rostrally, the white matter of the anterior temporal lobe. Inferiorly, the amygdala is bordered by the ventral cortex of the temporal lobe and, more posteriorly, the hippocampal formation.

The amygdala is a relatively small structure, but one of the more difficult to segment. The following text is divided into two parts. The first part will detail how to use the ***Guide Markup*** tool in the NeuroSegmentation module to generate anatomically-defined reliable borders. The second part will show how to use these borders in conjunction with other anatomical landmarks to segment the amygdala in coronal sections.

A three-dimensional image of the amygdala (blue) overlaid on a sagittal section shows that the amygdala is large anteriorly and tapers more posteriorly. The image also shows the relationship of the amygdala to the hippocampal formation (HF), a relationship that will be important in the segmentation of both structures. This relationship can be conceived of as the amygdala being a ball kicked toward the front of the brain by a foot represented by the hippocampal formation. The anterior part of the ball (amygdala) is in front of the foot, while the posterior part of the ball is above the top of the foot (hippocampal formation).

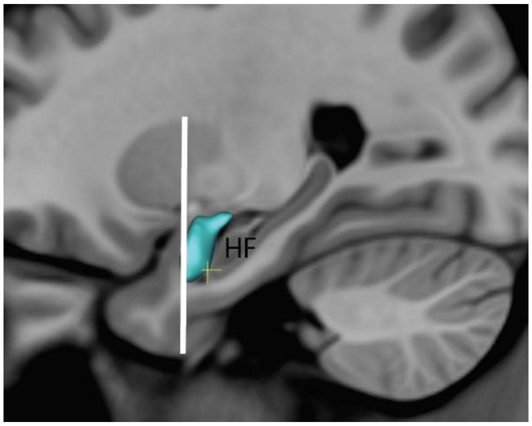

The anterior portion of the amygdala is difficult to visualize in the coronal view, but this plane is the anatomical plane of brain segmentation. Similarly, the border between the amygdala and hippocampal formation is difficult to discern in some brains in coronal sections. Due to these difficulties, both the sagittal and axial views are critical in outlining the shape of the amygdala prior to segmentation. The ***Guide Markup*** tool will be used in the sagittal and axial views to approximate the borders of the amygdala. The transects of these borders will be observed in the coronal view and are used to assist segmentation.

First identify a sagittal section like that observed in the image to the right where the amygdala (A) and hippocampal formation (HF) are clearly distinguished from each other.

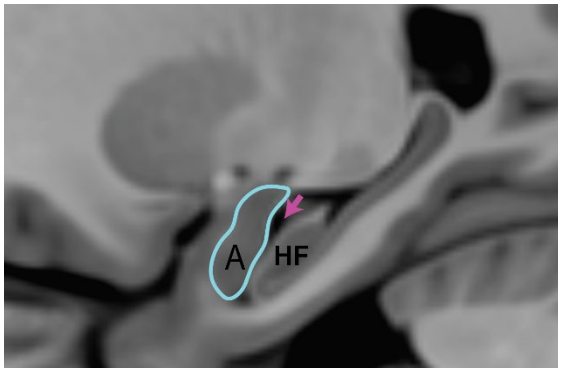

Next, select a ***Guide Line*** in the NeuroSegmentation module. Make sure that the ***Line*** checkbox is selected for the sagittal and axial views, but not for the coronal view, and select the ***Intersection*** checkbox for the coronal view only. This will allow lines to be drawn on the sagittal and axial views, and the transects of these lines to be visualized in the coronal view. Next, draw a line (yellow, right image) between the amygdala anteriorly and the more posterior hippocampal formation.

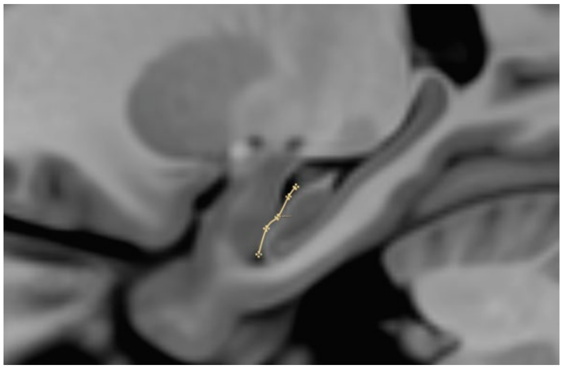

After drawing the ***Guide Line***, examine a coronal section similar to that seen on the right. The yellow dot represents the transect of the guide line drawn on the sagittal view. Notice that this dot is located at the border between the amygdala superiorly and the hippocampal formation inferiorly. Return to the sagittal viewer, and in different sections draw ***Guide Lines*** between the amygdala and hippocampal formation.

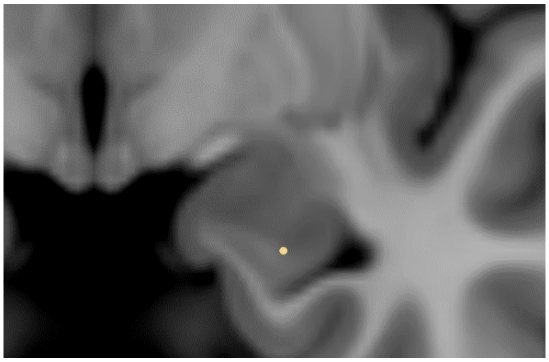

By drawing several lines between the amygdala and hippocampal formation in different sagittal sections, a border between the amygdala and the hippocampal formation can now be seen in the coronal view. This approach is valuable in brains where the border is difficult to ascertain. The section on the right shows a coronal section in which multiple transects are illustrated by different colored dots. Together these

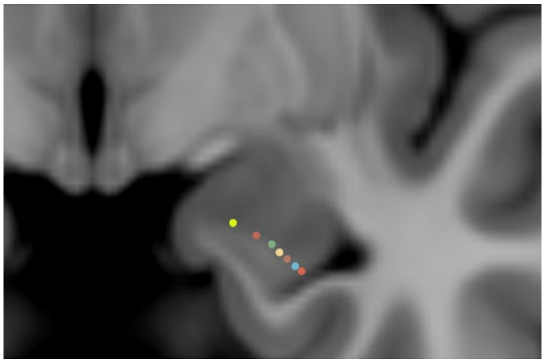

dots represent the border between the amygdala superiorly and the hippocampal formation inferiorly.

This procedure is also useful for determining the anterior border of the amygdala. ***Guide Lines*** (green, right image below) are drawn in successive sagittal sections to delimit the anterior portion of the amygdala. The coronal section on the left corresponds to the vertical crosshair line on the sagittal section in the right image below. The transects through the upper curve and lower curve drawn on the sagittal section are represented as dots on the coronal section on the left. These transects represent the borders of the amygdala.

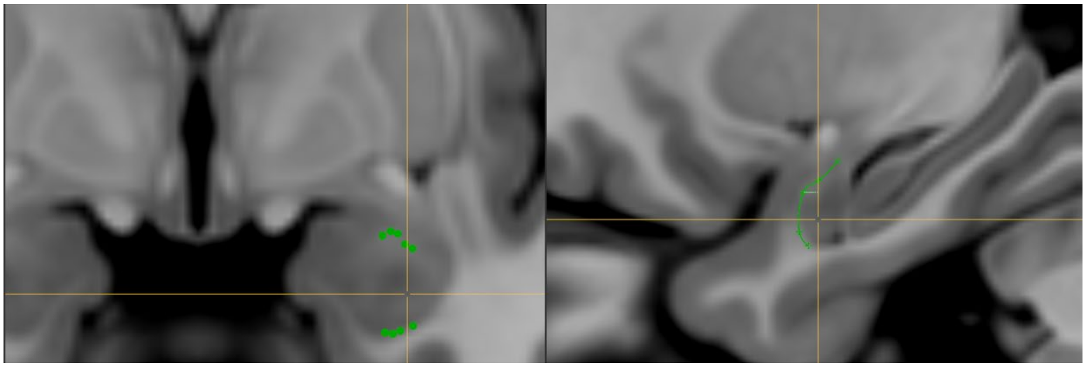

More anteriorly, this approach shows the first coronal section of the amygdala, and indicates the approximate borders of this region. The image below shows a sagittal section on the right with a green ***Guide Line*** curve to delineate the anterior amygdalar border. The vertical line of the crosshair in the sagittal view (right, below) shows the location of the coronal section illustrated on the left. The transects (dots) shown in this coronal section are the results of several guide lines that together approximate the anatomical borders of the anterior most section of the amygdala.

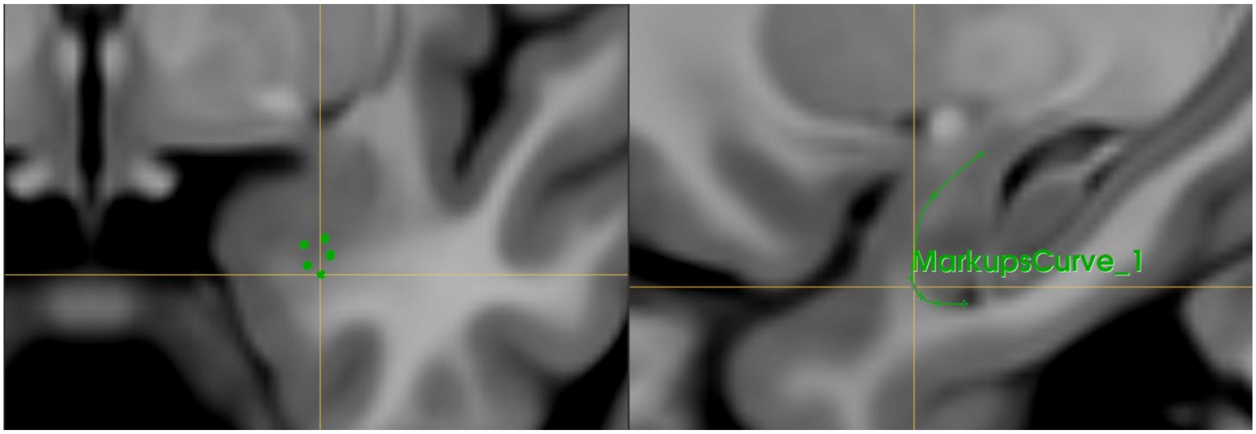

Guide Lines should also be drawn on the axial view (left on image below) to produce borders in the coronal view (right image below).

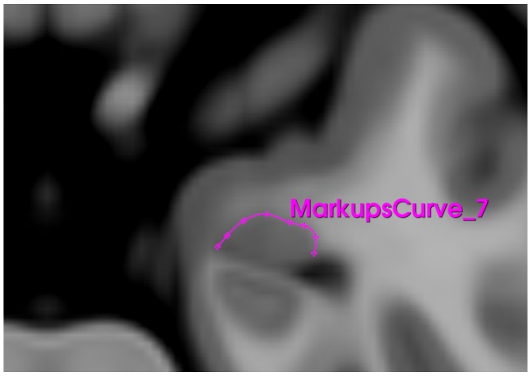

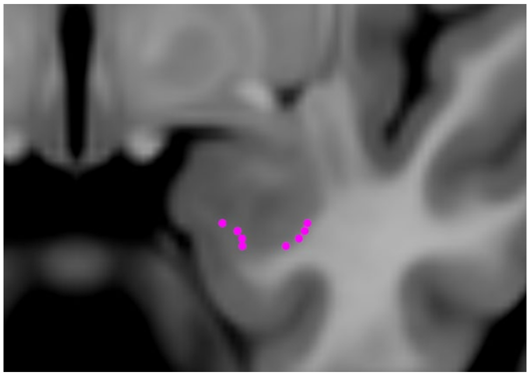

It is important to note that the above illustrations are from an averaged brain where the borders can be discerned. The ***Guide Line*** tools was developed for use in MRI scans where the borders may not be as distinct, and the utility of the tool is best appreciated when applied to those brains.

Once the anterior amygdalar border as well as the border between the amygdala and the hippocampal formation have been demarcated, turn to the coronal views to segment the amygdala.

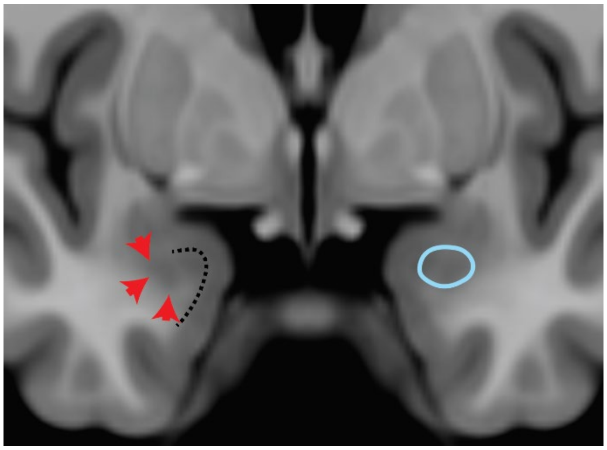

The anterior portion of the amygdala is defined by the transects of the previously drawn ***Guide Lines***. In the anterior-most section of the amygdala, the amygdala is operationally defined as being positioned below the cortical surface (red arrowheads under black dotted line in image to right), and is segmented similar to the blue outline in the image to the right.

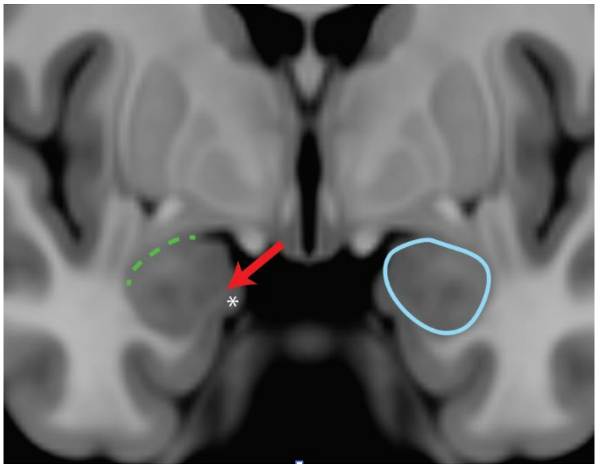

In subsequent sections, the amygdala extends dorsomedially to the exterior of the brain. The lateral and inferior parts of the amygdala are bordered by white matter. The dorsolateral border extends from the lateral invagination of the cerebral exterior to the lateral margin of the amygdala (dotted green line in image to right). Also notice the finger of white matter inferior to the amygdala that serves as its medial and inferior border (tip of red arrow) at this level of the uncus (white asterisk). This white matter separates the amygdala from the anterior part of the cerebral

cortex medially. Use the ***Threshold*** tool to obtain a border between the external subarachnoid space and the amygdala. Finally, draw the borders of the amygdala based on the position of the ***Guide Line*** transects.

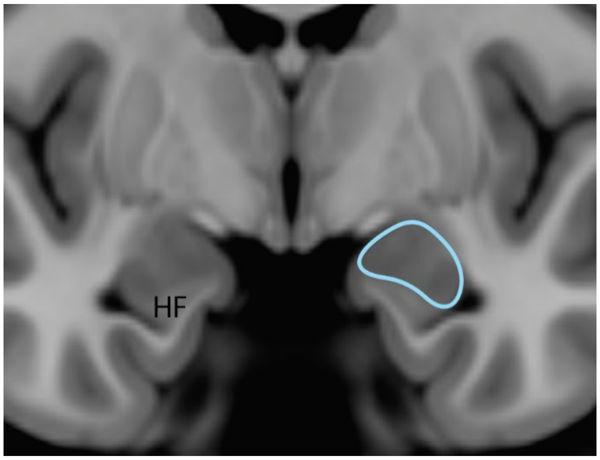

More posteriorly, a structure with slightly more intense voxels appears inferior to the amygdala. This is the hippocampal formation (HF). The size of the hippocampal formation will increase in more posterior coronal sections, and the size of the amygdala will diminish. The border between the two structures was defined previously using ***Guide Line*** transects. The approximate position and extent of the amygdalar segmentation at this coronal level is indicated by the blue outline in the image to the right.

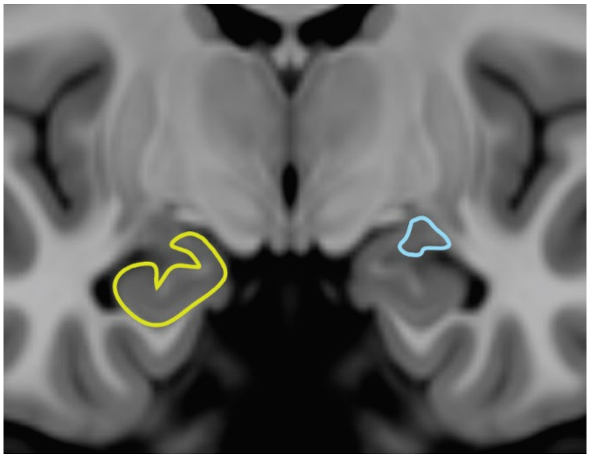

In more posterior sections, the hippocampal formation will adopt a rough 'W' shape (yellow outline) and the amygdala will come to lie lateral to and above the medial arm of the W (blue outline).

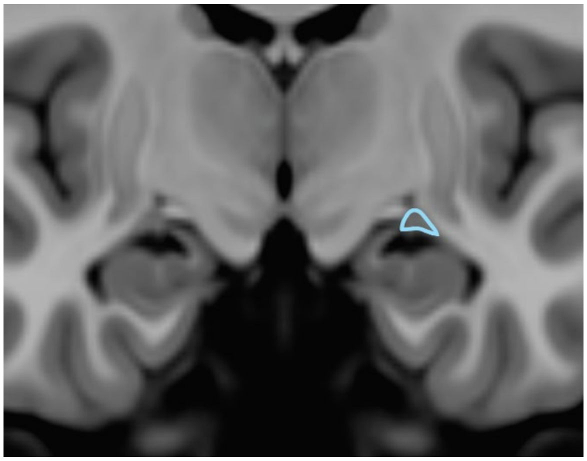

The amygdala will continue to shrink in this position until it disappears (blue outline).

Review the results of the amygdalar segmentation on the 3D view as well as on axial, sagittal and coronal series and edit if necessary.

The Hippocampal Formation

The hippocampal formation (HF) is a multicomponent cortical region that includes a number of subfields (CA1, CA2, CA3, CA4, CA4*, dentate gyrus), white matter components (alveus, portions of the fornix), as well as discrete cortical regions (e.g., subiculum, parasubiculum) that connect the hippocampal formation to the temporal lobe neocortex. It should be noted that in previous versions of this segmentation approach, this ROI was referred to simply as the hippocampus (e.g., Filipek et al., 1994; Makris et al., 1999; Seidman et al., 1999, 2002). This term has been replaced in the present study with the more precise term hippocampal formation to align with its currently accepted ontology.

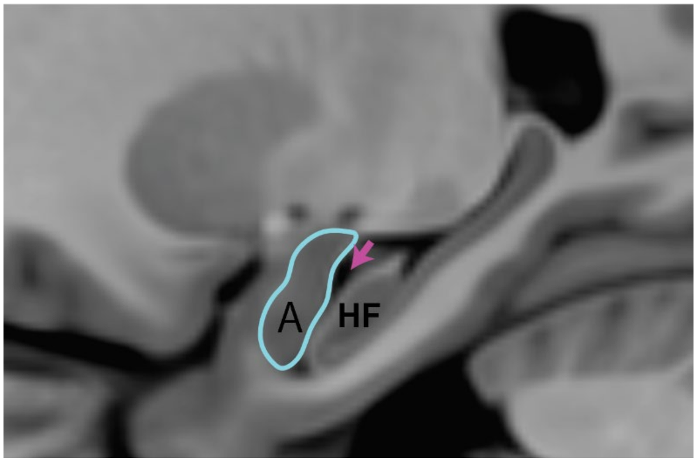

The hippocampal formation is a curved cortical structure located and folded under the medial temporal lobe. Its antero-posterior extent is visualized in the sagittal plane (right). In this plane, the hippocampal formation (HF) is adjacent to the inferior horn of the lateral ventricle (purple arrow). The amygdala (A) borders the hippocampal formation anteriorly. The medial border of the hippocampal formation is the medial curved surface of the hemisphere, which adjoins the subarachnoid space exterior to the brain. Inferiorly and laterally, the hippocampal formation is bordered by the white matter of the temporal lobe. More posteriorly, the hippocampal formation rises to be inferior and then posterior to the thalamus. Guide lines have been drawn previously to specify the border between the amygdala and the hippocampal formation, as part of the amgydalar segmentation procedure (see above). These lines define the anterior border of the hippocampal formation, which appears as a sliver of higher intensity voxels between the amygdala (A) and the inferior horn of the lateral ventricle.

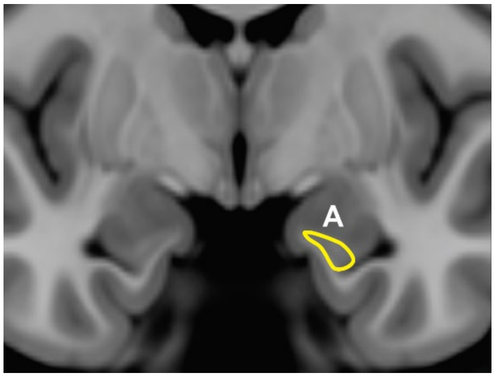

Identify the anterior most section of the hippocampal formation. In this section, the hippocampal formation appears as a small sliver similar to that shown in the image to the right (yellow outline). Segment the hippocampal formation with the ***Draw*** tool, and use the ***Threshold*** tool to specify the border between the hippocampal formation and the inferior horn of the lateral ventricle. A separate threshold should be used to define the border between the hippocampal formation and the white matter of the temporal lobe.

From this point posteriorly, the hippocampal formation volume will increase while that of the amygdala diminishes. The hippocampal formation will change shape, often appearing as a 'W' (yellow outline in image to right). The medial arm of the hippocampal formation curves around and ends (tip of white arrow). This point is the division between the hippocampal formation and the amygdala, and may require a slight adjustment in the brightness/contrast to optimally visualize.

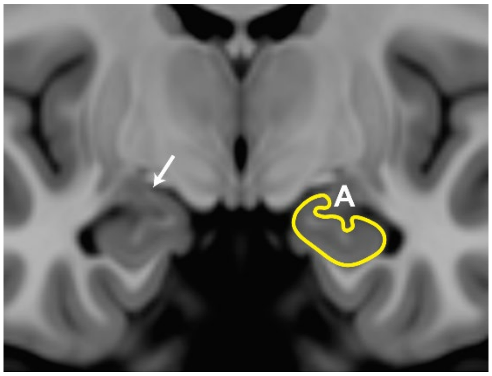

In more posterior sections, the 'W' shape becomes less distinct, and the amygdala and the hippocampal formation frequently become separated by a small space (tip of the white arrow). Also note that the hippocampal formation bulges into the inferior horn of the lateral ventricle and often is perceived to touch the lateral wall, thereby separating the inferior horn into two components.

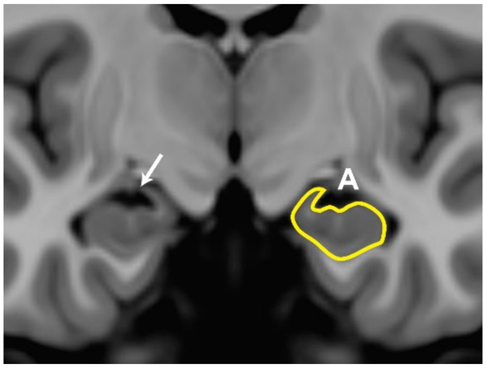

When the amygdala disappears, the hippocampal formation will lose its medial tip and coalesce into a more compact shape. The connection between the hippocampal formation and the adjacent temporal cortex medially is now visible, and the lateral border of the hippocampal formation should be specified at the bend of the cortex (yellow dashed line) as a diagonal line (approximately at a 45-degree angle) cutting through the cortex to reach the hemispheric surface. The other feature of note at this level is the coalescence of the thin white matter capsule into a fiber tract on the dorsal and medial aspect of the hippocampal formation (tip of white arrow). This white matter will be segmented at this stage as part of the hippocampal formation.

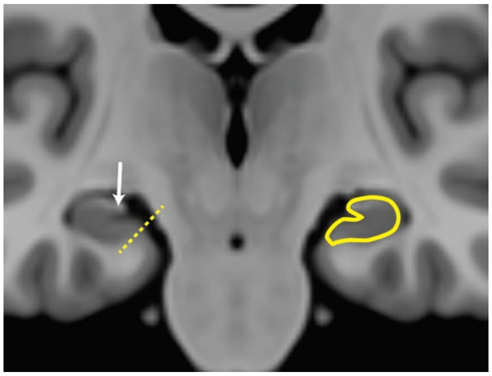

Continue including this medial white matter bundle (black arrow) in the hippocampal formation segmentation up to the level the posterior commissure (white asterisk). In the next posterior section, no longer include this white matter bundle in the hippocampal formation segmentation.

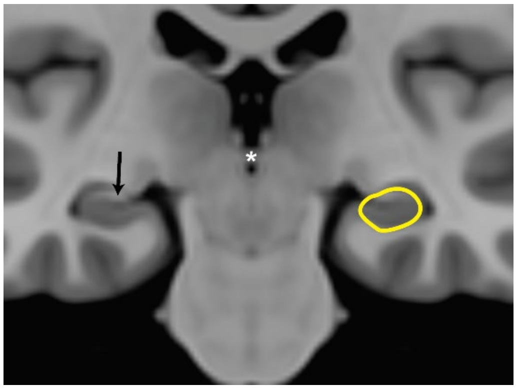

More posteriorly, the hippocampal formation (yellow outline) continues, while the thalamus (T) diminishes in volume. At this level, the white matter previously segemented as part of the hippocampal formation becomes the fornix (F). The fornix is located superior to the hippocampal formation and wraps along the lateral aspect of the thalamus.

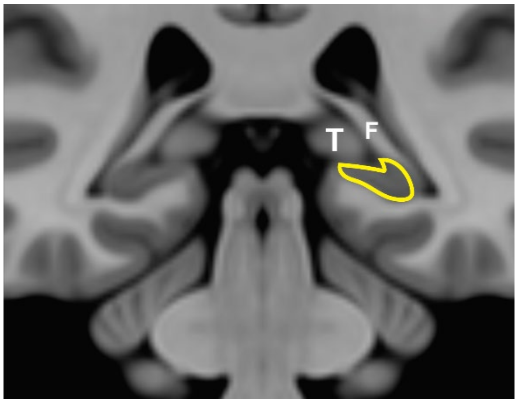

The hippocampal formation adopts the position of the thalamus after the latter nucleus end. The hippocampal formation is thus located inferior to the corpus callosum. In more posterior sections, the hippocampal formation ends.

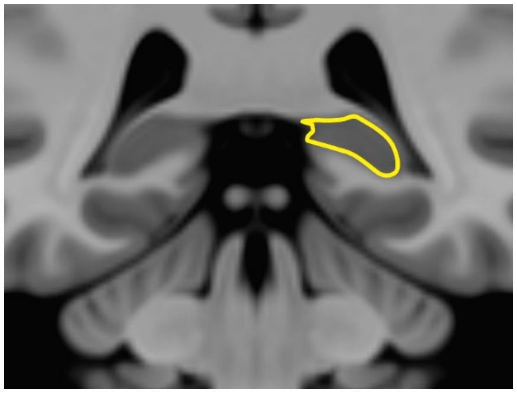

Review the hippocampal formation segmentation on the 3D, axial and sagittal series.

Inferior Horn of the Lateral Ventricle

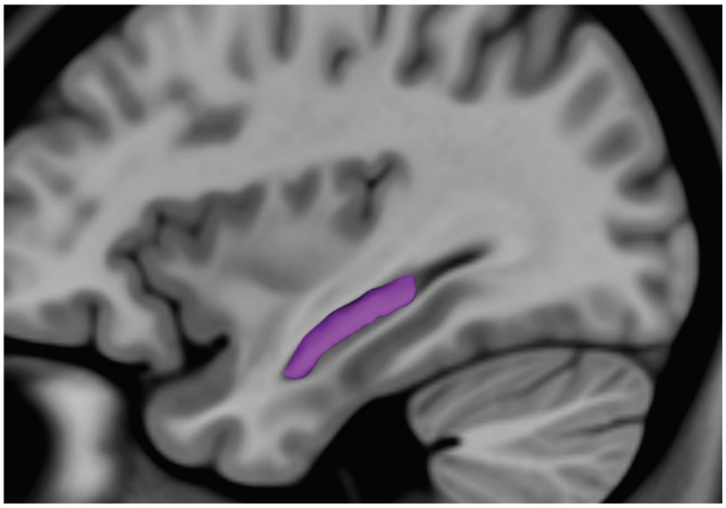

The inferior horn of the lateral ventricle (ILV) is the part of the lateral ventricle that extends into the temporal lobe. It is bordered by the hippocampal formation, amygdala, and white matter, and these structures often appear to compress the inferior horn. Thus, the ILV varies significantly in shape and the CMA guidelines do not consider this to be a reliably segmented structure. Nonetheless, it is an important structure given its topographic relationship with the hippocampal formation and amygdala.

The ILV should be segmented according to the following guidelines.

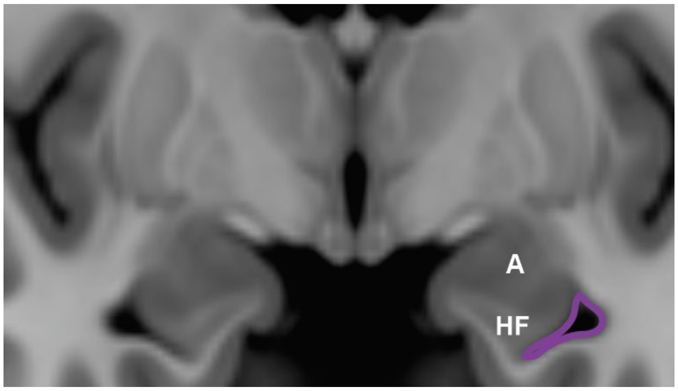

Begin the segmentation of the ILV at the anterior portion of the hippocampal formation (HF) and the lateral portion of the amygdala (A). Use the ***Threshold*** tool with gray and white matter separately to delineate the borders of the ILV. Be sure to move anteriorly to see the anterior end of the ILV, since in some instances, it will extend to the inferior margin of the amygdala. The anterior ILV may also be disconnected from the rest of the ILV in some brains.

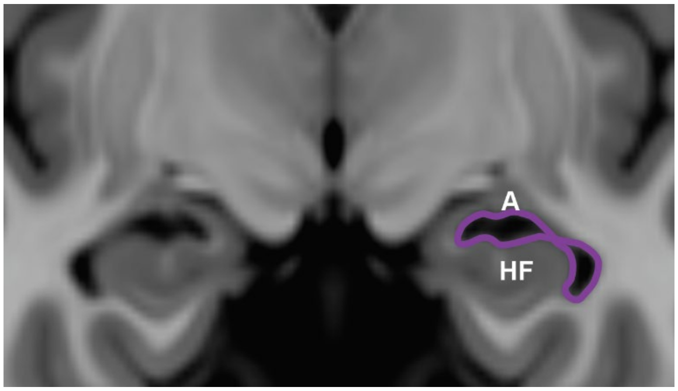

In more posterior sections, the ILV extends between the hippocampal formation (HF) inferiorly and the posterior portion of the amygdala (A) superiorly. Notice that the hippocampal formation bulges into the ILV to reach its supero-lateral wall.

After the amygdala ends, the ILV will cup the superior aspect of the hippocampal formation. On the medial aspect of the hippocampal formation, at the location where the white matter coalesces (yellow arrow), there is a thin membrane called the tela choroidea that separates the ILV from the subarachnoid space. This membrane is shown in the inset (arrow on left, dotted line on right) after altering the brightness for better visualization. If the membrane is not visible, use the medial and superior aspect of the white matter as the medial ILV border (tip of yellow arrow in larger image).

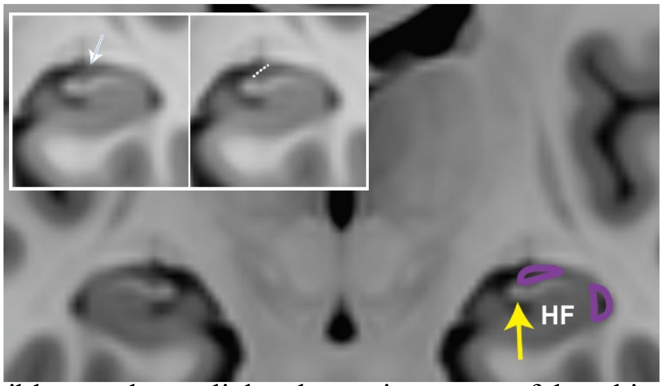

More posteriorly, the curve of the ILV will change to point to the enlarging lateral ventricle (LV). The hippocampal formation (HF) continues to form the medial border of the ILV. The last coronal section of the ILV is that in which the lateral ventricle and ILV are separate entities.

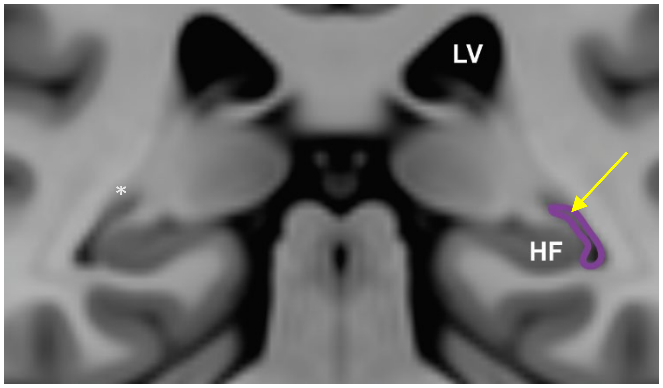

During ILV segmentation, keep in mind that the ventricular space may be filled with choroid plexus, which should be included in the segmentation (tip of yellow arrow). Turn off masking to do so. Be sure to not confuse the choroid with the tail of the caudate nucleus (white asterisk, left side of image), which appears superior to the ILV space.

When the lateral ventricle (LV, purple outline) and ILV spaces are continuous and connected, the ILV is not present and the entire space is considered as the lateral ventricle segmentation, as described previously.

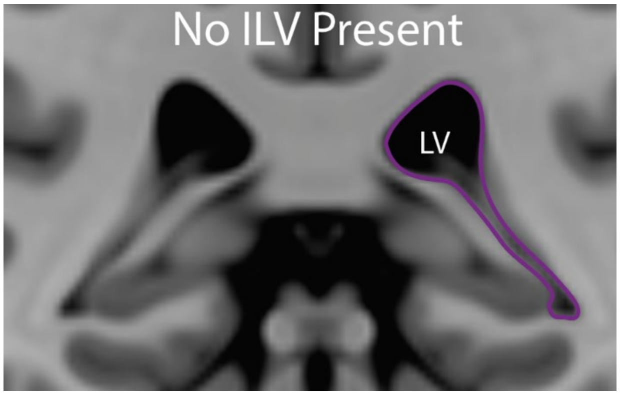

Review the ILV segmentation on sagittal, axial and 3D views.

The Optic Chiasm

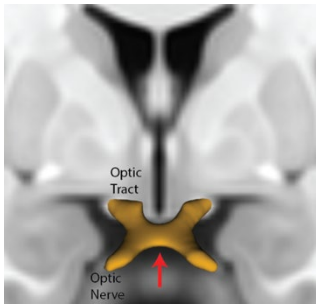

The optic chiasm is a partial crossing of the optic nerves from the eyes. The image to the right shows the optic chiasm and associated structures in 3D overlaid on a coronal image. The optic nerve originates from the back of the eye and courses posteriorly under the forebrain. The chiasm (red arrow) represents a partial crossing of the optic nerves (right image). Posterior to the chiasm (red arrow), two fiber tracts (by convention termed optic tracts not optic nerves) emanate and course posteriorly into the brain. The tracts, the nerves, and the chiasm together form the shape that gives the chiasm its name ('chiasm' means shaped like the letter X).

According to CMA guidelines, the optic chiasm is first segmented in the coronal section in which the third ventricle initially appears anteriorly (see below, dotted white line to left). Select a threshold between the subarachnoid space and the white matter of the optic chiasm to produce a segmentation outline like that observed in the image below (orange, right).

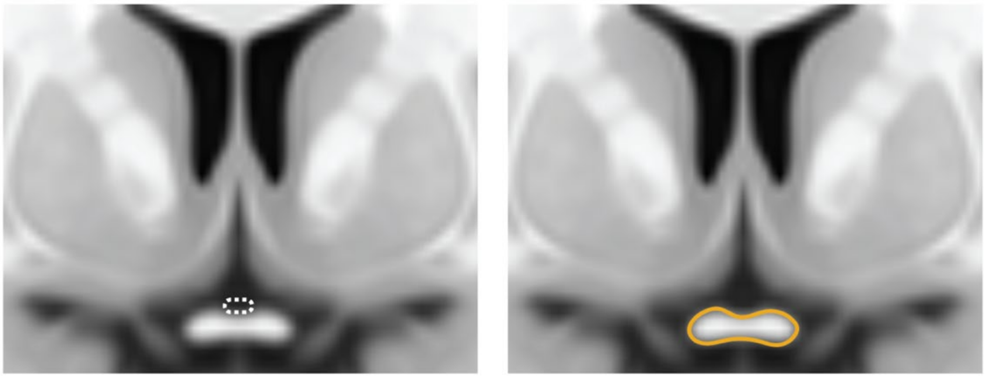

Continue segmenting the optic chiasm in more posterior sections. When the optic chiasm separates into two distinct tracts (image below), no longer segment the optic chiasm.

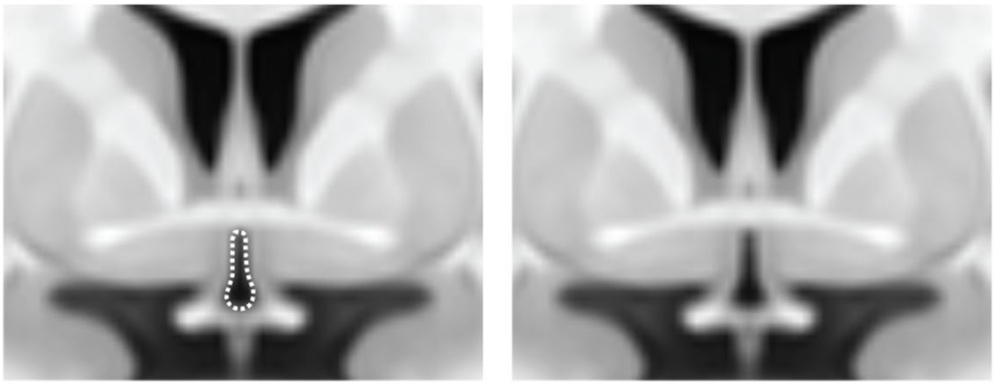

Review the segmentation on axial and sagittal sections. Make sure that any unlabeled voxels between the third ventricle and the optic chiasm are ascribed to one of the segmentations.

The Fifth Ventricle

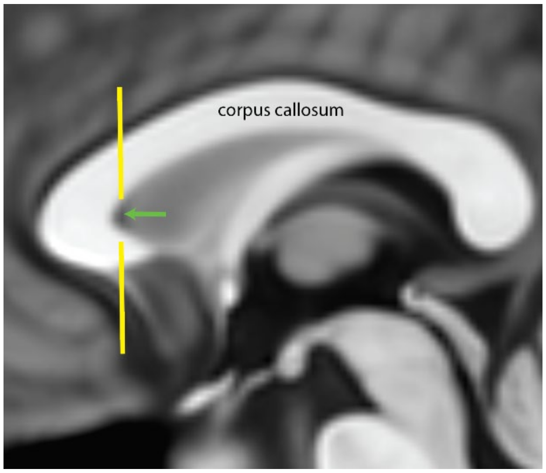

The fifth ventricle, also known as the cavum septum pellucidum, is a CSF-filled space that exists between the two lateral ventricles in the anterior part of the brain. Note that this structure is a single entity on the midline of the brain that does not have distinct left and right hemisphere components. On sagittal sections close to the midline, the fifth ventricle (tip of the green arrow) may be observed on the inner aspect of the anterior bend of the corpus callosum. A coronal section through the fifth ventricle taken at the position of the yellow lines produces an image similar to that shown below which depicts the fifth ventricle in the coronal plane.

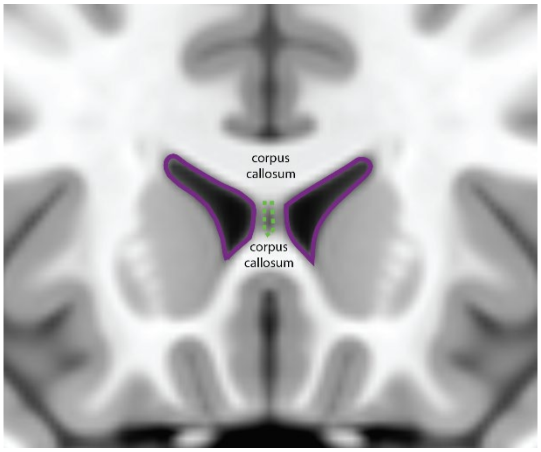

At this level, the corpus callosum is observed in two locations, between which are the lateral ventricles (purple). Each ventricle is bordered medially by a thin membrane called the septum pellucidum. Between the left and right septa pellucida is the fifth ventricle (green dashed line), which is also bordered superiorly and inferiorly by the corpus callosum. In most brains, the fifth ventricle is typically small and is present in only a handful of sections. However, the fifth ventricle can enlarge following conditions such as trauma that create small tears in the septum pellucidum and allow CSF to enter and expand the fifth ventricle.

Segment the fifth ventricle using the ***Threshold*** tool. Given the small size of the fifth ventricle, this tool may be of limited utility; in these cases, use anatomical judgment to outline the borders. Begin your segmentation at the coronal section in which the fifth ventricle is largest. Move to more anterior and then to more posterior sections until the segmentation is complete.

Review the segmentation of the fifth ventricle on axial and sagittal series.

Glossary of Terms:

Sagittal Plane: An imaging plane that divides the brain into left and right halves. The midsagittal plane is exactly mid-way between the hemispheres of the brain.

Coronal Plane: An imaging plane named for the coronal suture of the skull. This plane is parallel to the long axis of the body, and perpendicular to the sagittal and axial planes.

Axial Plane: An imaging plane perpendicular to the long axis of the body.

Alveus: A layer of hippocampal axons adjoining the inferior horn of the lateral ventricle.

Anterior commissure: A prominent fiber tract crossing the midline and located inferior to the columns of the fornix. This commissure mainly connects regions of the left and right temporal lobe.

Amygdala: The amygdala is an almond-shaped collection of subcortical nuclei in the anterior portion of the temporal lobe. The amygdala is involved in sensory-sensory associations related to emotion (especially fear and aggressive behavior), including sensory-visceral associations.

Arachnoid: The spider-like membrane that covers the brain. The arachnoid is the middle of three membranes, together called meninges, that cover and protect the brain inside the skull. The subarachnoid space is between the arachnoid membrane and the more interior pia.

Basal Ganglia: A group of interconnected subcortical nuclei. The basal ganglia comprise the caudate nucleus, the putamen, the nucleus accumbens, the globus pallidus, the subthalamic nucleus, and the substantia nigra.

Brainstem: The brainstem is a region of the neuraxis between the diencephalon and the spinal cord. It is traditionally divided into three parts: midbrain, pons, and medulla. The brainstem contains fiber tracts connecting the spinal cord with the forebrain, nuclei and tracts associated with cranial nerves, as well as long-range neurotransmitter nuclei and systems controlling sleep, wake, and vigilance.

Caudate Nucleus: The caudate nucleus is a comma-shaped nucleus that forms the lateral border of the lateral ventricle. It contains a large anterior head, an intermediate-sized body, a posterior bend, and a slender tail. The caudate nucleus is one of the basal ganglia. It is connected most heavily with association areas of the cerebral cortex and involved with circuits modulating cognitive and motor function.

Cerebellum: A large convolutional structure located in the brainstem region. It is separated from the pons of the brainstem by the fourth ventricle. The cerebellum is involved in the sequencing and modulation of movement as well as cognitive and emotional function.

Cerebellar Peduncles: Three white matter formations that connect the cerebellum with spinal cord, brainstem and forebrain regions.

Cerebral Aqueduct: A small channel containing cerebrospinal fluid (CSF) that connects the third and fourth ventricles. The aqueduct is located in the midbrain component of the brainstem and is included within the fourth ventricle ROI for the purpose of segmentation.

Cerebral Peduncle: A large fiber bundle on the ventral surface of the midbrain comprising axons emanating from the cerebral cortex and terminating in regions of the brainstem and spinal cord.

Cerebrospinal Fluid: The cerebrospinal fluid (CSF) is a clear secretion of the choroid plexus. It is derived from blood, and fills the brain's ventricular system (lateral ventricles, third ventricle, fourth ventricle) as well as the subarachnoid space surrounding the brain.

Choroid Plexus: The choroid plexus is the highly vascular organ that secretes cerebrospinal fluid. The choroid plexus is found in parts of the lateral ventricles, the third ventricle, and the fourth ventricle.

Claustrum: The claustrum is a thin sheet of gray matter lateral to the putamen. It has connections with widespread regions of the cerebral cortex, but little is known about its function.

Cornu Ammonis: A term used to refer to numbered subdivisions of the hippocampus. Cornu Ammonis is often abbreviated as CA, with a numerical suffix indicating the specific subfield (e.g., CA1).

Diencephalon: The second division of the neuraxis that includes the thalamus, the hypothalamus, the habenular nuclei, and the subthalamic nuclei.

Dentate Gyrus: A subfield of the hippocampal formation resembling a tooth.

Dura: The outermost fibrous covering of the brain. The dura is one of three membranes, together called meninges, that cover and protect the brain inside the skull.

External Capsule: A thin white matter pathway between the putamen and the claustrum.

Fifth Ventricle (also called the cavum septum pellucidum): A space filled with cerebrospinal fluid that is not directly connected with the cerebral ventricular system. This space exists posterior to the anterior bend (genu) of the corpus callosum and is bordered by the paired septum pellicuda laterally.

Foramen of Monro: The canal between each lateral ventricle and the third ventricle.

Fornix (pl: Fornices): A fiber bundle containing axons from cells in the hippocampus and terminating principally in the mamillary bodies of the hypothalamus. The fornix has an appearance of an arch with several portions: 1) the *fimbria* (fringe) refers to the region of the fornix where axons coalesce, 2) the *crus* (legs) are the initial parts of the fornix that form the posterior bend of the arch, 3) the body, the region of the fornix that forms the medial and inferior border of the lateral ventricle, 4) the columns, the anterior bend of the arch that courses inferiorly to terminate in the hypothalamus.

Fourth Ventricle: A tent-shaped ventricular space between the brainstem and the cerebellum. The fourth ventricle communicates with the third ventricle via the cerebral aqueduct. The fourth ventricle also communicates with the subarachnoid space surrounding the brain via two lateral apertures and a median aperture. The fourth ventricle, according to CMA guidelines, includes the cerebral aqueduct.

Globus Pallidus: One of the nuclei of the basal ganglia located medial to the putamen and lateral to the internal capsule. It has two parts – a pars internus and a pars externus. These divisions have different connections and functions, but are often difficult to distinguish on T1w MRI images. The globus pallidus is the output nucleus of the basal ganglia, and its projections terminate in the subthalamic nucleus and the thalamus.

Habenular nuclei (pl: habenulae): Nuclei that line part of the medial aspect of the posterior thalamus. The habenular nuclei receive input from forebrain nuclei and relay it to the brainstem.

Hippocampal Formation: A region of the cerebral cortex curved under the margin of the temporal lobe medially. The hippocampal formation is involved in spatial navigation and memory consolidation, and

consists of several interconnected regions: the subiculum, the cornu ammonis subfields, and the dentate gyrus. A main white matter projection of the hippocampal formation is the fornix, which projects to the mammillary bodies of the hypothalamus.

Hypothalamic Sulcus (of Monro): An invagination in the lateral wall of the third ventricle that separates the thalamus superiorly from the hypothalamus and brainstem inferiorly.

Hypothalamus: A region of the diencephalon inferior to the hypothalamic sulcus. The hypothalamus is involved in species-specific behaviors as well as control of visceral, autonomic and homeostatic function. It contains many nuclei, including the mammillary bodies.

Inferior Horn of Lateral Ventricle: A region of the lateral ventricle located within the temporal lobe.

Internal Capsule: A roughly V-shaped white matter bundle containing projections from the cerebral cortex that terminate in diencephalic, brainstem and spinal cord nuclei. The internal capsule has two principal parts: the anterior portion between the caudate nucleus and the putamen/globus pallidus, and the posterior portion between the thalamus and putamen/globus pallidus.

Inferior Colliculus: Paired nuclei that appear as little hills (colliculus (Latin): little hill) on the superior aspect of the midbrain involved in auditory processing. The inferior and superior colliculi together comprise the midbrain tectum.

Lateral Geniculate Nucleus: A thalamic nucleus located posterior and inferiorly to the main portion of the thalamus. The axons of each optic tract terminate within the lateral geniculate nucleus and relay visual signals from the contralateral visual hemifield through both eyes.

Lateral Ventricle: A roughly C-shaped space within each hemisphere filled with cerebrospinal fluid (CSF). Each lateral ventricle has different parts: the anterior horn within the frontal lobe, the body within the frontal and parietal lobes, the inferior horn within the temporal lobe, and the posterior horn within the occipital lobe. The atrium (or trigone) of the ventricle is the confluence of the body, inferior and posterior horns. Choroid plexus is present within the inferior horn, the atrium and the body of the lateral ventricle.

Lenticular Nucleus: A nucleus with the shape of a lens, comprising the globus pallidus and putamen.

Mammillary Bodies: Paired circular nuclei of the hypothalamus that receive projections chiefly from the ipsilateral hippocampal formation via the fornix. The mammillary bodies are located in the inferior and posterior portion of the diencephalon.

Medial Geniculate Nucleus of the Thalamus: located posterior and inferiorly to the main portion of the thalamus, and lateral to the superior colliculus of the midbrain. The medial geniculate nucleus receives auditory information and relays these signals to auditory regions of the cerebral cortex.

Medulla (medulla oblongata): The region of the brainstem between the pons and the spinal cord.

Meninges: The three coverings of the brain, including the outer dura, middle arachnoid and inner pia. The meninges are located between the skull and the brain.

Midbrain: The upper region of the brainstem located between the pons and the diencephalon. It contains the tectum (superior and inferior colliculi) posteriorly, and the cerebral peduncles anteriorly.

Middle Cerebellar Peduncle: A large fiber bundle connecting the pons of the brainstem with the cerebellum.

Nucleus Accumbens: A region of the basal ganglia that connects the caudate nucleus and the putamen. The nucleus accumbens is often referred to as the ventral striatum and is involved in reward and reinforcement.

Obex: The inferior notch of the fourth ventricle.

Optic chiasm: A midline structure ventral to the hypothalamus that is a partial crossing of the optic nerves from each retina to the opposite hemisphere. The fiber bundles that emerge posteriorly from the optic chiasm are termed the optic tracts.

Optic tracts: Fiber bundles emanating from the optic chiasms that convey visual signals from the contralateral visual hemifield of both eyes to the lateral geniculate nucleus of the thalamus.

Parasubiculum: A region of the hippocampal formation.

Pia: The innermost of the three membranes covering of the brain. The pia is a thin layer on the surface of the brain interior to the arachnoid membrane. The subarachnoid space is between the pia and the arachnoid membrane.

Pretectum: A region of nuclei located between the superior colliculus and the thalamus.

Pons: The region of the brainstem between the midbrain and the medulla. It is connected to the cerebellum via the middle cerebellar peduncle.

Putamen: A nucleus of the basal ganglia lateral to the globus pallidus principally involved in motor function. The putamen is separated from the claustrum laterally by the external capsule.

Pyramidal decussation: The region of the anterior medulla where motor axons from the cerebral cortex undergo a partial crossing before entering the spinal cord.

Septum Pellucidum: A thin membrane that forms the medial border of the lateral ventricle. These left and right membranes are often fused on the midline.

Spinal Cord: The region of the central nervous system located in the vertebral canal.

Striatum: A collection of nuclei including the caudate nucleus and the putamen, as well as the nucleus accumbens. The caudate nucleus and putamen are often referred to as the dorsal striatum, and the nucleus accumbens as the ventral striatum.

Subarachnoid space: A cerebrospinal fluid (CSF)-filled space between the pial and arachnoid layers of the meninges. This space surrounds the brain.

Subiculum: A region of the hippocampal formation.

Superior Colliculi: Paired nuclei on the superior aspect of the midbrain involved in redirection of gaze and attention. The inferior and superior colliculi together comprise the midbrain tectum.

Tectum: A collective term for the superior and inferior colliculi of the brainstem.

Thalamus: A complex collection of nuclei in the diencephalon principally involved in the relay of signals to and from the cerebral cortex.

Third Ventricle: A midline cerebrospinal fluid (CSF)-filled space in the diencephalon. The third ventricle is connected with both left and right lateral ventricles via the foramen of Monro, as well as the fourth ventricle via the cerebral aqueduct.

Transverse Cerebral Fissure: A specific extension of the subarachnoid space between the thalami and fornices.

Uncus: The medial-most bulge of the medial temporal lobe. The uncus is visible from an inferior perspective of the temporal lobe.

Ventral Diencephalon: A region inferior to the thalamus extending from the anterior commissure to the posterior commissure. This region includes gray matter nuclei such as the hypothalamus, subthalamus, habenular nucleus, and red nucleus, and white matter pathways such as regions of the internal capsule, regions of the fornix, and the optic tract.
