# Supplementary material for: Anatomically curated segmentation of human subcortical structures in high resolution magnetic resonance imaging: An open science approach
Source: Front Neuroanat. 2022 Sep 30;16:894606. doi: 10.3389/fnana.2022.894606 (PMC9562126; doi:10.3389/fnana.2022.894606)
Supplement: Supplementary file 2 [file Data_Sheet_2.pdf]

Lateral Ventricle

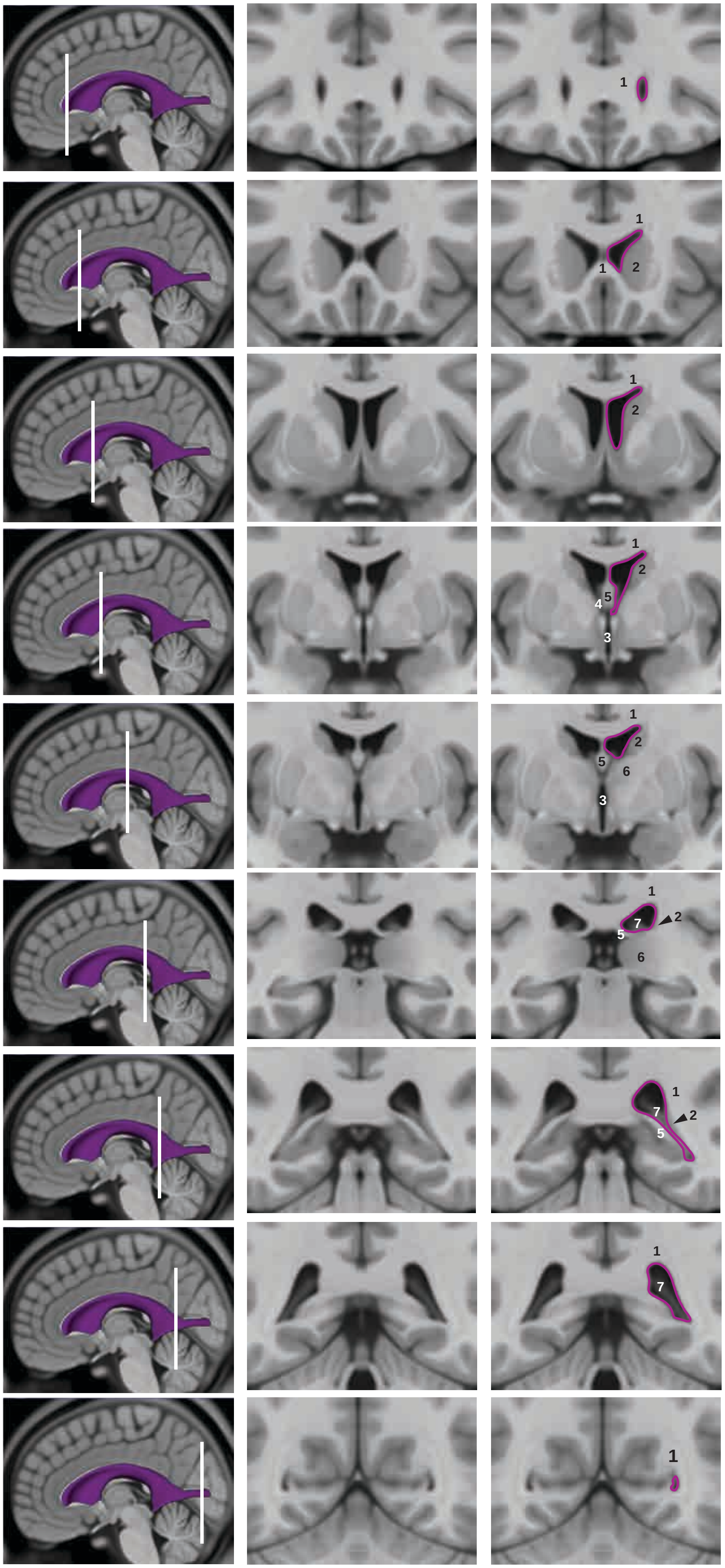

1. White Matter
2. Caudate
3. Third Ventricle
4. Foramen of Monro
5. Fornix
6. Thalamus
7. Choroid Plexus (in ventricle)

The Third Ventricle

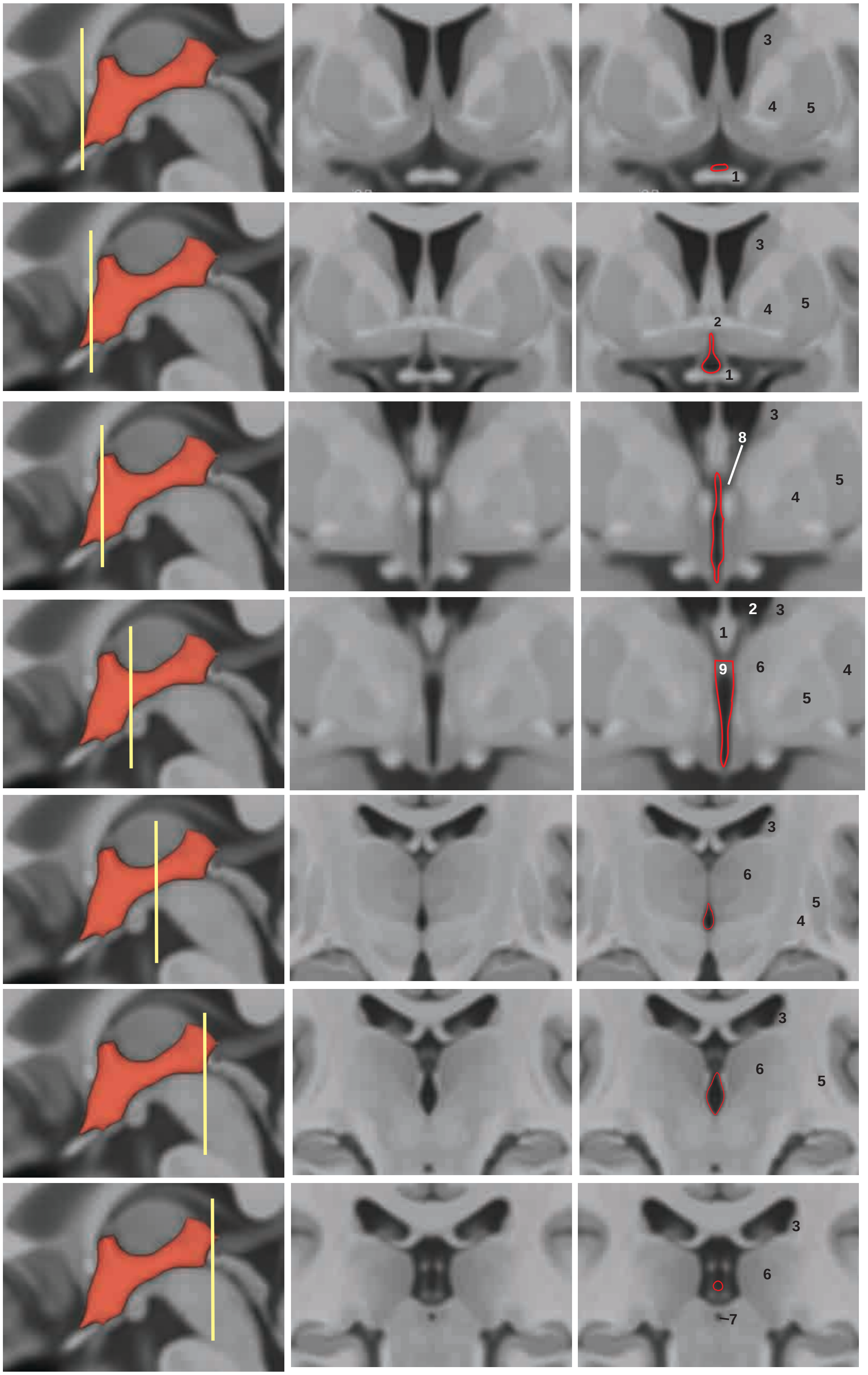

1. Optic Chiasm
2. Anterior Commissure
3. Caudate
4. Globus Pallidus
5. Putamen
6. Thalamus
7. Cerebral Aqueduct
8. Foramen of Monro
9. Choroid plexus

The Transverse Cerebral Fissure

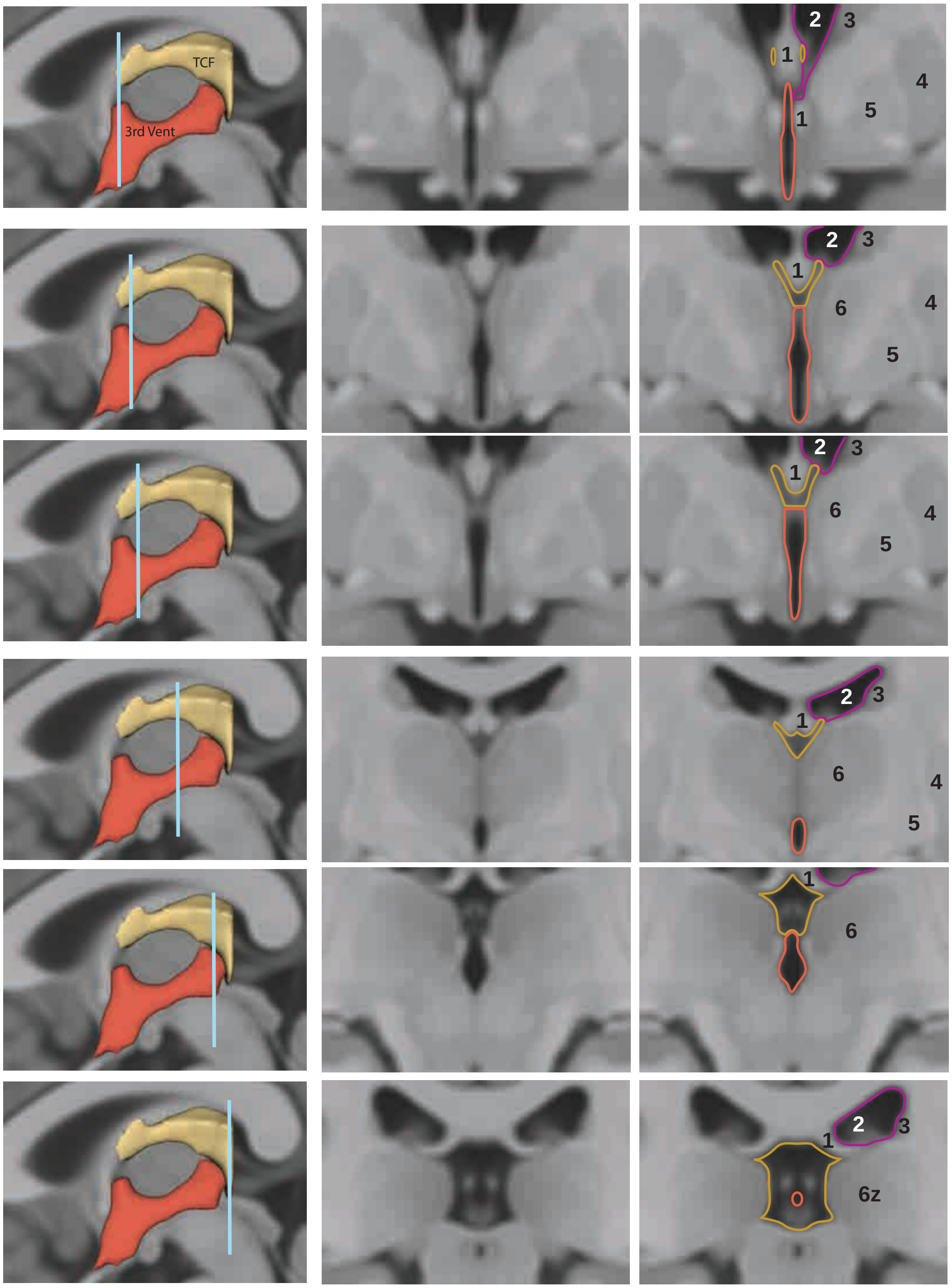

1. Fornix
2. Lateral Ventricle
3. Caudate
4. Putamen
5. Globus Pallidus
6. Thalamus

The Fourth Ventricle

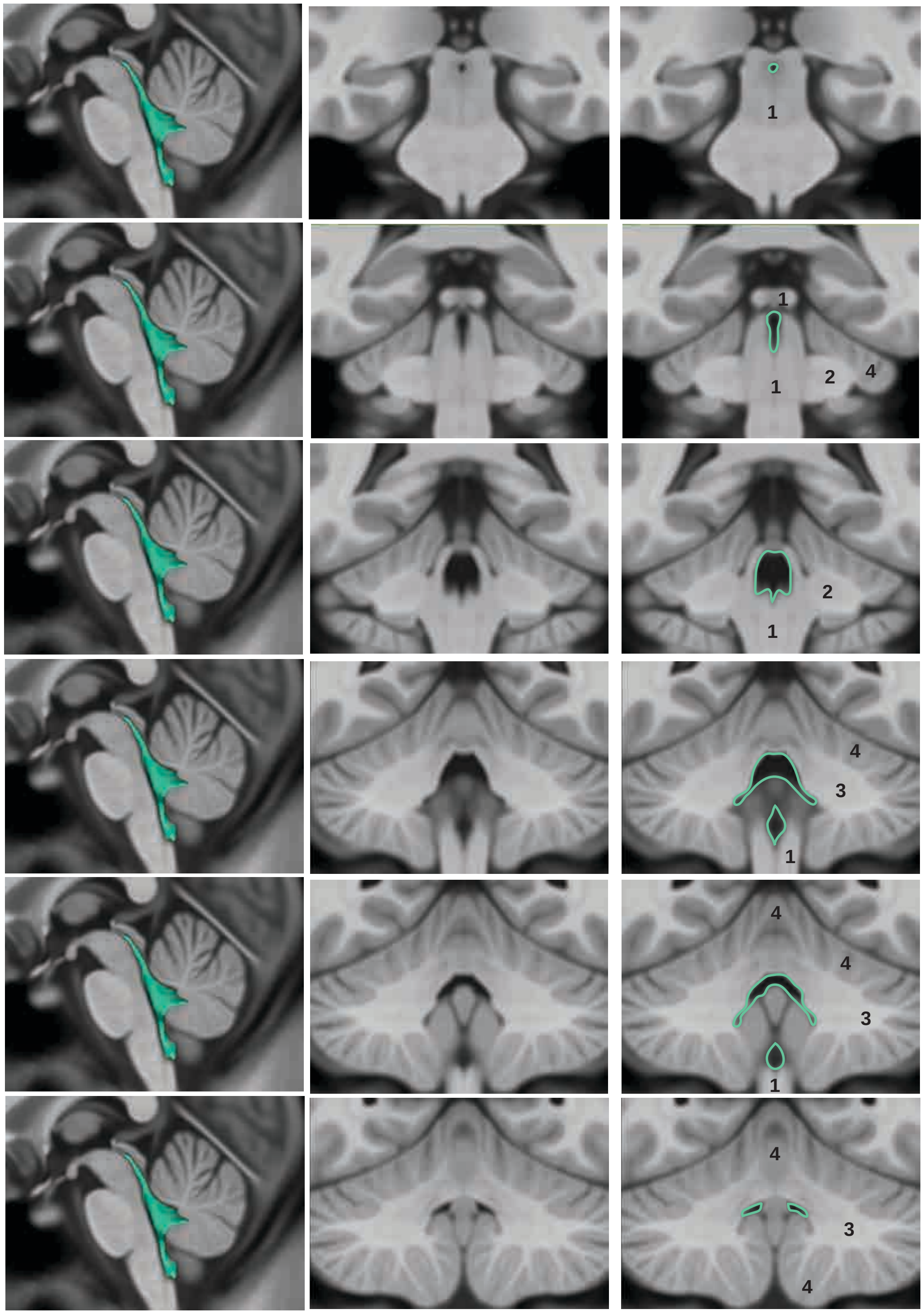

1. Brainstem
2. Middle Cerebellar Peduncle
3. Cerebellar White Matter
4. Cerebellar Cortex

Nucleus Accumbens

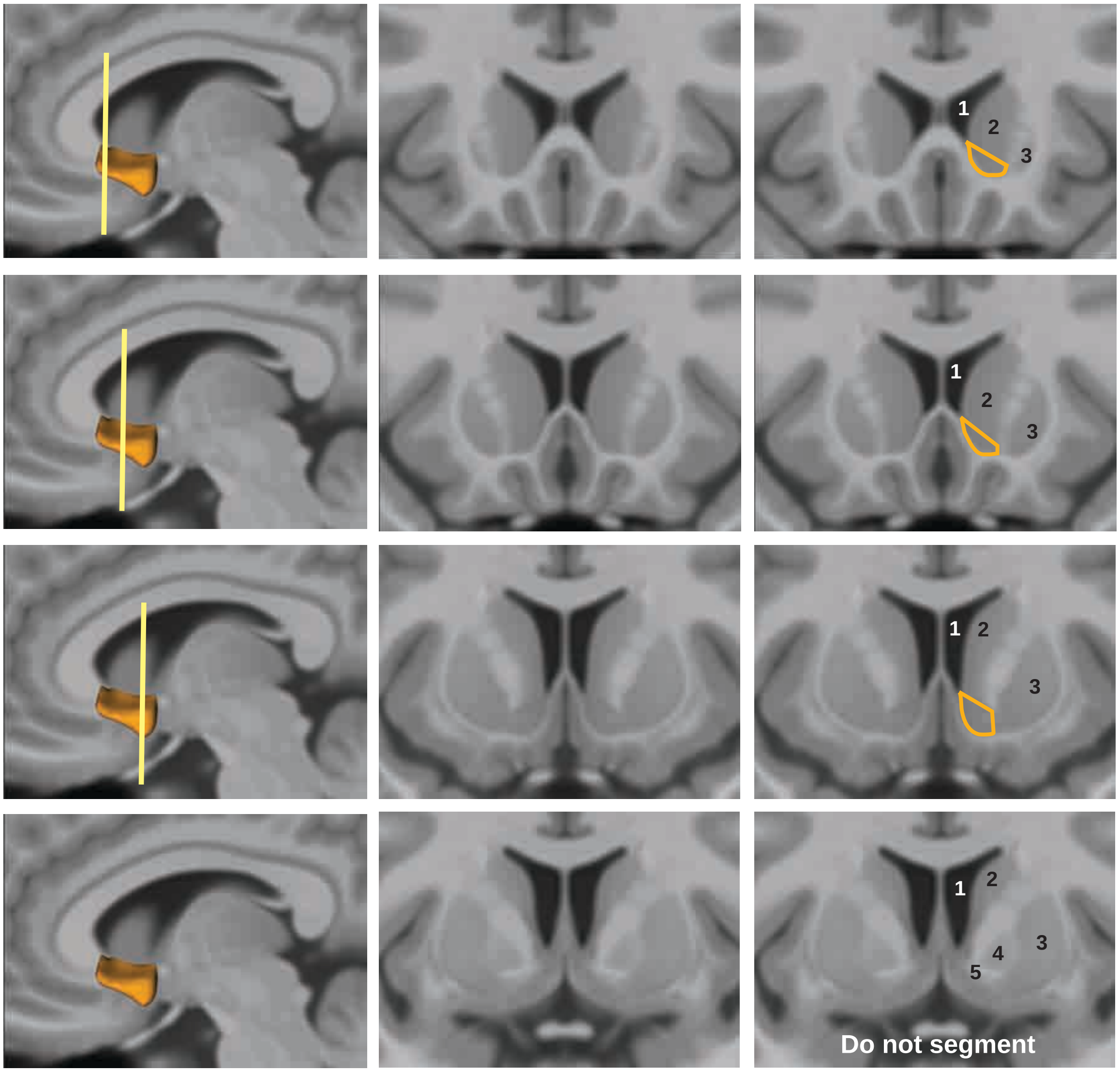

1. Lateral Ventricle
2. Caudate
3. Putamen
4. Globus Pallidus
5. Anterior Commissure

The Caudate

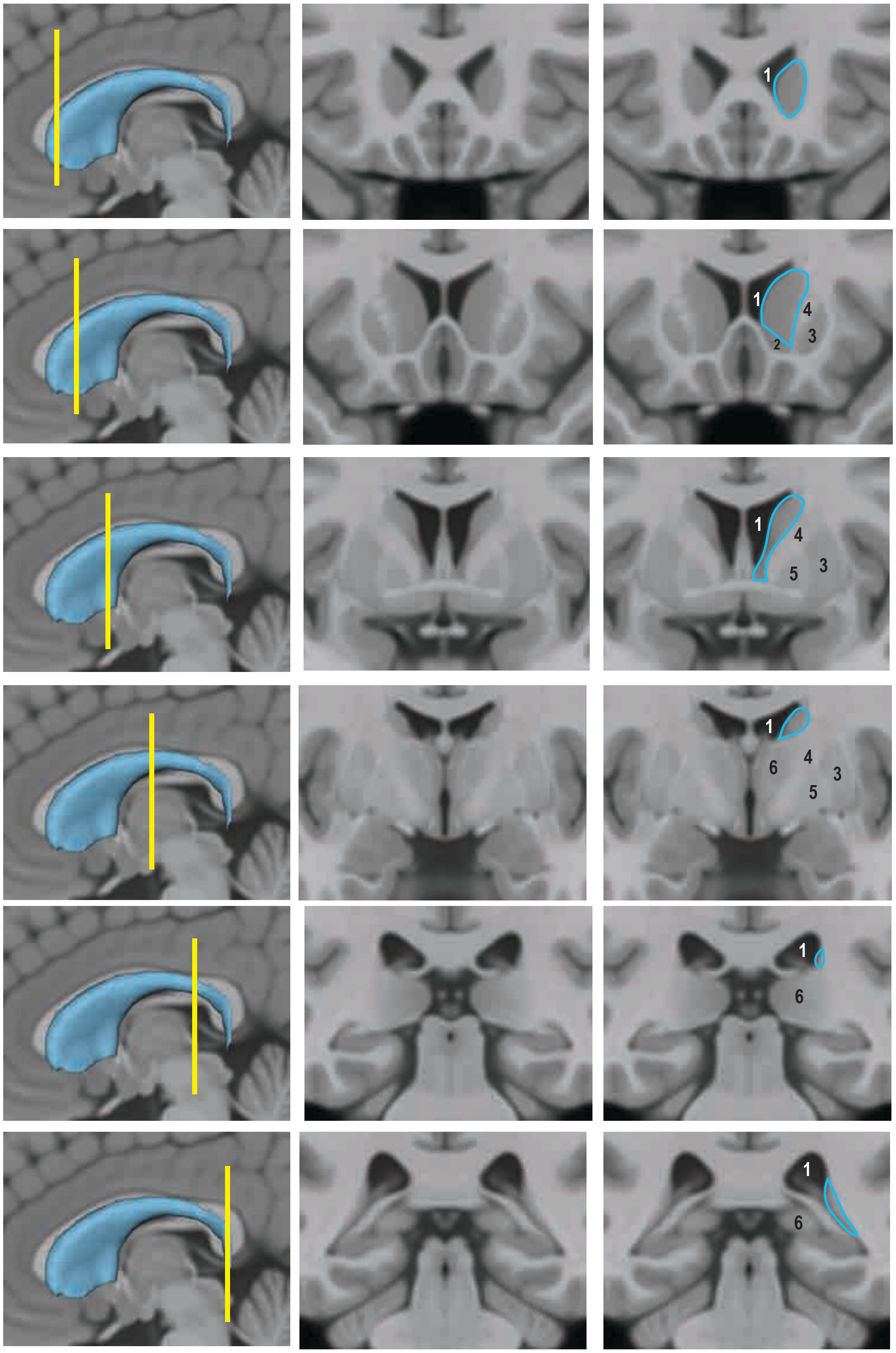

1. Lateral Ventricle
2. Nucleus Accumbens
3. Putamen
4. Internal Capsule
5. Globus Pallidus
6. Thalamus

The Putamen

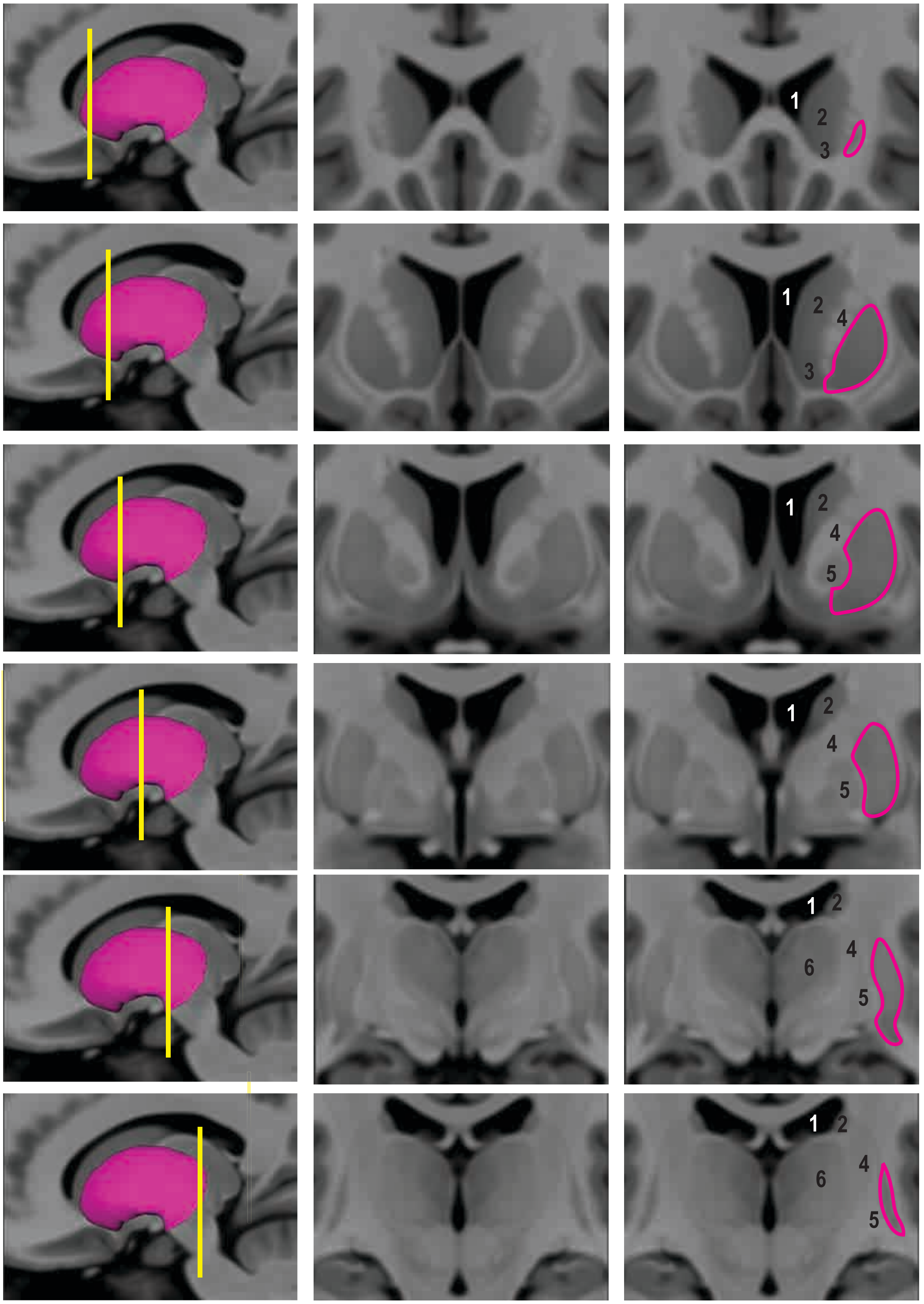

1. Lateral Ventricle
2. Caudate
3. Nucleus Accumbens
4. Internal Capsule
5. Globus Pallidus
6. Thalamus

Globus Pallidus

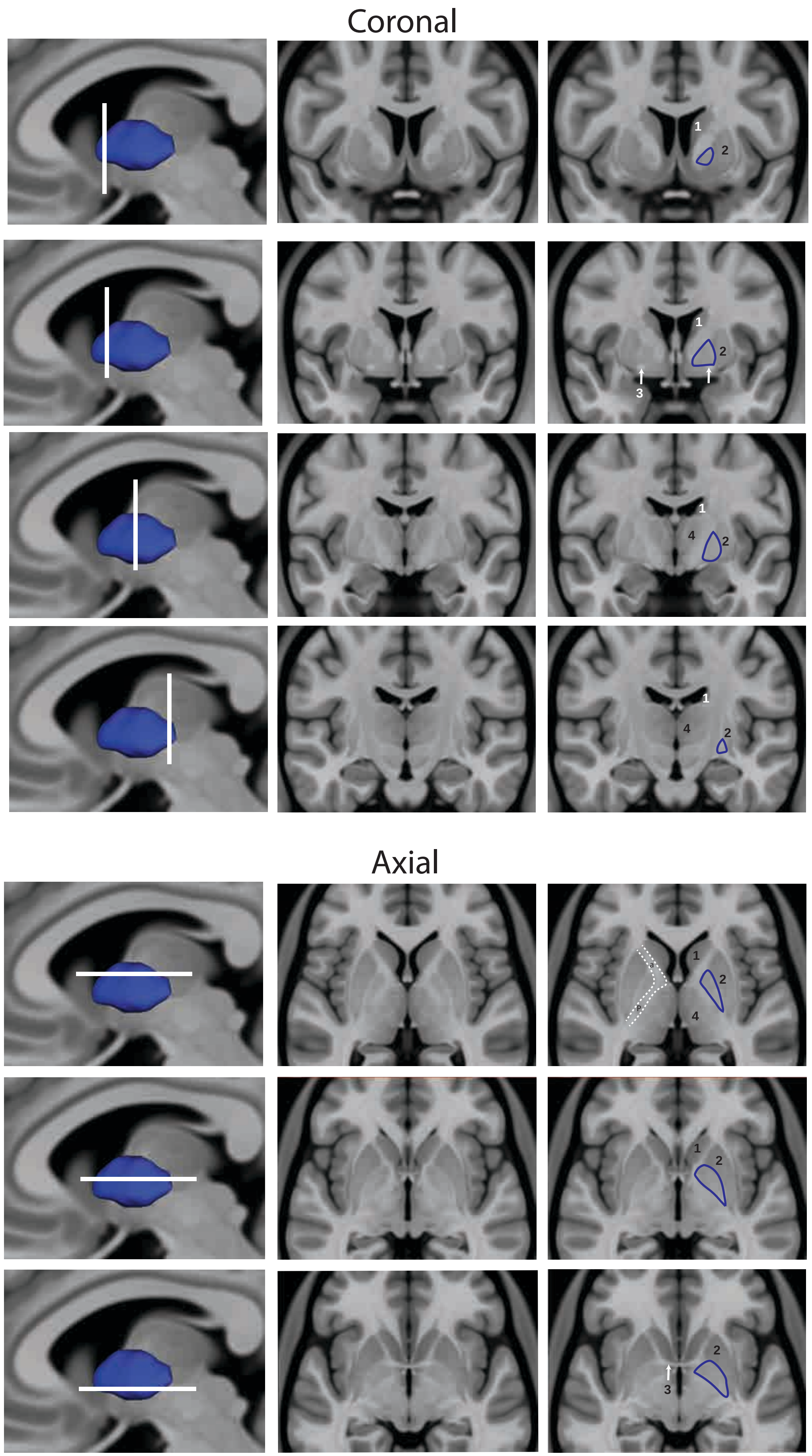

1. Caudate
2. Putamen
3. Anterior Commissure
4. Thalamus

a. Anterior limb of the internal capsule
p. Posterior limb of the internal capsule

Brainstem

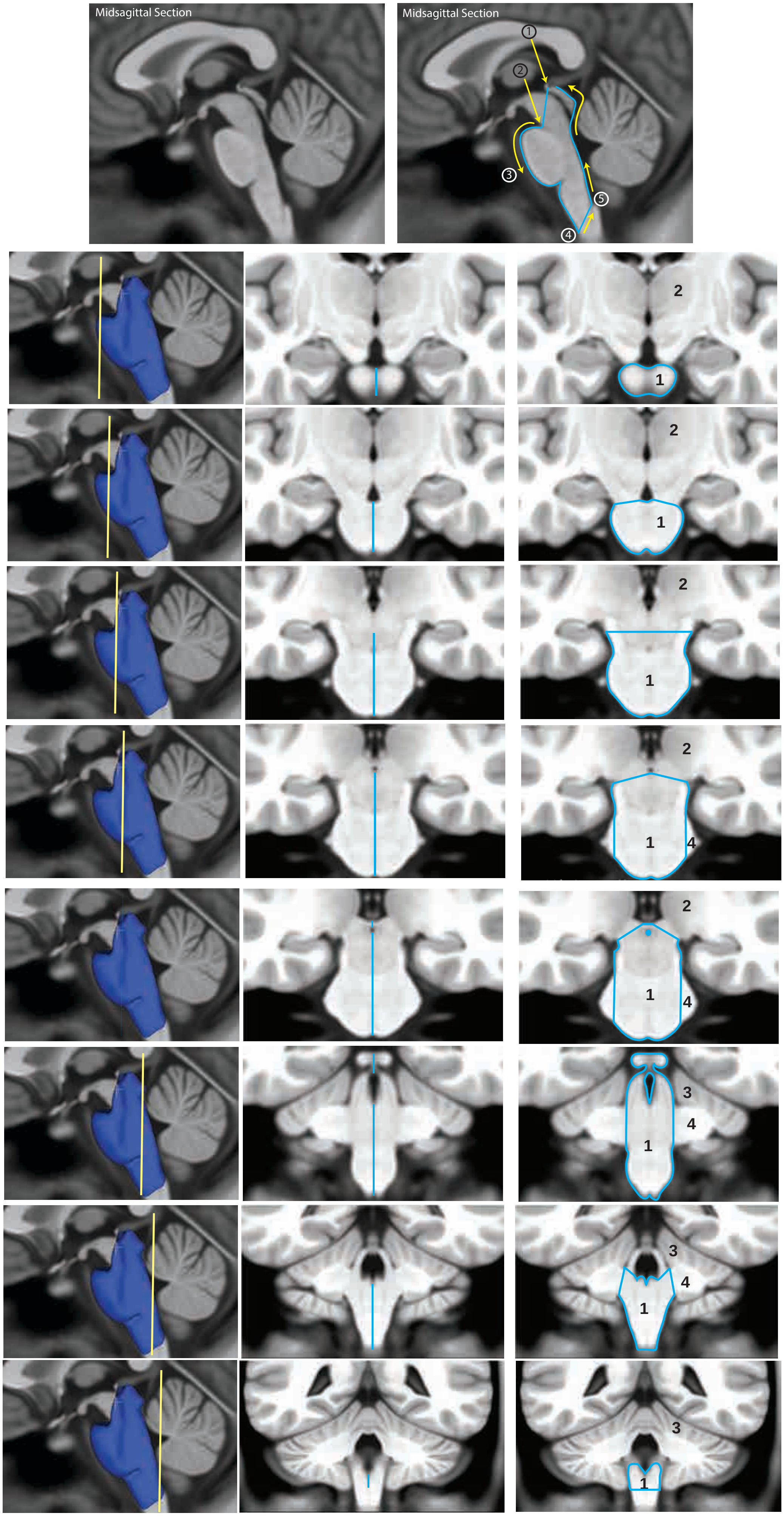

1. Brainstem
2. Thalamus
3. Cerebellum
4. Middle Cerebellar Peduncle

The Thalamus

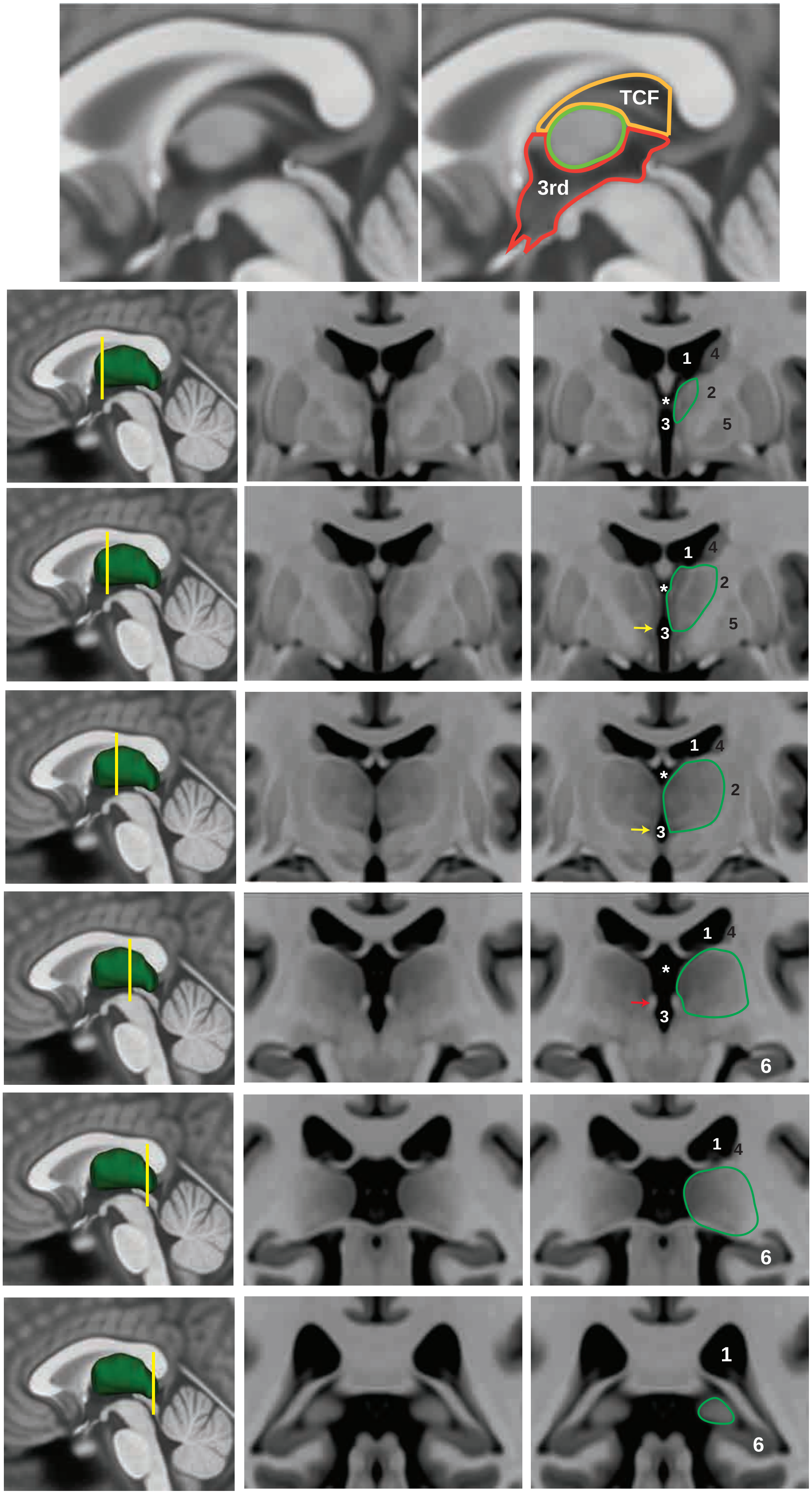

1. Lateral Ventricle
2. Internal Capsule
3. Third Ventricle
4. Caudate
5. Globus Pallidus
6. Hippocampus

*. Transverse Cerebral Fissure
yellow arrow - hypothalamic sulcus
red arrow - habenular complex

The Ventral Diencephalon

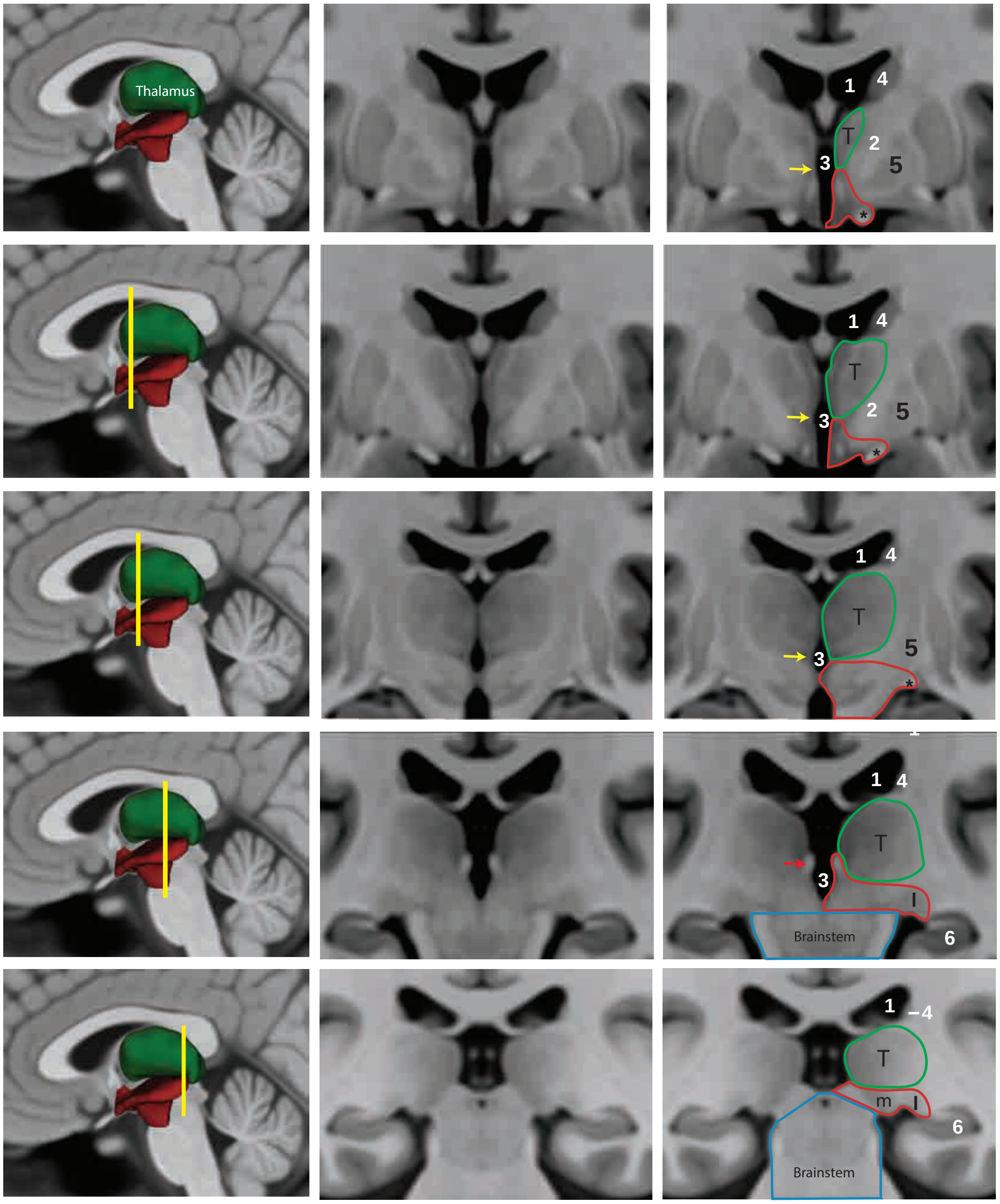

1. Lateral Ventricle
2. Internal Capsule
3. Third Ventricle
4. Caudate
5. Globus Pallidus
6. Hippocampus

*. Optic Tract

m. medial geniculate nucleus

l. lateral geniculate nucleus

T. Thalamus

yellow arrow - hypothalamic sulcus

red arrow - habenular complex

Amygdala

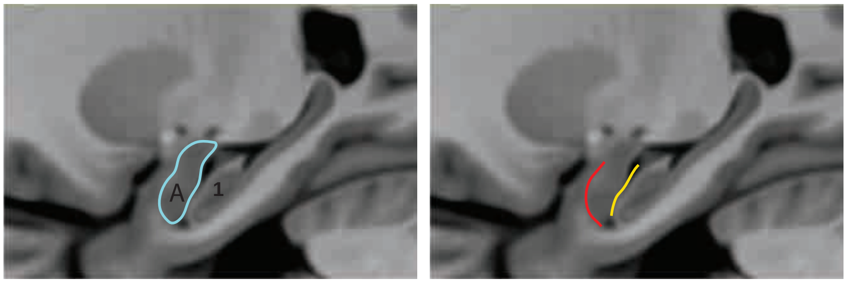

The use of multiple guide markup lines placed between the amygdala (A) and the hippocampus (yellow) and between the anterior amygdala and the white matter (red) in the sagittal plane aids in the anatomically-reliable identification of these borders.

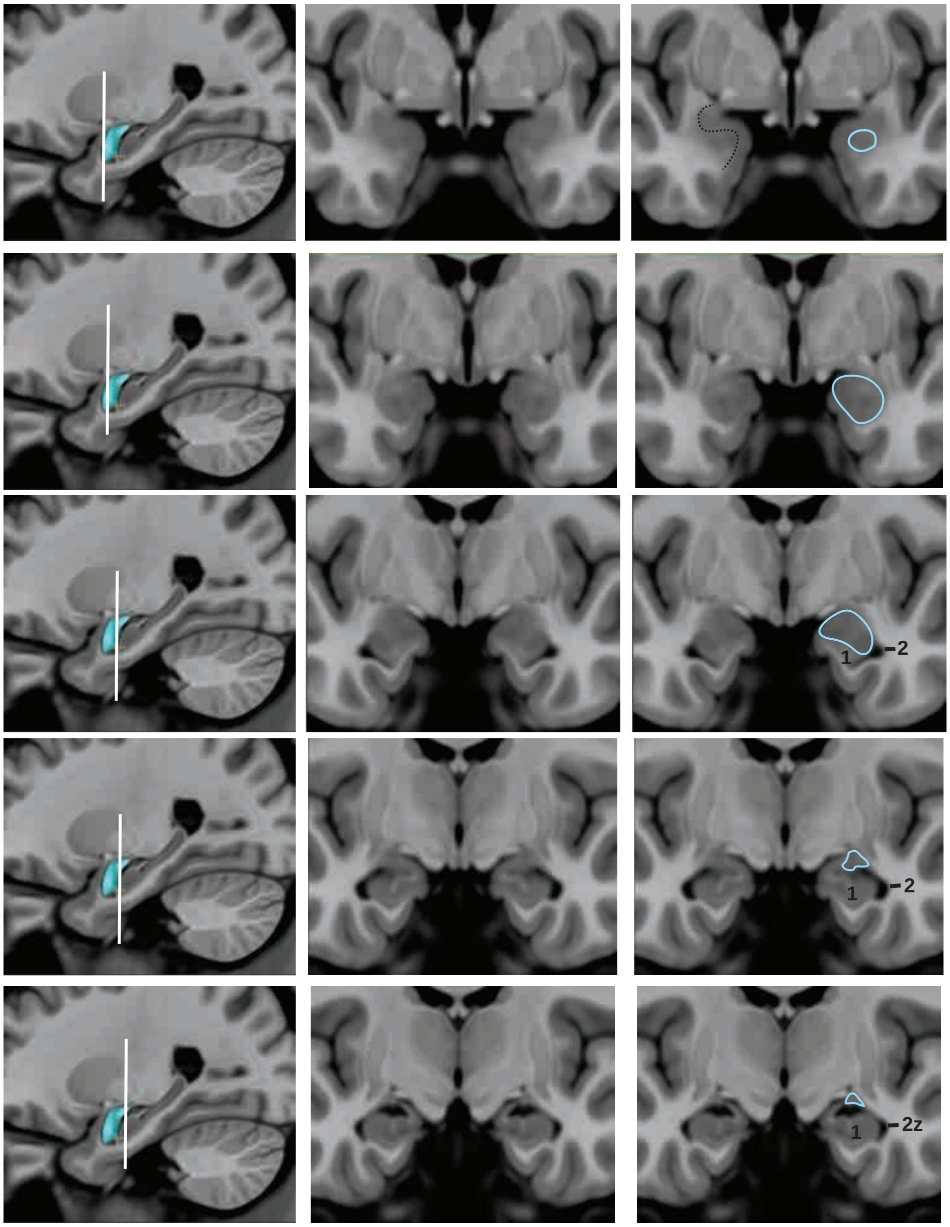

1. Hippocampus
2. Inf Horn Lat Vent

The Hippocampal Formation

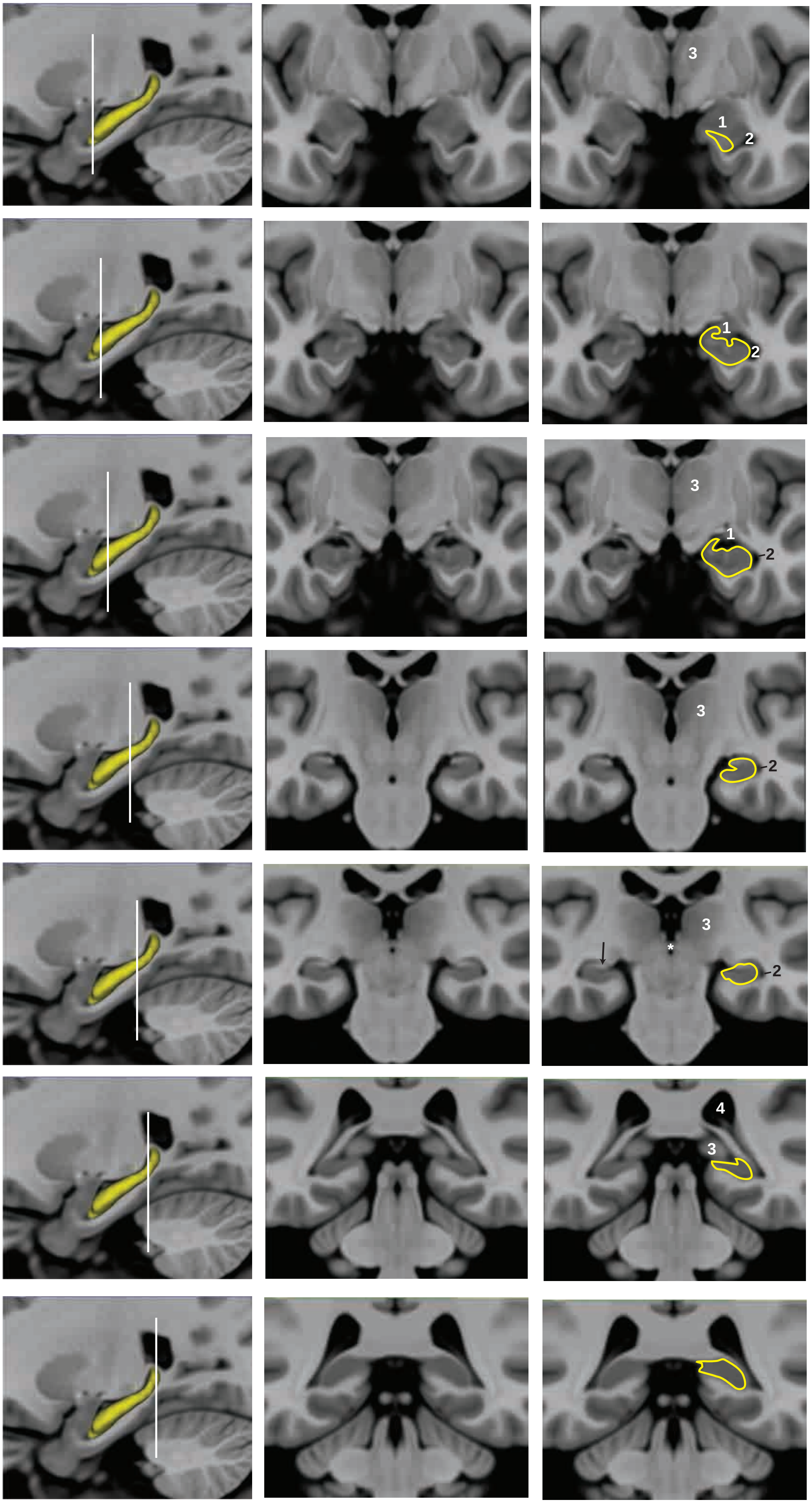

1. Amygdala
2. Inf. Horn of Lat Ventricle
3. Thalamus
4. Atrium of Lat Ventricle

IMPORTANT CONVENTION: In coronal sections posterior to the posterior commissure (asterisk), do not include the white matter bundle adjacent to the hippocampal formation(dashed line at tip of black arrow). In sections anterior to and including the posterior commissure, include the bundle in the hippocampal formation segmentation.

Inferior Horn of the Lateral Ventricle

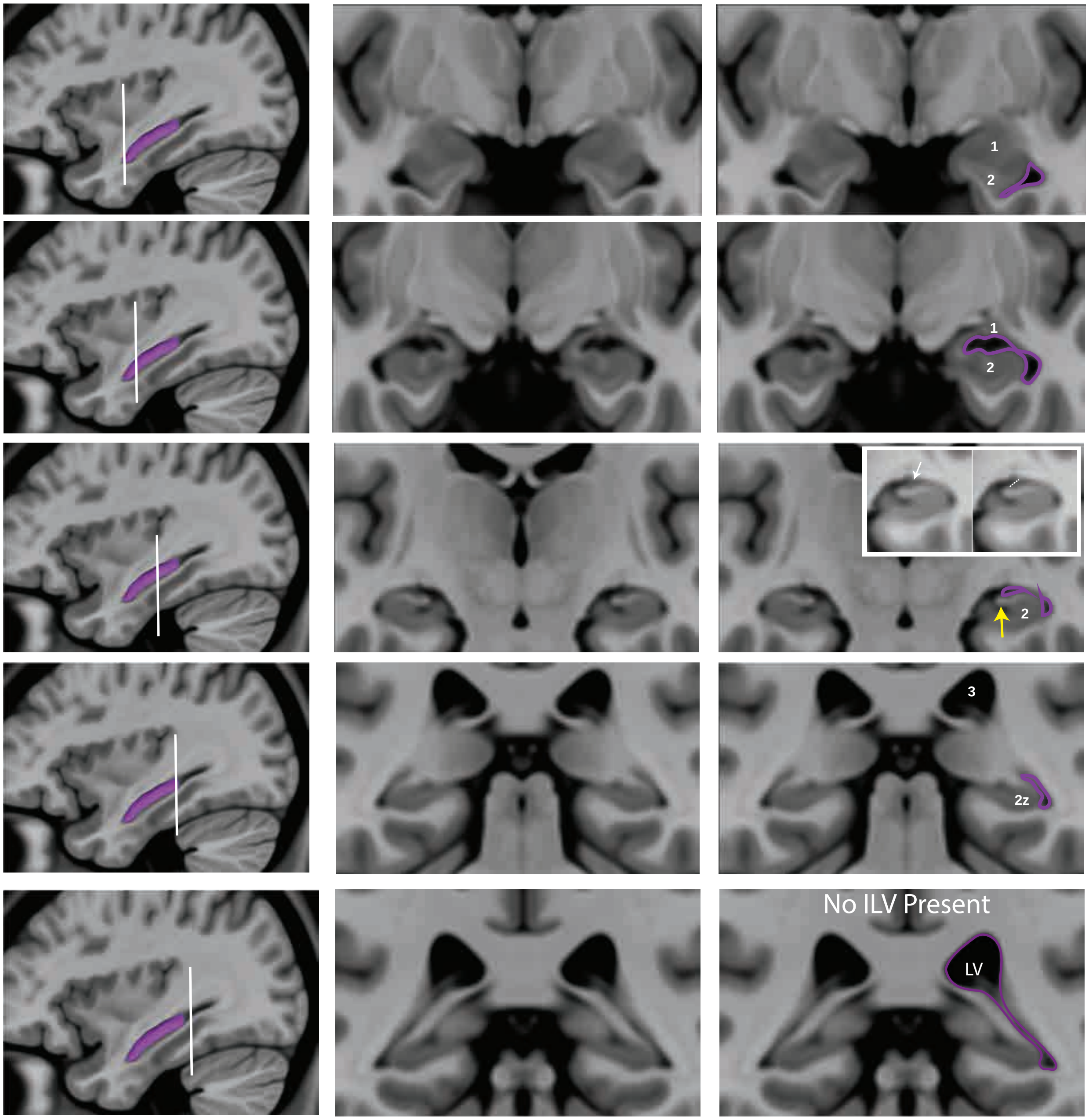

1. Amygdala
2. Hippocampus
3. Lat Ventricle Body

Fifth Ventricle

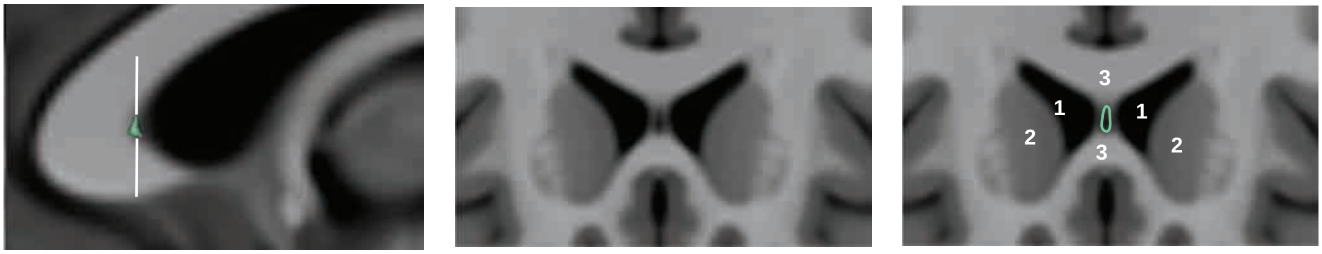

1. Lateral Ventricle
2. Caudate
3. Corpus Callosum

The Optic Chiasm

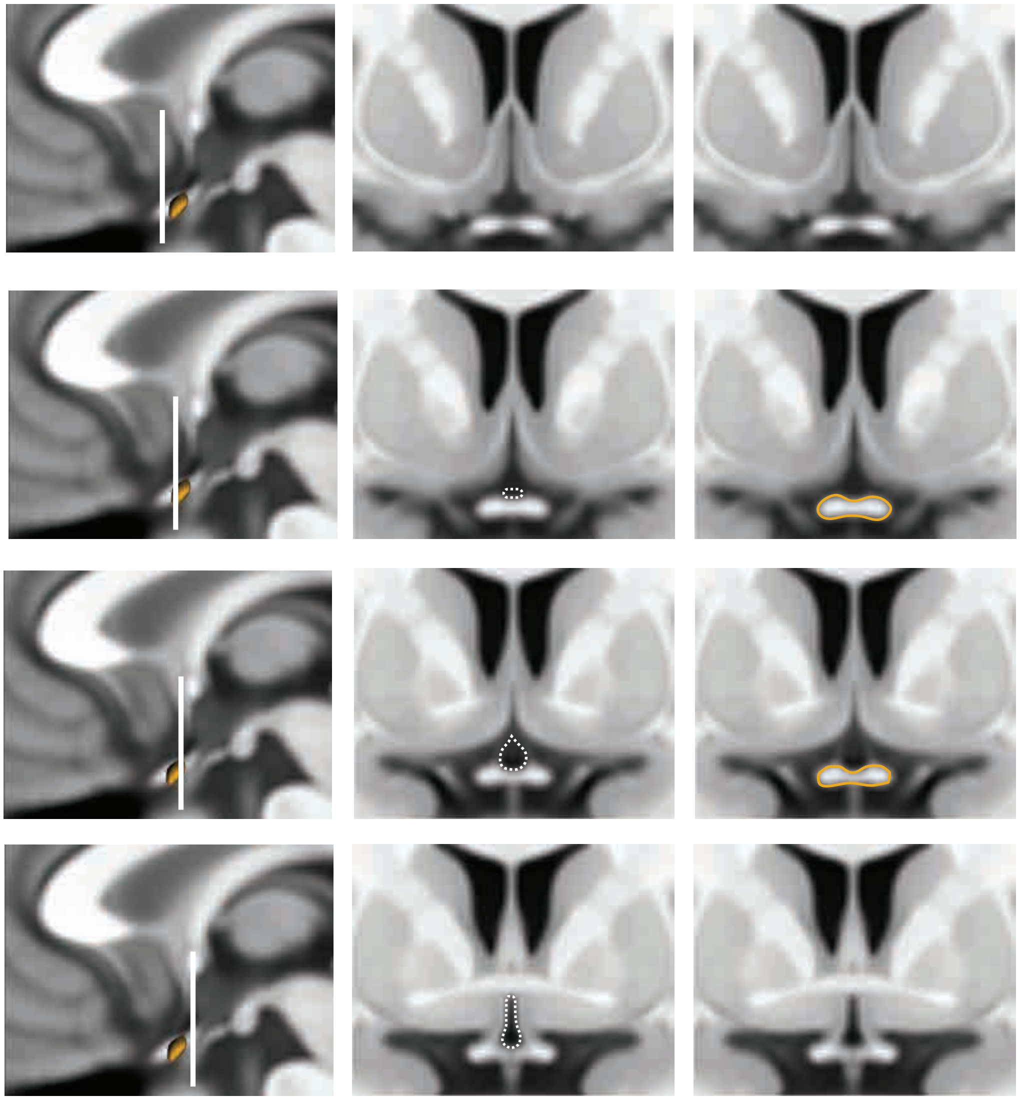

The optic chiasm is segmented at the first section that the third venticle (white dotted line) appears. Segmentation of the optic chiasm ends when the chiasm clearly separates into two optic tracts.
