# Supplementary material for: Anatomically curated segmentation of human subcortical structures in high resolution magnetic resonance imaging: An open science approach
Source: Front Neuroanat. 2022 Sep 30;16:894606. doi: 10.3389/fnana.2022.894606 (PMC9562126; doi:10.3389/fnana.2022.894606)
Supplement: Supplementary file 3 [file Data_Sheet_3.pdf]

Brainstem

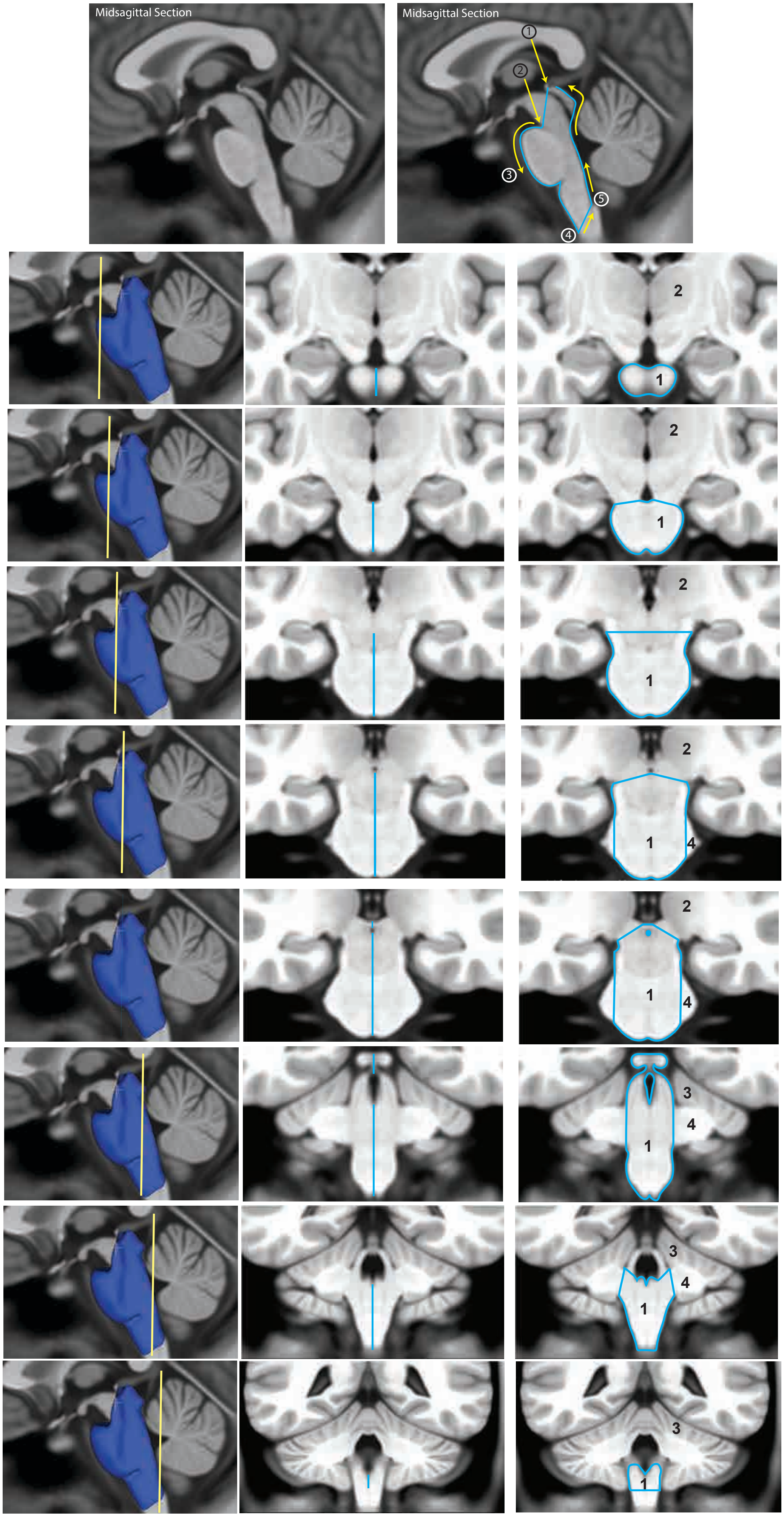

1. Brainstem
2. Thalamus
3. Cerebellum
4. Middle Cerebellar Peduncle

The Thalamus

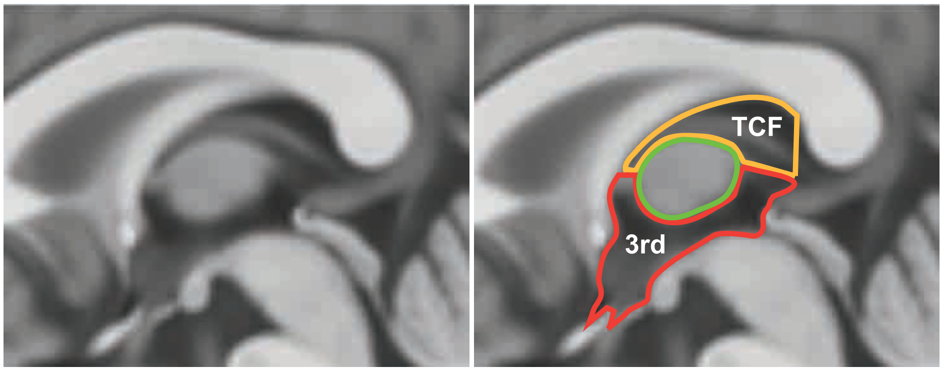

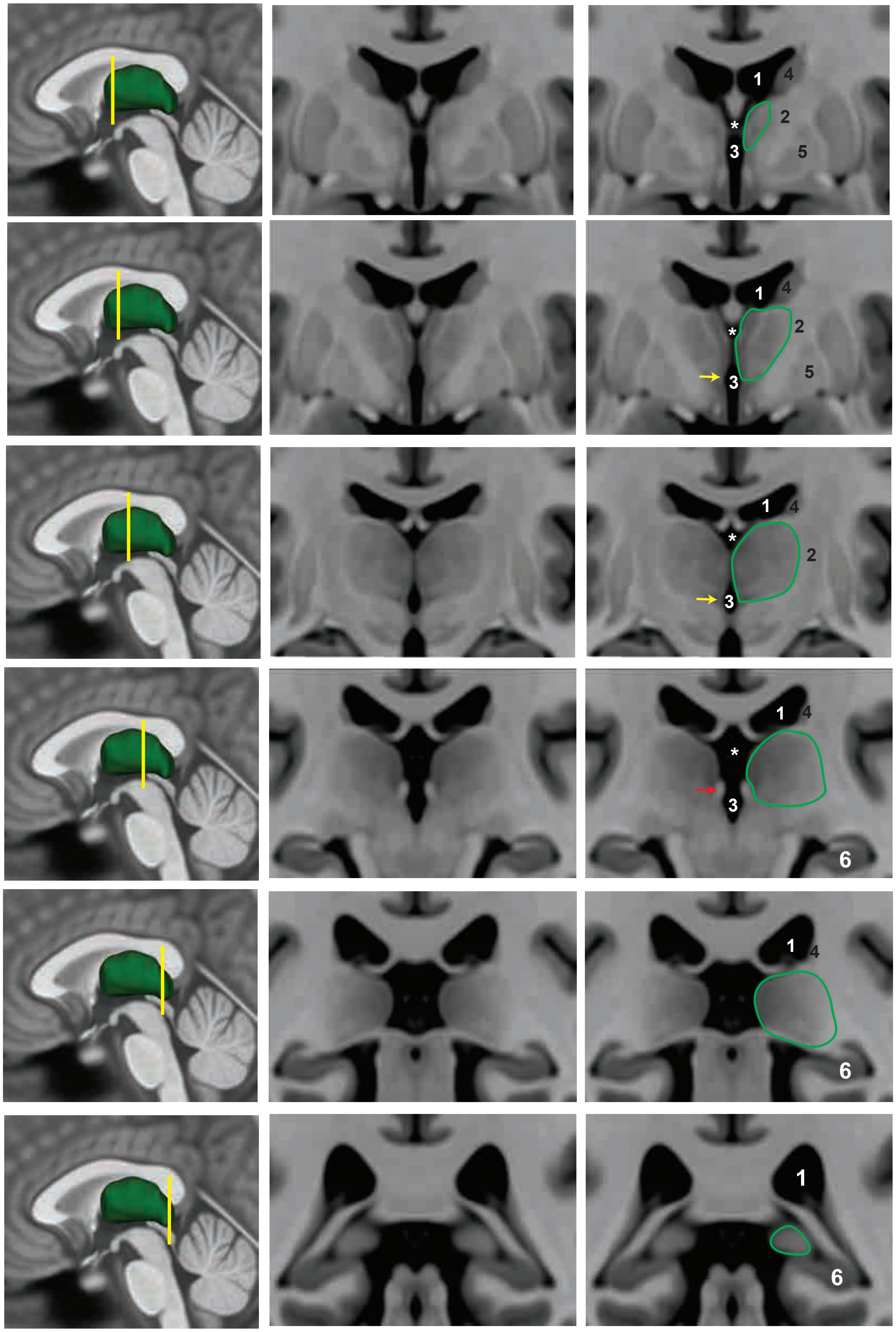

1. Lateral Ventricle
2. Internal Capsule
3. Third Ventricle
4. Caudate
5. Globus Pallidus
6. Hippocampus
*. Transverse Cerebral Fissure

The Ventral Diencephalon

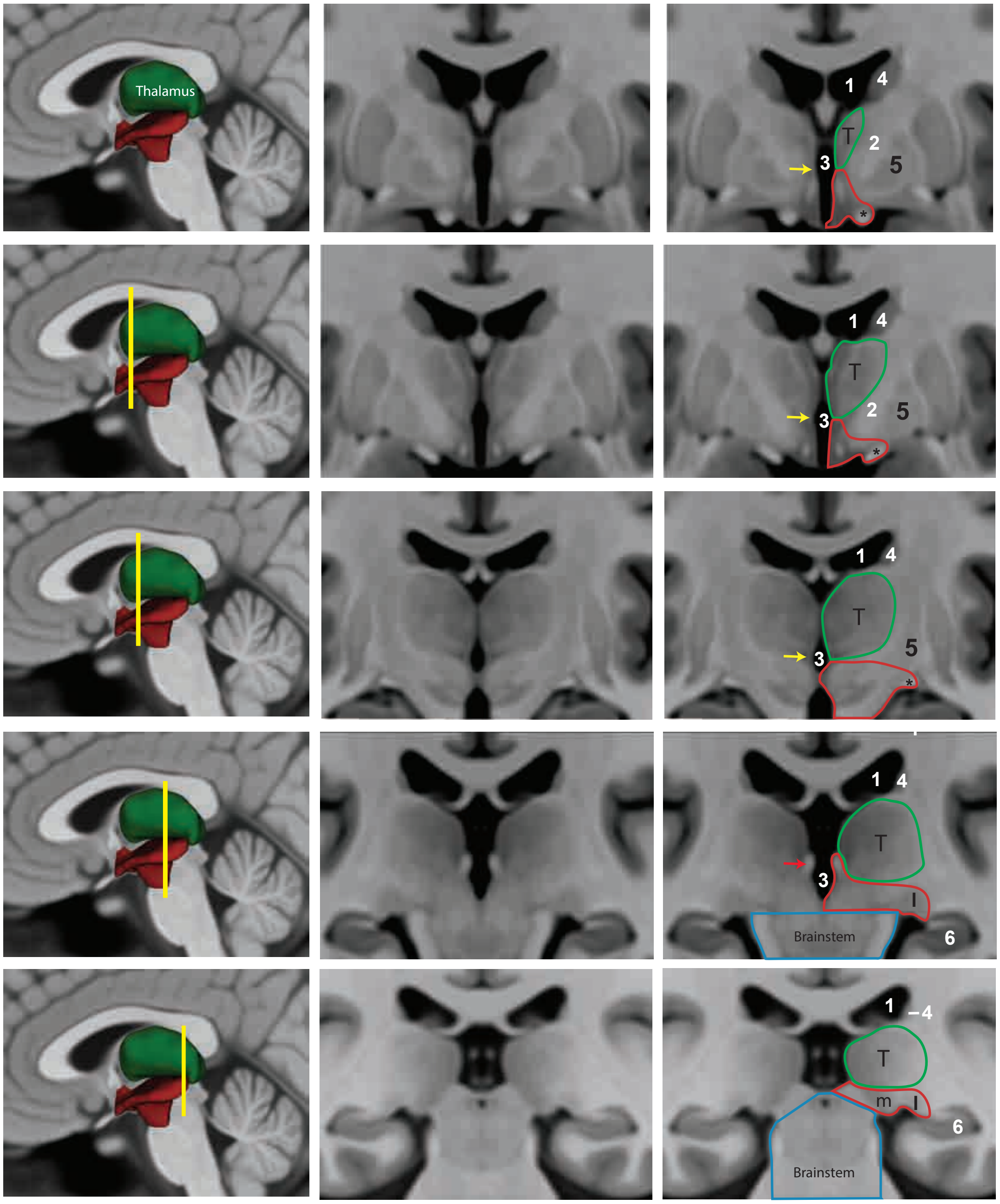

1. Lateral Ventricle
2. Internal Capsule
3. Third Ventricle
4. Caudate
5. Globus Pallidus
6. Hippocampus

*. Optic Tract

m. medial geniculate nucleus

l. lateral geniculate nucleus

T. Thalamus

Inferior Horn of the Lateral Ventricle

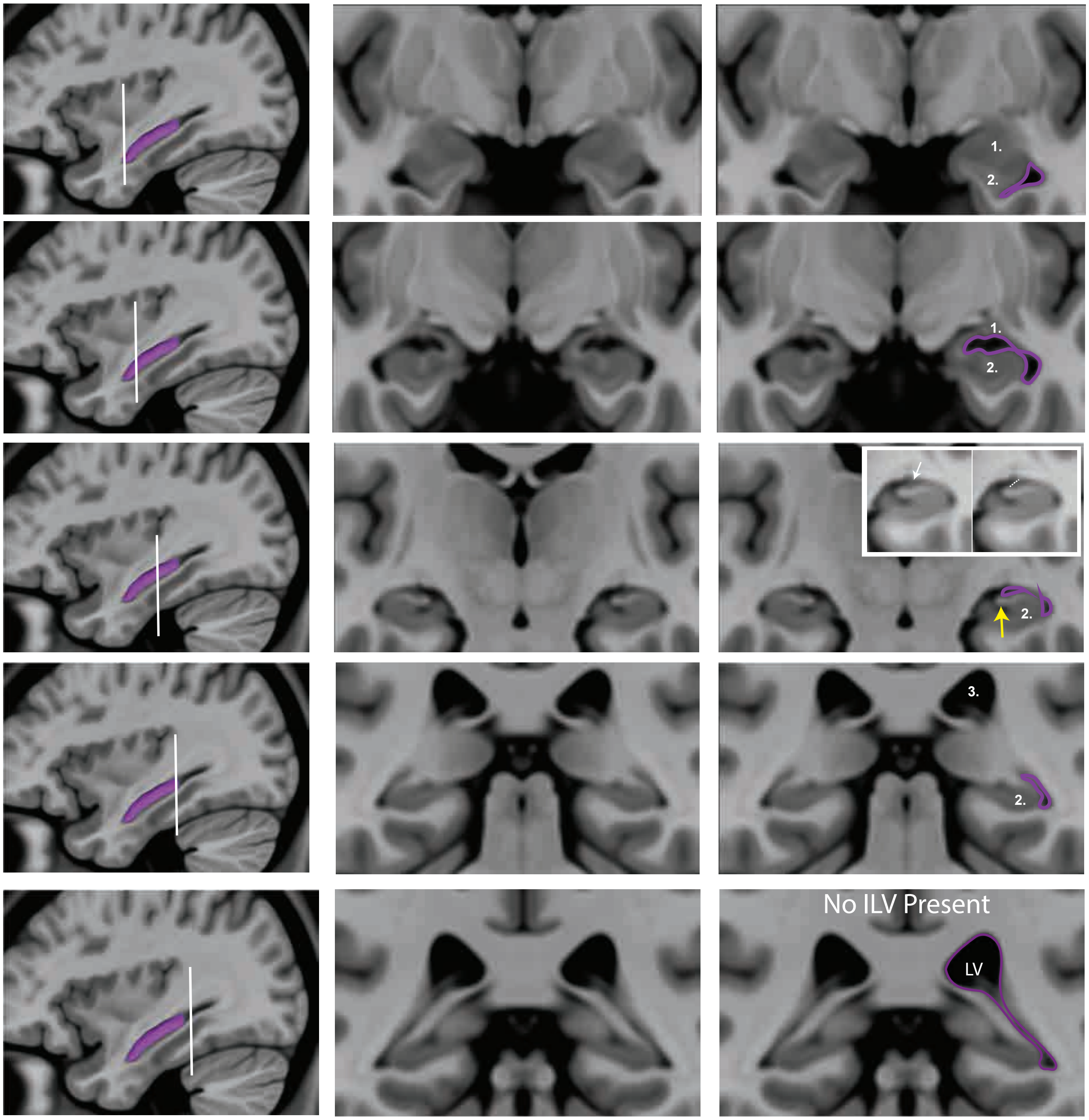

1. Amygdala
2. Hippocampus
3. Lat Ventricle Body

The Hippocampal Formation

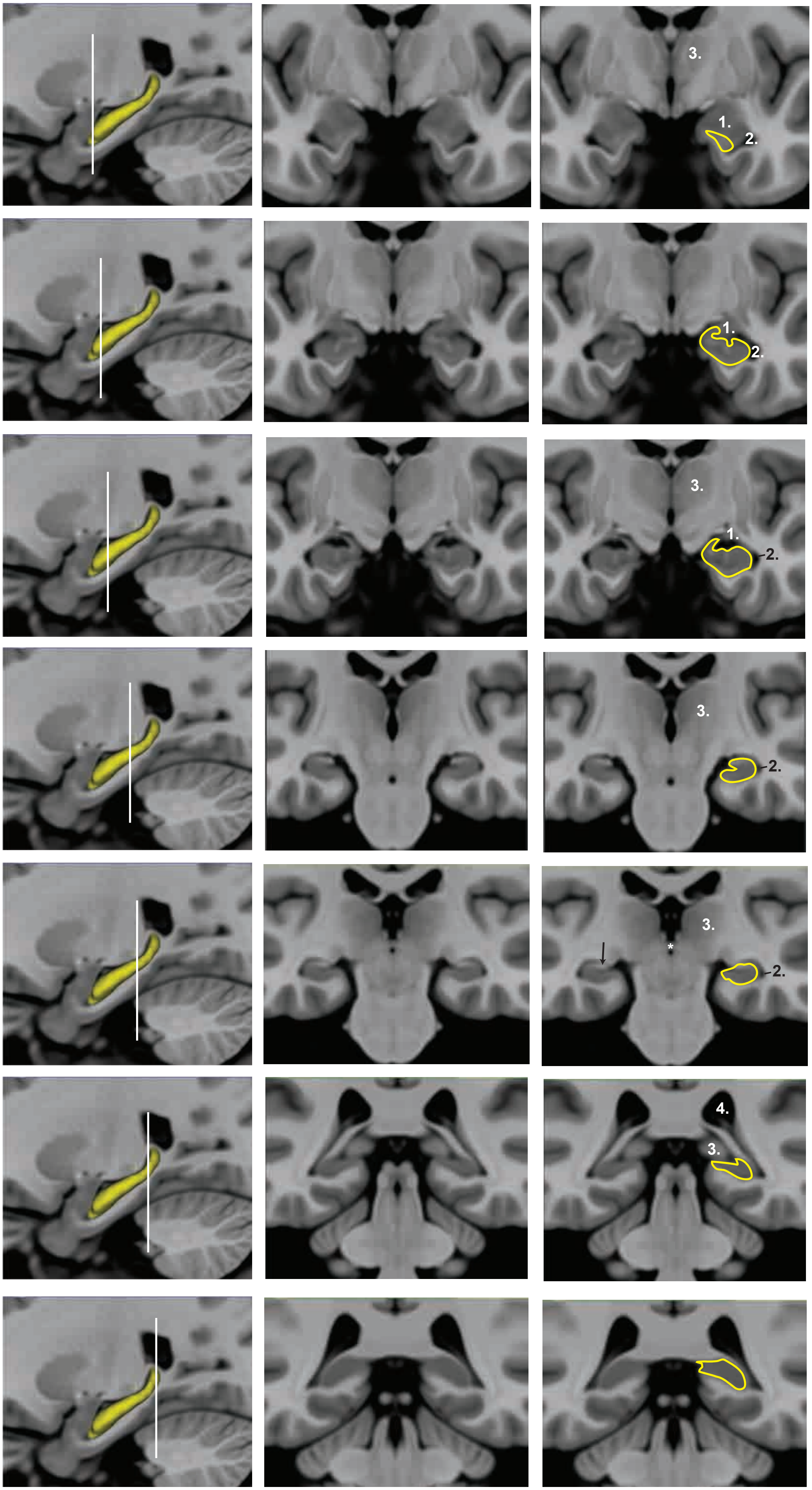

1. Amygdala
2. Inf. Horn of Lat Ventricle
3. Thalamus
4. Atrium of Lat Ventricle

IMPORTANT CONVENTION: In coronal sections posterior to the posterior commissure (asterisk), do not include the white matter bundle adjacent to the hippocampal formation(dashed line at tip of black arrow). In sections anterior to and including the posterior commissure, include the bundle in the hippocampal formation segmentation.

Amygdala

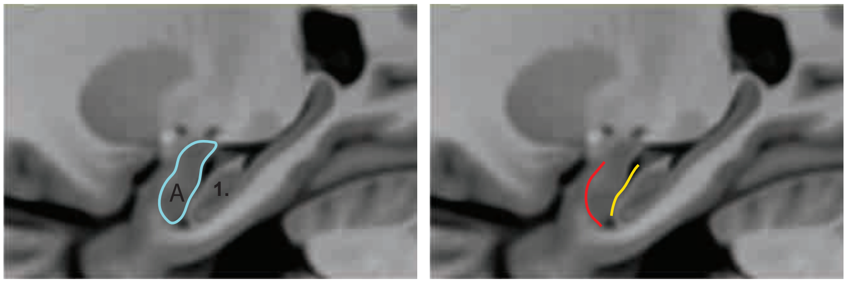

The use of multiple guide markup lines placed between the amygdala and the hippocampus (yellow) and between the anterior amygdala and the white matter (red) in the sagittal plane aids in the anatomically-reliable identification of these borders.

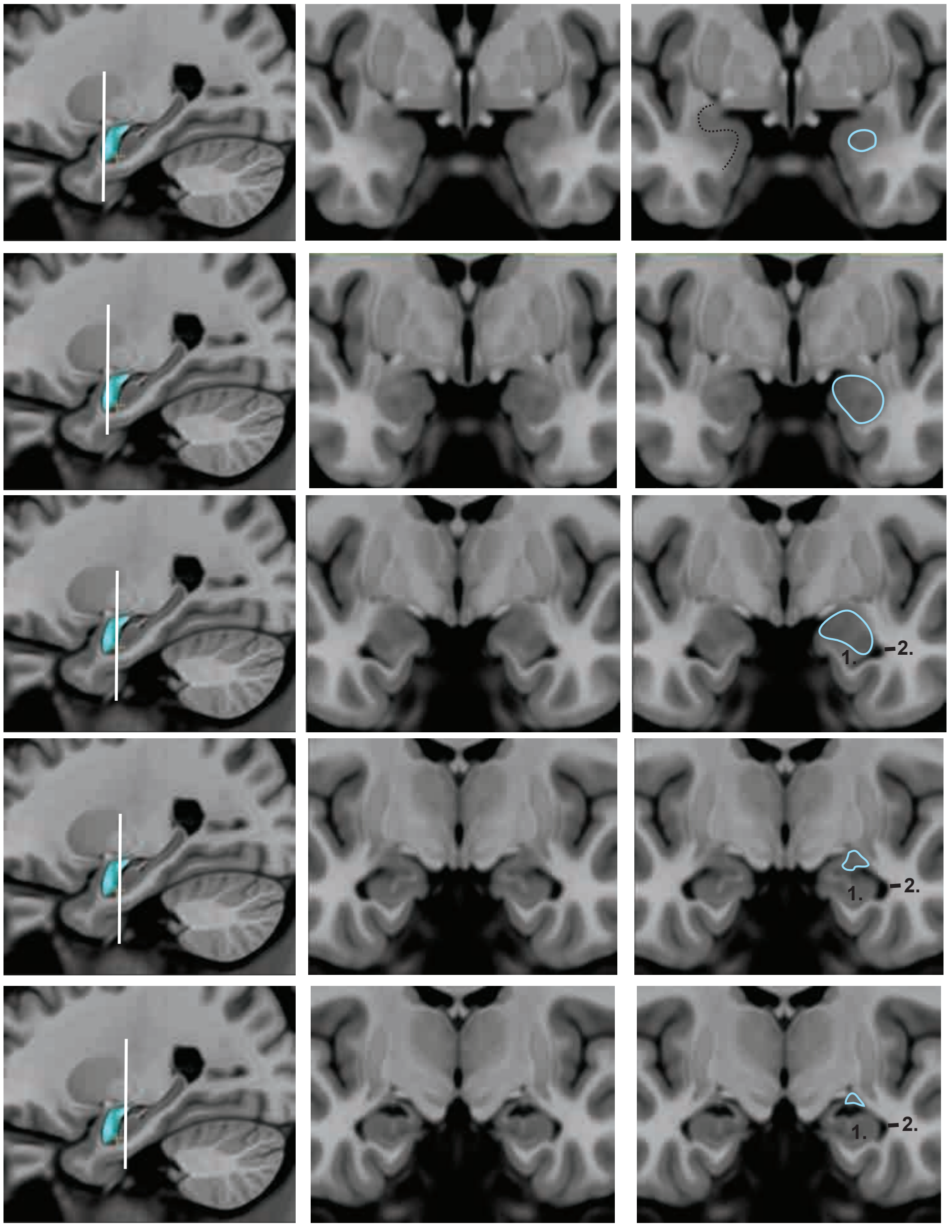

1. Hippocampus
2. Inf Horn Lat Vent

Fifth Ventricle

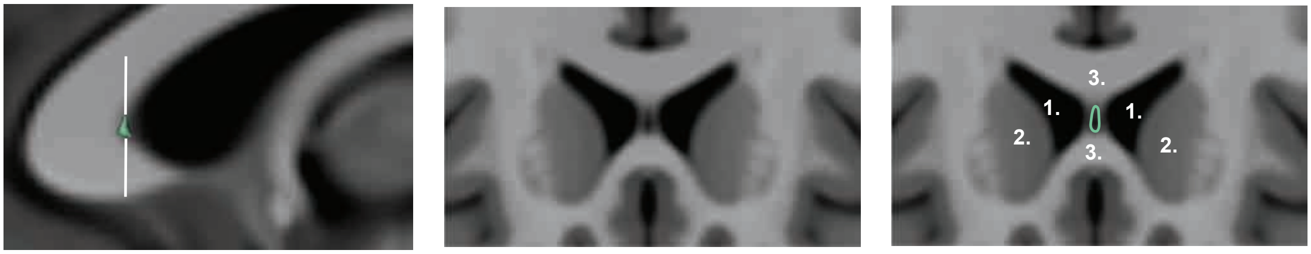

1. Lateral Ventricle
2. Caudate
3. Corpus Callosun
